# Transcriptional repression of *GIF1* by the KIX-PPD-MYC repressor complex controls seed size in Arabidopsis

**DOI:** 10.1038/s41467-020-15603-3

**Published:** 2020-04-15

**Authors:** Zupei Liu, Na Li, Yueying Zhang, Yunhai Li

**Affiliations:** 1 State Key Laboratory of Plant Cell and Chromosome Engineering, CAS Centre for Excellence in Molecular Plant Biology, Institute of Genetics and Developmental Biology, The Innovative Academy of Seed Design, Chinese Academy of Sciences, 100101 Beijing, China; 2 University of Chinese Academy of Sciences, 100039 Beijing, China

**Keywords:** Plant sciences, Seed development

## Abstract

Seed size is a key agronomic trait that greatly determines plant yield. Elucidating the molecular mechanism underlying seed size regulation is also an important question in developmental biology. Here, we show that the KIX-PPD-MYC-GIF1 pathway plays a crucial role in seed size control in *Arabidopsis thaliana*. Disruption of KIX8/9 and PPD1/2 causes large seeds due to increased cell proliferation and cell elongation in the integuments. KIX8/9 and PPD1/2 interact with transcription factors MYC3/4 to form the KIX-PPD-MYC complex in Arabidopsis. The KIX-PPD-MYC complex associates with the typical G-box sequence in the promoter of *GRF*-*INTERACTING FACTOR 1* (*GIF1*), which promotes seed growth, and represses its expression. Genetic analyses support that KIX8/9, PPD1/2, MYC3/4, and GIF1 function in a common pathway to control seed size. Thus, our results reveal a genetic and molecular mechanism by which the transcription factors MYC3/4 recruit KIX8/9 and PPD1/2 to the promoter of *GIF1* and repress its expression, thereby determining seed size in Arabidopsis.

## Abstract (web summary)

Seed size is an important determinant of plant yield. Here, Liu *et al*. show that a KIX-PPD repressor complex and MYC transcription factors interact with the G-box motif in the promoter of GRF-INTERACTING FACTOR 1 to regulate seed size by influencing cell proliferation and elongation in the integument.

## Introduction

Seed size is one of important agronomic traits that influences seed yield of crops, and elucidating the molecular mechanism underlying seed size control will help to improve seed yield. In flowering plants, a mature seed consists of the embryo, endosperm and seed coat. Seed development starts with double fertilisation. One sperm cell fuses with the egg cell to form the embryo, and another sperm cell fuses with the central cell to generate the endosperm. Thus, the embryo and endosperm are derived from the zygotic tissues. The seed coat, which envelops the embryo and the endosperm, is derived from maternal integuments^1–4^. Seed size is therefore coordinately determined by zygotic and maternal tissues. Several genes have been shown to control seed size in *Arabidopsis thaliana*, such as *DA1*, *ENHANCER OF DA1*-*1* (*EOD3*), *SUPPRESSOR OF DA1*-*1* (*SOD7*), *BIG BROTHER* (*BB*/*EOD1*), *UBIQUITION*-*SPECIFIC PROTEIASE* (*UBP15*) *GRF*-*INTERACTING FACTOR 1* (*GIF1*), *SAMBA*, *AUXIN RESPONSE FACTOR 2* (*ARF2*), *ARABIDOPSIS G PROTEIN GAMMA SUBUNIT 3* (*AGG3*), *APETALA 2* (*AP2*), *KLU* and *IKU1*/*2*^4,5^. However, the genetic and molecular mechanisms underlying seed size regulation are complicated and still largely unknown.

Cell proliferation and cell growth coordinately determine the organ size during the plant organogenesis^6,7^. GRF-INTERACTING FACTORs (GIF1/2/3), a group of transcriptional co-activators, interact with Growth-Regulating Factors (GRFs) to control leaf, flower, and root development by regulating cell proliferation and growth^8–14^. The *gif1* single mutants produce significantly smaller leaves and flowers than wild-type plants resulting from reduced cell number in Arabidopsis, while overexpression of *GIF1* results in large leaf size due to an increase of cell number^8–11,13^. In addition, GIF1 also recruits SWI/SNF chromatin remodelling complexes to its target genes that can be transcriptionally activated or repressed by GRFs^15^. In rice, overexpression of *OsGIF1* increases the size of leaves, stems, and grains, while loss-of-function of *OsGIF1* leads to small plants^16,17^. In maize, *gif1* mutants are dwarf with narrow leaves resulting from a less cell number^18^. GIFs are involved in many developmental processes, but the molecular mechanisms of transcription activation and inhibition of *GIFs* is unknown.

PEAPODs (PPD1/2), which belong to the TIFY class II protein family, control leaf development, seed growth and germination, hypocotyl elongation, stomata development and flowering time^19–22^. Suppression of *PPD* genes leads to big and dome-shape leaves resulting from prolonged cell proliferation. PPD1/2 interact with KINASE-INDUCIBLE DOMAIN INTERACTING 8/9 (KIX8/9) and TOPLESS (TPL) to form a repressor protein complex, which controls the leaf development by influencing the expression of cell division-related genes^21,22^. The stability of KIX-PPD complex is regulated by an F-box protein STERILE APETALA (SAP) that acts as a part of the SKP1/Cullin/F-box E3 ubiquitin ligase complex^22,23^. SAP positively regulates organ growth by targeting the KIX-PPD complex for 26S proteasome-dependent degradation^22,23^.

Here, we find that the KIX-PPD complex controls maternal integument development and influences seed size by regulating cell proliferation and growth. KIX8/9 and PPD1/2 interact with transcription factors MYC3/4 to form the KIX-PPD-MYC complex in Arabidopsis. The KIX-PPD-MYC complex binds to the typical G-box sequence in the *GIF1* promoter and represses its expression. Genetic analyses show that GIF1 functions as a downstream factor of the SAP-KIX-PPD-MYC signalling pathway to control seed size. Our results reveal a genetic and molecular mechanism by which the transcriptional repression of *GIF1* by the KIX-PPD-MYC complex regulates seed size in Arabidopsis.

## Results

### The KIX-PPD complex represses seed growth

**Fig. 1:**
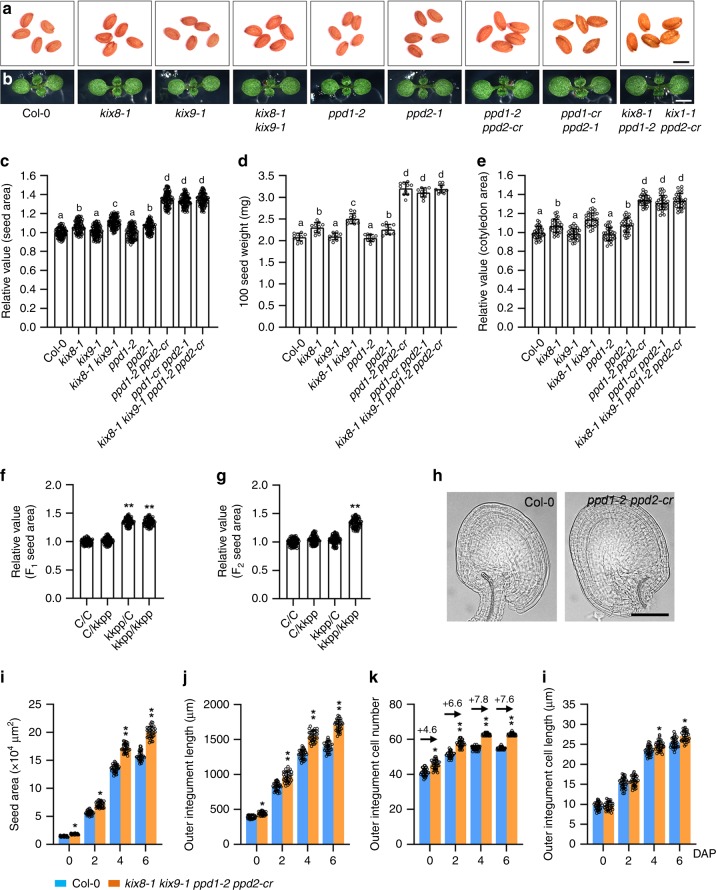
The KIX-PPD complex acts maternally to control seed development. **a**, **b** The seeds (**a**) and 8-day-old seedlings (**b**) of Col-0, *kix8*-*1*, *kix9*-*1*, *kix8*-*1 kix9*-*1*, *ppd1*-*2*, *ppd2*-*1*, *ppd1*-*2 ppd2*-*cr*, *ppd1*-*cr ppd2*-*1*, and *kix8*-*1 kix9*-*1 ppd1*-*2 ppd2*-*cr* plants. **c**–**e** The relative seed area (**c**, *n* = 100), 100 seed weight (**d**, *n* = 10), and cotyledon area (**e**, *n* = 30) of Col-0, *kix8*-*1*, *kix9*-*1*, *kix8*-*1 kix9*-*1*, *ppd1*-*2*, *ppd2*-*1*, *ppd1*-*2 ppd2*-*cr*, *ppd1*-*cr ppd2*-*1*, and *kix8*-*1 kix9*-*1 ppd1*-*2 ppd2*-*cr*. Seeds from the third to seventh silique on the stem of six plants were used for analysis. Cotyledons from the 8-day-old seedlings were used for analysis. **f**, **g** The relative area of F_1_ seeds (**f**) and F_2_ seeds (**g**) from Col-0/Col-0 (C/C), Col-0/*kix8*-*1 kix9*-*1 ppd1*-*2 ppd2*-*cr* (C/kkpp), *kix8*-*1 kix9*-*1 ppd1*-*2 ppd2*-*cr*/Col-0 (kkpp/C), and *kix8*-*1 kix9*-*1 ppd1*-*2 ppd2*-*cr*/*kix8*-*1 kix9*-*1 ppd1*-*2 ppd2*-*cr* (kkpp/kkpp) plants (*n* = 100). **h** Ovules of Col-0 and *kix8*-*1 kix9*-*1 ppd1*-*2 ppd2*-*cr* plants at 0 DAP (days after pollination). **i**–**l** The seed area (**i**), outer integument length (**j**), outer integument cell number (**k**), and outer integument cell length (**l**) of Col-0 and *kix8*-*1 kix9*-*1 ppd1*-*2 ppd2*-*cr* plants at 0, 2, 4, and 6 DAP (*n* = 33). Ovules and seeds from six siliques, which were from the fourth silique on the stem of six plants, were used for analysis. Scale bars, 0.5 mm (**a**), 0.2 cm (**b**), and 50 μm (**h**). Error bars represent ±SE. Different lowercase letters above the columns indicate the significant difference among different groups, one-way ANOVA *P*-values: *P* < 0.05. * indicates significant difference from the Col-0, one-way ANOVA *P*-values: *P < 0.05 and **P < 0.01.

KINASE-INDUCIBLE DOMAIN INTERACTING 8/9 (KIX8/9) and PEAPOD1/2 (PPD1/2) were previously reported to form a KIX-PPD complex and regulate leaf size by influencing cell proliferation in *Arabidopsis thaliana*^21,22^. Here, we found an important function of the KIX-PPD complex in seed size control. The *kix8*-*1* plants exhibited larger seeds than wild-type (Col-0) plants (Fig. 1a, c). Seed weight of *kix8*-*1* plants was also heavier than that of wild-type plants (Fig. 1d). The size of cotyledons usually reflects changes in seed size^24–26^. Consistent with this, cotyledons of *kix8*-*1* were larger than wild-type cotyledons (Fig. 1b, e). By contrast, seed size and weight and cotyledon area in *kix9*-*1* plants were similar to those in the wild type (Fig. 1a–e). The *kix8*-*1 kix9*-*1* double mutant showed significantly larger and heavier seeds and bigger cotyledons than the *kix8*-*1* and *kix9*-*1* single mutant (Fig. 1a–e), indicating that KIX8 and KIX9 function redundantly to control seed size and weight in Arabidopsis.

Seed area and weight and cotyledon area in *ppd2*-*1* plants were increased compared with those in wild-type plants, while seed area and weight and cotyledon area in *ppd1*-*2* plants were comparable to those in wild-type plants (Fig. 1a–e). Because the *PPD1* gene (*AT4G14713*) is close to the *PPD2* gene (*AT4G14720*) in the chromosome, we could not isolate the *ppd1*-*2 ppd2*-*1* double mutants. We generated *ppd2*-*cr* mutation in the *ppd1*-*2* mutant background and *ppd1*-*cr* mutation in the *ppd2*-*1* mutant background to obtain the *ppd1*-*2 ppd2*-*cr* and *ppd1*-*cr ppd2*-*1* double mutants using the CRISPR-Cas9 technology, respectively (Supplementary Fig. 1)^27^. The *ppd1*-*2 ppd2*-*cr* and *ppd1*-*cr ppd2*-*1* mutants had similar phenotypes (Fig. 1a–e). Seed area and weight and cotyledon area in *ppd1*-*2 ppd2*-*cr* and *ppd1*-*cr ppa2*-*1* mutants were significantly increased compared with those in *ppd1*-*2* and *ppd2*-*1* single mutants (Fig. 1a–e), indicating that PPD1 and PPD2 function redundantly to control seed size and weight. The *ami*-*ppd* and *ppd2*-*1* plants have been reported to produce large and curvatured leaves with more cell number^19–21^. Interestingly, we observed that *ppd1*-*2 ppd2*-*cr* and *ppd1*-*cr ppd2*-*1* showed strong curvature of leaves (Supplementary Fig. 2a), indicating that *ppd1*-*2 ppd2*-*cr* and *ppd1*-*cr ppd2*-*1* are strong alleles compared with *ami*-*ppd*. We measured the leaf area of 32-d-old Col-0, *ppd1*-*2 ppd2*-*cr* and *ppd1*-*cr ppd2*-*1* plants and found that the third to eight leaves of *ppd1*-*2 ppd2*-*cr* and *ppd1*-*cr ppd2*-*1* were smaller than those of wild type, but the tenth and eleventh leaves of *ppd1*-*2 ppd2*-*cr* and *ppd1*-*cr ppd2*-*1* were larger than those of wild type (Supplementary Fig. 2b, c). Considering that *ami*-*ppd* leaves had more cells than Col-0 leaves, we further examined palisade cell size and number of *ppd1*-*2 ppd2*-*cr* and *ppd1*-*cr ppd2*-*1* fifth leaves. The palisade cell size of *ppd1*-*2 ppd2*-*cr* and *ppd1*-*cr ppd2*-*1* plants was smaller than that of wild-type plants (Supplementary Fig. 2d, e). By contrast, the palisade cell number in *ppd1*-*2 ppd2*-*cr* and *ppd1*-*cr ppd2*-*1* leaves was higher than that in wild-type leaves (Supplementary Fig. 2e), consistent with higher cell number in *ami*-*ppd* and *ppd2*-*1* leaves^19–21^. These results supported that *ppd1*-*2 ppd2*-*cr* and *ppd1*-*cr ppd2*-*1* promote cell proliferation in leaves, but decrease cell expansion. These data also suggest a possible compensation mechanism between cell number and cell size in *ppd1*-*2 ppd2*-*cr* and *ppd1*-*cr ppd2*-*1*. This compensation phenomenon has been observed in several mutants^28–30^.

Considering that KIX8/9 and PPD1/2 function in a complex^21,22^, we investigated the effect of simultaneous disruption of KIX8/9 and PPD1/2 on seed size. Seed area and weight and cotyledon area in the *kix8*-*1 kix9*-*1 ppd1*-*2 ppd2*-*cr* quadruple mutant were similar to those of *ppd1*-*2 ppd2*-*cr* or *ppd1*-*cr ppa2*-*1* double mutants (Fig. 1a–e) suggesting that KIX8/9 and PPD1/2 act in the common pathway to control seed size. Together, these results show that the KIX-PPD complex restricts seed growth in Arabidopsis.

### The KIX-PPD complex acts maternally to regulate seed size

Seed size is determined coordinately by the growth of maternal and zygotic tissues^1,4^. To investigate whether the KIX-PPD complex functions maternally or zygotically to control seed size, we performed the reciprocal crossing experiments between the wild type and *kix8*-*1 kix9*-*1 ppd1*-*2 ppd2*-*cr*. As shown in Fig. 1f, the F_1_ seed area of Col-0 plants pollinated with the pollen of *kix8*-*1 kix9*-*1 ppd1*-*2 ppd2*-*cr* plants was similar to that of self-pollinated Col-0 plants, and the F_1_ seed area of *kix8*-*1 kix9*-*1 ppd1*-*2 ppd2*-*cr* plants pollinated with the pollen of Col-0 plants was comparable to that of self-pollinated *kix8*-*1 kix9*-*1 ppd1*-*2 ppd2*-*cr* plants. The size of Col-0/*kix8*-*1 kix9*-*1 ppd1*-*2 ppd2*-*cr* and *kix8*-*1 kix9*-*1 ppd1*-*2 ppd2*-*cr*/Col-0 F_2_ seeds was similar to that of Col-0/Col-0 F_2_ seeds and smaller than that of *kix8*-*1 kix9*-*1 ppd1*-*2 ppd2*-*cr*/*kix8*-*1 kix9*-*1 ppd1*-*2 ppd2*-*cr* F_2_ seeds (Fig. 1g). In addition, reciprocal crossing experiments showed that *kix8*-*1* or *ppd2*-*1* single mutation acts maternally to influence seed size (Supplementary Fig. 3). Together, these results indicate that the KIX-PPD complex regulates seed growth through the maternal tissue of mother plants.

As the integuments belong to maternal tissues in Arabidopsis, we investigated the development of the outer integuments in the wild type and *kix8*-*1 kix9*-*1 ppd1*-*2 ppd2*-*cr*. The *kix8*-*1 kix9*-*1 ppd1*-*2 ppd2*-*cr* plants had bigger ovule area and longer outer integuments than wild-type plants at 0 DAP (days after pollination) (Fig. 1h–j). We then counted the number of cells in wild-type and *kix8*-*1 kix9*-*1 ppd1*-*2 ppd2*-*cr* outer integuments and found that *kix8*-*1 kix9*-*1 ppd1*-*2 ppd2*-*cr* outer integuments contained more cells than wild-type outer integuments (Fig. 1k). By contrast, the length of cells in *kix8*-*1 kix9*-*1 ppd1*-*2 ppd2*-*cr* outer integuments was similar to that in wild-type outer integuments (Fig. 1l). These results indicate that the KIX-PPD module restricts cell proliferation in outer integuments before fertilisation. We then examined cell number and cell length in wild-type and *kix8*-*1 kix9*-*1 ppd1*-*2 ppd2*-*cr* outer integuments at 2, 4 and 6 DAP, respectively. The outer integument cell number and cell length of *kix8*-*1 kix9*-*1 ppd1*-*2 ppd2*-*cr* were increased compared with those of the wild type at 4 and 6 DAP (Fig. 1k–l), thereby resulting in long outer integument and large seed size in the *kix8*-*1 kix9*-*1 ppd1*-*2 ppd2*-*cr* plants (Fig. 1i–j). These results indicate that the KIX-PPD module limits both cell proliferation and cell elongation in outer integuments after fertilisation. We further compared the effect of *kix8*-*1 kix9*-*1 ppd1*-*2 ppd2*-*cr* on cell proliferation during ovule and seed developmental processes. As shown in Fig. 1k, the KIX-PPD module restrains cell proliferation of the outer integument during both ovule and early seed developmental stages.

### The formation of the KIX8/9-PPD1/2-MYC3/4 complex

**Fig. 2:**
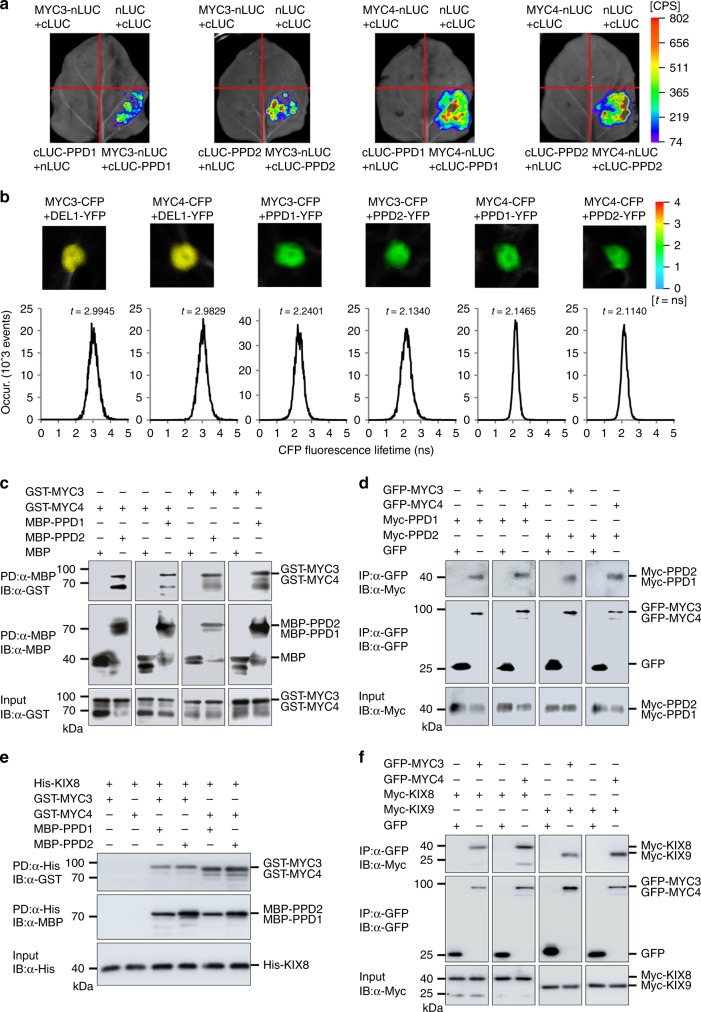
KIX8/9, PPD1/2, and MYC3/4 form a complex in Arabidopsis. **a** Split luciferase complementation assays showing the interactions between PPD1/2 and MYC3/4. MYC3/4-nLUC and cLUC-PPD1/2 were coexpressed in *N. benthamiana* leaves. The luciferase activity was detected at 2 days later after infiltration. **b** The FRET-FLIM assays showing that MYC3/4 interact with PPD1/2 in *N. benthamiana* leaves. CFP fluorescence lifetime was obtained at 2 days later after coinfiltrating with different combinations of *35S*:*MYC3*-*CFP*, *35S*:*MYC4*-*CFP*, *35S*:*PPD1*-*YFP*, *35S*:*PPD2*-*YFP*, and *35S*:*DEL1*-*YFP* constructs. *35S*:*DEL1*-*YFP* was used as a negative control. **c** Pull-down analyses showing the interactions between PPD1/2 and MYC3/4 in vitro. GST-MYC3 and GST-MYC4 were incubated with MBP-PPD1, MBP-PPD2, and MBP, respectively. Proteins were pulled down by MBP-Trap-A agarose beads and detected by Western blot with anti-GST or anti-MBP antibody. **d** Co-immunoprecipitation analyses showing the interactions between PPD1/2 and MYC3/4 in Arabidopsis. Total protein extracts of *35S*:*Myc*-*PPD1*/*2*;*35S*:*GFP* and *35S*:*Myc*-*PPD1*/*2*;*35S*:*GFP*-*MYC3*/*4* plants were incubated with GFP-Trap agarose beads. Precipitates were detected by Western blot with anti-GFP or anti-Myc antibody. **e** Pull-down analyses showing that PPD1/2 are required for the interactions between KIX8 and MYC3/4. His-KIX8 was incubated with GST-MYC3 or GST-MYC4 and MBP-PPD1 or MBP-PPD2. Proteins were pulled down by the Ni-NTA agarose beads and detected by Western blot with anti-GST, anti-MBP, or anti-His antibody. **f** Co-immunoprecipitation analyses showing that KIX8/9 and MYC3/4 are in a protein complex in Arabidopsis. Total protein extracts of *35S*:*Myc*-*KIX8*/*9;35S*:*GFP* and *35S*:*Myc*-*KIX8*/*9;35S*:*GFP*-*MYC3*/*4* plants were incubated with GFP-Trap agarose beads. Precipitates were detected by Western blot with anti-GFP or anti-Myc antibody.

PPD1/2 and 12 JAZ proteins belong to TIFY class II protein family^31^. JAZ proteins usually interact with transcription factors to perform their functions, such as ENHANCER OF GLABRA 3 (EGL3), GLABROUS 3 (GL3), TRANSPARENT TESTA 8 (TT8), MYELOCYTOMATOSIS 2/3/4 (MYC2/3/4), MYB21/24, GLABRA 1 (GL1), PURPLE ACID PHOSPHATASE 1 (PAP1), ETHYLENE-INSENSITIVE 3 (EIN3) and ETHYLENE-INSENSITIVE3-LIKE 1 (EIL1)^32–35^. We therefore used the split luciferase complementation assays to test whether PPD proteins could interact with these transcription factors. We found that PPD1 and PPD2 interacted with MYC3 and MYC4 (Fig. 2a), but not with MYC2 and other transcription factors (Supplementary Fig. 4). By contrast, we did not detect the interactions between KIX8/9 and MYC2/3/4 in split luciferase complementation assays (Supplementary Fig. 5). The interactions between PPD1/2 and MYC3/4 were further verified by forster resonance energy transfer and fluorescence lifetime imaging microscopy analyses (FRET-FLIM). As shown in Fig. 2b, the CFP fluorescence lifetime of MYC3-CFP was significantly decreased by the PPD1-YFP or PPD2-YFP in *N. benthamiana* leaves. The CFP fluorescence lifetime of MYC4-CFP was also significantly decreased by the PPD1-YFP or PPD2-YFP in *N. benthamiana* leaves. The bimolecular fluorescence complementation assays also showed that nYFP-PPD1 and nYFP-PPD2 associated with cYFP-MYC3 and cYFP-MYC4, but not with the cYFP control (Supplementary Fig. 6). To determine whether PPD1/2 could directly interact with MYC3/4 in vitro, we performed pull-down analyses. As shown in Fig. 2c, GST-MYC3/4 bound to MBP-PPD1/2 in vitro, but not the MBP control.

To investigate whether PPD1/2 could interact with MYC2/3/4 in Arabidopsis, we generated *35S*:*Myc*-*PPD1* #8, *35S*:*Myc*-*PPD2* #5, *35S*:*GFP*-*MYC2* #4, *35S*:*GFP*-*MYC3* #12, and *35S*:*GFP*-*MYC4* #8 transgenic lines. *35S*:*Myc*-*PPD1* #8, *35S*:*Myc*-*PPD2* #5, *35S*:*GFP*-*MYC3* #12, and *35S*:*GFP*-*MYC4* #8 transgenic lines produced small seeds (Supplementary Figs. 9 and 10), indicating that they are functional. We crossed *35S*:*Myc*-*PPD1* #8 and *35S*:*Myc*-*PPD2* #5 lines with *35S*:*GFP*, *35S*:*GFP*-*MYC2* #4, *35S*:*GFP*-*MYC3* #12, and *35S*:*GFP*-*MYC4* #8 lines to generate *35S*:*Myc*-*PPD1*;*35S*:*GFP*, *35S*:*Myc*-*PPD1*;*35S*:*GFP*-*MYC2*, *35S*:*Myc*-*PPD1*;*35S*:*GFP*-*MYC3*, *35S*:*Myc*-*PPD1*;*35S*:*GFP*-*MYC4*, *35S*:*Myc*-*PPD2*;*35S*:*GFP*, *35S*:*Myc*-*PPD2*;*35S*:*GFP*-*MYC2*, *35S*:*Myc*-*PPD2*;*35S*:*GFP*-*MYC3* and *35S*:*Myc*-*PPD2*;*35S*:*GFP*-*MYC4* plants, respectively. Co-immunoprecipitation analyses (co-IP) showed that Myc-PPD1/2 associated with GFP-MYC3/4 but not with the GFP control (Fig. 2d), indicating that PPD1/2 and MYC3/4 form a complex in Arabidopsis. The interactions between Myc-PPD1/2 and GFP-MYC2 were not found by co-immunoprecipitation analyses in Arabidopsis (Supplementary Fig. 7).

Pull-down analyses showed that His-KIX8 could not directly associate with GST-MYC3/4 in vitro (Fig. 2e). Considering that KIX8/9 can interact with PPD1/2^21^, we incubated His-KIX8 with MBP-PPD1/2 and GST-MYC3/4. When proteins were pulled down by Ni-NTA agarose, we detected the MBP-PPD1/2 and GST-MYC3/4 proteins (Fig. 2e), indicating that KIX8, PPD1/2, and MYC3/4 could form a complex in vitro. The associations of His-KIX9 with GST-MYC3/4 were not found in similar assays (Supplementary Fig. 8). To further investigate whether KIX8/9, PPD1/2, and MYC3/4 form a complex in Arabidopsis, we generated *35S*:*Myc*-*KIX8* #6 and *35S*:*Myc*-*KIX9* #14 transgenic lines that formed small seeds compared with the wild type (Supplementary Fig. 9), and crossed them with *35S*:*GFP*, *35S*:*GFP*-*MYC3* #12, and *35S*:*GFP*-*MYC4* #8 plants to isolate *35S*:*Myc*-*KIX8*;*35S*:*GFP*, *35S*:*Myc*-*KIX8*;*35S*:*GFP*-*MYC3*, *35S*:*Myc*-*KIX8*;*35S*:*GFP*-*MYC4*, *35S*:*Myc*-*KIX9*;*35S*:*GFP*, *35S*:*Myc*-*KIX9*;*35S*:*GFP*-*MYC3* and *35S*:*Myc*-*KIX9*;*35S*:*GFP*-*MYC4* plants, respectively. Co-immunoprecipitation analyses revealed that Myc-KIX8/9 and GFP-MYC3/4 exist in a complex (Fig. 2f). Together, these results indicate that KIX8/9, PPD1/2, and MYC3/4 form a KIX-PPD-MYC complex in Arabidopsis.

### MYC3 and MYC4 function redundantly to regulate seed size

**Fig. 3:**
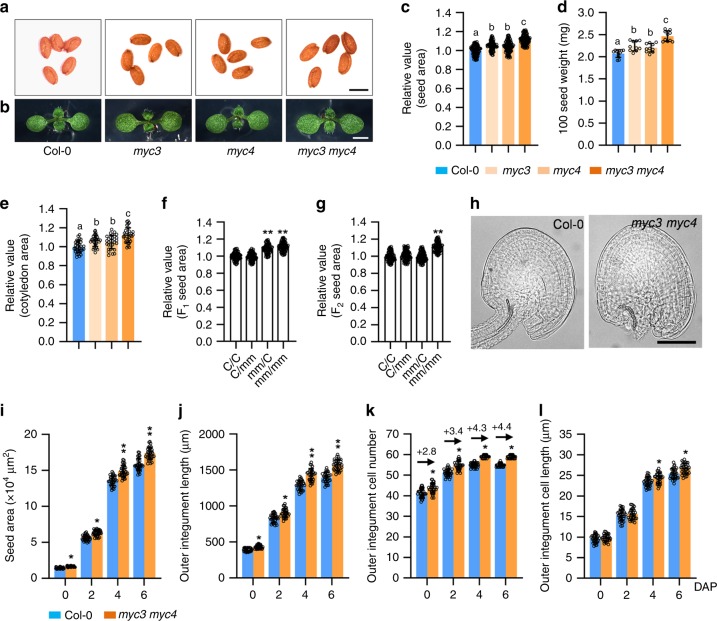
*MYC3* and *MYC4* act maternally to control seed development. **a**, **b** The seeds (**a**) and 8-day-old seedlings (**b**) of Col-0, *myc3*, *myc4*, and *myc3 myc4*. **c**–**e** The relative seed area (**c**, *n* = 100), 100 seed weight (**d**, *n* = 10), and cotyledon area (**e**, *n* = 30) of Col-0, *myc3*, *myc4* and *myc3 myc4*. Seeds from the third to seventh silique on the stem of six plants were used for analysis. Cotyledons from the 8-day-old seedlings were used for analysis. **f**, **g** The relative area of F_1_ seeds (**f**) and F_2_ seeds (**g**) from the Col-0/Col-0 (C/C), Col-0/*myc3 myc4* (C/mm), *myc3 myc4*/Col-0 (mm/C), and *myc3 myc4*/*myc3 myc4* (mm/mm) plants (n = 100). **h** Ovules of Col-0 and *myc3 myc4* plants at 0 DAP (days after pollination). **i**–**l** The seed area (**i**), outer integument length (**j**), outer integument cell number (**k**), and outer integument cell length (**l**) of Col-0 and *myc3 myc4* plants at 0, 2, 4, and 6 DAP (n = 33). Ovules and seeds from six siliques, which were from the fourth silique on the stem of six plants, were used for analysis. Scale bars, 0.5 mm (**a**), 0.2 cm (**b**), and 50 μm (**h**). Error bars represent ±SE. Different lowercase letters above the columns indicate the significant difference among different groups, one-way ANOVA *P*-values: *P* < 0.05. * indicates significant difference from the Col-0, one-way ANOVA *P*-values: *P < 0.05 and **P < 0.01.

As MYC3/4 could interact with PPD1/2, we asked whether *MYC3*/*4* influence seed size. As shown in Fig. 3a–c, e, *myc3* (GK_445B11) and *myc4* (GK_491E10) plants produced larger seeds and cotyledons than the wild-type plants, consistent with a previous study^36^. The *myc3* and *myc4* plants also had heavier seed weight than the wild-type plants (Fig. 3d). The *myc3 myc4* double mutant produced larger and heavier and bigger cotyledons than *myc3* and *myc4* single mutant (Fig. 3a–e), indicating that *MYC3* and *MYC4* function redundantly to regulate seed size and weight. In addition, overexpression of GFP-MYC3/4 fusion proteins driven by the CaMV 35S promoter in wild-type plants resulted in small and light seeds compared with the wild type (Supplementary Fig. 10), indicating that MYC3/4 limit seed growth in Arabidopsis.

To investigate whether *MYC3*/*4* function maternally or zygotically to control seed size, we performed reciprocal crossing experiments between the *myc3 myc4* and wild type plants. As shown in Fig. 3f, the F_1_ seed area of Col-0 plants pollinated with the pollen of *myc3 myc4* plants was similar to that of self-pollinated Col-0 plants, and the F_1_ seed area of *myc3 myc4* plants pollinated with the pollen of Col-0 plants was comparable to that of self-pollinated *myc3 myc4* plants. In addition, the size of Col-0/*myc3 myc4* and *myc3 myc4*/Col-0 F_2_ seeds was similar to that of Col-0/Col-0 F_2_ seeds and smaller than that of *myc3 myc4*/*myc3 myc4* F_2_ seeds (Fig. 3g). These results indicate that *MYC3*/*4* act maternally to control seed size.

We then examined the development of wild-type and *myc3 myc4* outer integuments. *myc3 myc4* plants had larger ovules and longer outer integuments than the wild-type plants at 0 DAP (Fig. 3h–j). The number of cells in *myc3 myc4* outer integuments was increased compared with that in wild-type outer integuments, while the outer integument cell length of *myc3 myc4* was similar to that of the wild type at 0 DAP (Fig. 3k, l). These results indicate that *MYC3*/*4* limit cell proliferation in the ovules before fertilisation. We further examined cell number and cell length in wild-type and *myc3 myc4* outer integuments at 2, 4 and 6 DAP. The outer integument cell number and length of *myc3 myc4* were significantly increased compared with those of the wild type at 4 and 6 DAP, thereby resulting in longer outer integument and larger seed size in the *myc3 myc4* plants (Fig. 3i–l). These results indicate that *MYC3*/*4* limit both cell proliferation and cell elongation in outer integuments after fertilisation, consistent with the role of the KIX-PPD complex. We further compared the effect of *myc3 myc4* on cell proliferation during ovule and seed developmental processes. As shown in Fig. 3k, *MYC3*/*4* restrict cell proliferation in the integuments during both ovule and early seed developmental stages.

### The KIX-PPD-MYC complex represses *GIF1* expression

**Fig. 4:**
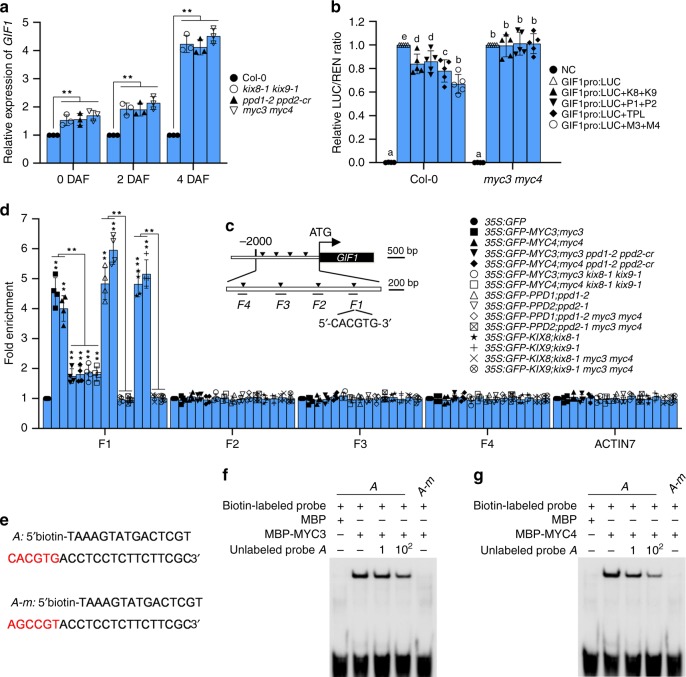
The KIX-PPD-MYC complex associates with the promoter of *GIF1* and represses its expression. **a** The relative expression levels of *GIF1* in the 0, 2 and 4 DAF (days after flowering) siliques of Col-0, *kix8*-*1 kix9*-*1*, *ppd1*-*2 ppd2*-*cr* and *myc3 myc4* were detected by qPCR (*n* = 3). Data was normalised with *ACTIN2*. **b** The LUC activity of *GIF1pro*:*LUC* from the transient expression analysis in the Col-0 and *myc3 myc4* protoplast (*n* = 5). *GIF1pro*:*LUC* was cotransfected with different combinations of *35S*:*Myc*-*KIX8* (K8), *35S*:*Myc*-*KIX9* (K9), *35S*:*Myc*-*PPD1* (P1), *35S*:*Myc*-*PPD2* (P2), *35S*:*Myc*-*MYC3* (M3), *35* *S*:*Myc*-*MYC4* (M4), and *35S*:*Myc*-*TPL* (TPL) into the Col-0 and *myc3 myc4* protoplast. The LUC and REN luciferase activities of *GIF1pro*:*LUC* were measured 40 h later after transfection. NC (*pGreen II_0800*-*LUC*) was used as a negative control. **c** The schematic diagram of *GIF1* promoter containing a typical G-box (5′-CACGTG-3′) sequence in *F1* fragment. *F1*-*F4* represent DNA fragments used for ChIP-qPCR analysis. **d** ChIP-qPCR assays showing that KIX8/9 and PPD1/2 associate with the promoter of *GIF1* by MYC3/4 in Arabidopsis (*n* = 4). Chromatin from 1 to 4 DAF siliques of *35S*:*GFP*, *35S*:*GFP*-*MYC3*;*myc3*, *35S*:*GFP*-*MYC4*;*myc4*, *35S:GFP-MYC3;myc3* *ppd1*-*2 ppd2*-*cr*, *35S*:*GFP*-*MYC4*;*myc4 ppd1*-*2 ppd2*-*cr*, *35S*:*GFP*-*MYC3*;*myc3 kix8*-*1* *kix9-1*, *35S*:*GFP*-*MYC4*;*myc4 kix8*-*1* *kix9-1*, *35S*:*GFP*-*PPD1*;*ppd1*-*2*, *35S*:*GFP*-*PPD2*;*ppd2*-*1*, *35S*:*GFP*-*PPD1*;*ppd1*-*2 myc3 myc4*, *35S*:*GFP*-*PPD2*;*ppd2*-*1 myc3 myc4*, *35S*:*GFP*-*KIX8*;*kix8*-*1*, *35S*:*GFP*-*KIX9*;*kix9*-*1*, *35S*:*GFP*-*KIX8*;*kix8*-*1 myc3 myc4* and *35S*:*GFP*-*KIX9*;*kix9*-*1 myc3 myc4* were incubated with ChIP anti-GFP antibody and precipitated by ChIP protein A + G magnetic beads. The enrichment of fragments was determined by qPCR. The *35S*:*GFP* plants acted as a control. The *ACTIN7* promoter was used as a negative control. **e** The sequence of *A* and *A*-*m* probes for EMSA analysis. **f**, **g** The associations of MBP-MYC3 (**g**) and MBP-MYC4 (**i**) with the promoter of *GIF1* were detected by EMSA. 5′-biotin-*A*/*A*-*m* probes were incubated with MBP or MBP-MYC3/4 and detected by ChIP western blot with the anti-biotin antibody. Error bars represent ±SE. Asterisk indicates significant difference, one-way ANOVA *P*-values: **P < 0.01. Different lowercase letters above the columns indicate the significant difference among different groups, one-way ANOVA *P*-values: *P* < 0.05.

We previously reported the SAP-KIX-PPD signalling pathway has an important role in leaf size control^22,23^, and performed the RNA-seq analysis using the first pair of leaves of 9-day-old *myc3 myc4* and *ppd1*-*2 ppd2*-*cr* seedlings. 149 genes with significantly changed expression were found in both *myc3 myc4* and *ppd1*-*2 ppd2*-*cr* plants (Supplementary Data 1). One of them was the transcriptional coactivator *GIF1* (*GRF*-*INTERACTING FACTOR 1*), which has been reported to control the size of leaves, flowers, seeds, and cotyledons^8–11,13,37,38^. The expression of *GIF1* was significantly upregulated in both *myc3 myc4* and *ppd1*-*2 ppd2*-*cr* seedlings (Supplementary Data 1). We also found that expression levels of *GIF1* were significantly higher in *kix8*-*1 kix9*-*1*, *ppd1*-*2 ppd2*-*cr*, and *myc3 myc4* siliques than those in wild-type siliques at 0, 2, and 4 DAF (days after flowering) (Fig. 4a). By contrast, expression levels of *GIF1* were decreased in the 2 DAF siliques of *35S*:*Myc*-*KIX*8 #6, *35S*:*Myc*-*KIX*9 #14, *35S*:*Myc*-*PPD1* #8, *35S*:*Myc*-*PPD2* #5, *35S*:*GFP*-*MYC3* #12, and *35S*:*GFP*-*MYC4* #8 plants compared with those of wild-type plants (Supplementary Fig. 11). In addition, the LUC activity of *GIF1pro*:*LUC* was significantly reduced by overexpressing *Myc*-*KIX8*/*9*, *Myc*-*PPD1*/*2* and *Myc*-*MYC3*/*4* in the Col-0 protoplast (Fig. 4b). These results indicate that the KIX-PPD-MYC complex represses *GIF1* expression. The KIX-PPD module limits leaf development by the repressor TOPLESS (TPL)^21,22^. Overexpression of Myc-TPL also reduced the LUC activity of *GIF1pro*:*LUC* in the Col-0 protoplast (Fig. 4b). Plant cis-acting regulatory DNA element analysis showed that there was a typical G-box sequence (5′-CACGTG-3′) at the -425 bp site in the 2 kb promoter region of *GIF1* (https://www.dna.affrc.go.jp/PLACE/?action=newplace) (Fig. 4c). MYCs and PPDs had been reported to associate with the G-box sequence to regulate target gene expression^21,39^. Furthermore, down-regulation of *PPDs* orthologs in legume *Medicago truncatula* and legume soybean leads to significant increases in expression of *MtGIF1* and *GmGIF1*^40^. These results imply that *GIF1* might be a target gene of the KIX-PPD-MYC repressive complex.

To test this possibility, we performed the chromatin immunoprecipitation-quantitative PCR (ChIP-qPCR) assays using the 1–4 DAF siliques of *35S*:*GFP*, *35S*:*GFP*-*MYC3*;*myc3*, and *35S*:*GFP*-*MYC4*;*myc4* plants. The *35S*:*GFP*-*MYC3*;*myc3* and *35S*:*GFP*-*MYC4*;*myc4* plants formed small seeds compared with the wild type, indicating GFP-MYC3 and GFP-MYC4 are functional (Supplementary Fig. 12). As shown in Fig. 4c, d, the fragment *F1* from the *GIF1* promoter containing the typical G-box cis-acting element in *35S*:*GFP*-*MYC3*;*myc3* and *35S*:*GFP*-*MYC4*;*myc4* plants was remarkably enriched compared with that in *35S*:*GFP* plants. The fragment *F1* from the *GIF1* promoter was also significantly enriched compared with other fragments (*F2*-*F4*) without the typical G-box element and the negative control (a fragment of *ACTIN7* promoter) in *35S*:*GFP*-*MYC3*;*myc3* and *35S*:*GFP*-*MYC4*;*myc4* plants, indicating that MYC3 and MYC4 associate with the *GIF1* promoter through the fragment *F1* in Arabidopsis. We then asked whether KIX8/9 and PPD1/2 could influence the associations of MYC3 and MYC4 with the *GIF1* promoter. In *35S*:*GFP*-*MYC3*;*myc3 kix8*-*1 kix9*-*1*, *35S*:*GFP*-*MYC4*;*myc4 kix8*-*1 kix9*-*1*, *35S*:*GFP*-*MYC3*;*myc3 ppd1*-*2 ppd2*-*cr* and *35S*:*GFP*-*MYC4*;*myc4 ppd1*-*2 ppd2*-*cr* plants, the enrichment of *F1* fragments was substantially decreased compared with that in *35S*:*GFP*-*MYC3*;*myc3* and *35S*:*GFP*-*MYC4*;*myc4* plants (Fig. 4d), indicating that KIX8/9 and PPD1/2 are required for MYC3/4 to effectively bind to the *GIF1* promoter. The fragment *F1* from the *GIF1* promoter in *35S*:*GFP*-*KIX8*;*kix8*-*1*, *35S*:*GFP*-*KIX9*;*kix9*-*1*, *35S*:*GFP*-*PPD1*;*ppd1*-*2*, and *35S*:*GFP*-*PPD2*;*ppd2*-*1* plants also could be strongly enriched compared with that in the *35S*:*GFP* plants, while it could not be enriched in *35S*:*GFP*-*KIX8*;*kix8*-*1 myc3 myc4*, *35S*:*GFP*-*KIX9*;*kix9*-*1 myc3 myc4*, *35S*:*GFP*-*PPD1*;*ppd1*-*2 myc3 myc4*, and *35S*:*GFP*-*PPD2*;*ppd2*-*1 myc3 myc4* plants (Fig. 4d). These results indicate that MYC3/4 are required for KIX8/9 and PPD1/2 to bind to the *GIF1* promoter and repress its expression. Consistent with this, the LUC activity of *GIF1pro*:*LUC* was not reduced by the overexpression of Myc-KIX8/9, Myc-PPD1/2, and Myc-TPL driven by the CaMV 35S promoter in the *myc3 myc4* protoplast (Fig. 4b).

To investigate whether MYC3/4 could directly bind to the G-box cis-acting element in the promoter of *GIF1*, we performed the electrophoretic mobility shift assays (EMSA). As shown in Fig. 4e–g, MBP-MYC3 and MBP-MYC4 bound to the biotin-labelled probe *A* from the *GIF1* promoter containing the typical G-box (5′-CACGTG-3′) but not to the mutated biotin-labelled probe *A* (*A*-*m*). The binding ability of MBP-MYC3 and MBP-MYC4 to the probe *A* was decreased by adding the biotin-unlabelled probe *A*. These results indicate that MYC3 and MYC4 directly bind to the *GIF1* promoter.

### GIF1 acts maternally to control seed size

**Fig. 5:**
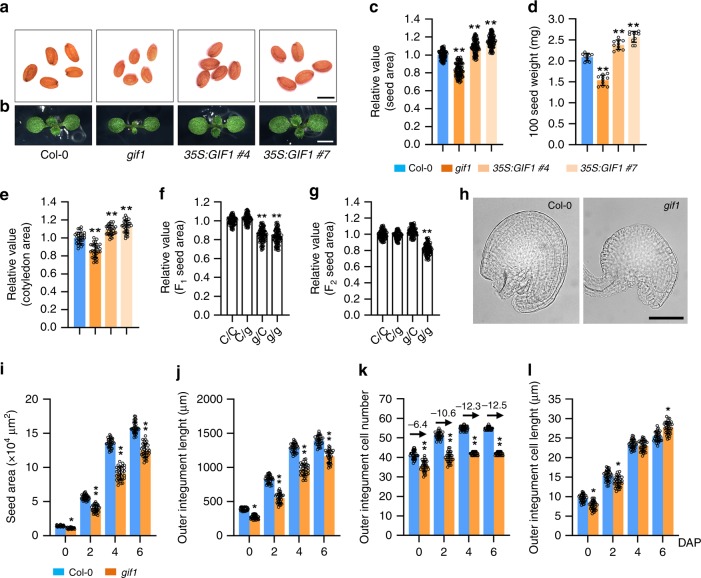
*GIF1* acts maternally to control seed development. **a**, **b** The seeds (**a**) and 8-day-old seedlings (**b**) of Col-0, *gif1*, *35S*:*GIF1 #*4, and *35* *S*:*GIF1 #*7. **c**–**e** The relative seed area (**c**, *n* = 100), 100 seed weight (**d**, *n* = 10), and cotyledon area (**e**, *n* = 30) of Col-0, *gif1*, *35S*:*GIF1 #*4, and *35S*:*GIF1 #*7. Seeds from the third to seventh silique on the stem of six plants were used for analysis. Cotyledons from the 8-day-old seedlings were used for analysis. **f**, **g** The relative area of F_1_ seeds (**f**) and F_2_ seeds (**g**) from Col-0/Col-0 (C/C), Col-0/*gif1* (C/g), *gif1*/Col-0 (g/C), and *gif1*/*gif1*(g/g) plants (*n* = 100). **h** The Mature ovule of Col-0 and *gif1* plants at 0 DAP (days after pollination). **i**–**l** The seed area (**i**), outer integument length (**j**), outer integument cell number (**k**), and outer integument cell length (**l**) of Col-0 and *gif1* plants at 0, 2, 4, and 6 DAP (n = 33). Ovules and seeds from six siliques, which were from the fourth silique on the stem of six plants, were used for analysis. Scale bars, 0.5 mm (**a**), 0.2 cm (**b**), and 50 μm (**h**). Error bars represent ±SE. Different lowercase letters above the columns indicate the significant difference among different groups, one-way ANOVA *P*-values: *P* < 0.05. Asterisk indicates significant difference from the Col-0, one-way ANOVA *P*-values: *P < 0.05 and **P < 0.01.

The *gif1* plants (SALK_150407) produced smaller leaves, seeds, and cotyledons than wild-type plants (Fig. 5a–c, e, and Supplementary Fig. 13a), consistent with previous studies^8,10,11^. The seed weight of *gif1* plants was also significantly lower than that of wild-type plants (Fig. 5d). In addition, the fertility of *gif1* plants was lower than that of wild-type plants (Supplementary Fig. 13b). By contrast, overexpression of *GIF1* (*35S*:*GIF1*) led to bigger and heavier seeds and bigger cotyledons than the wild type (Fig. 5a–e). These results indicate that *GIF1* is required for normal seed and other organs development.

To investigate whether *GIF1* functions maternally or zygotically to control seed size, we conducted reciprocal crossing experiments between the wild type and *gif1*. As shown in Fig. 5f, the F_1_ seed size of Col-0 plants pollinated with the pollen of *gif1* plants was similar to that of self-pollinated Col-0 plants, and the F_1_ seed size of *gif1* plants pollinated with the pollen of Col-0 plants was comparable to that of self-pollinated *gif1* plants. In addition, the size of Col-0/*gif1* and *gif1*/Col-0 F_2_ seeds was similar to that of Col-0/Col-0 F_2_ seeds and bigger than that of *gif1*/*gif1* F_2_ seeds (Fig. 5g). These results support that *GIF1* acts maternally to control seed size.

We then investigated the outer integument cell number and cell length before and after fertilisation. The *gif1* plants had shorter outer integuments with fewer and shorter cells than the wild type at 0 DAP (Fig. 5h, j–l). We further examined the outer integument cell number and cell length after fertilisation. The *gif1* showed shorter outer integuments and smaller seeds than the wild type before 6 DAP (Fig. 5i, j). The outer integument cell number of *gif1* was significantly decreased compared with that of the wild type at 2, 4 and 6 DAP (Fig. 5k). The cells in *gif1* outer integuments were shorter than those in wild-type outer integuments at 2 DAP, while they were longer than those in wild-type outer integuments at 6 DAP (Fig. 5l), suggesting a compensation phenomenon between cell proliferation and cell elongation^28,41,42^. In addition, we observed that *gif1* decreases cell proliferation in outer integuments during both ovule and early seed developmental stages (Fig. 5k).

### Partially overlapping expression of KIX-PPD-MYC

**Fig. 6:**
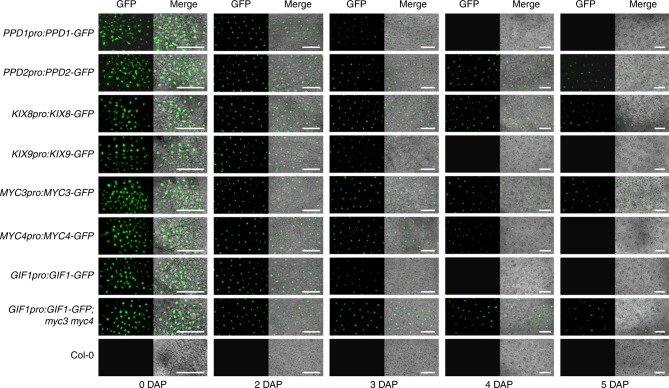
Expression patterns of *KIX8*, *KIX9*, *PPD1*, *PPD2*, *MYC3*, *MYC4*, and *GIF1* in epidermal cells of the outer integuments during seed development. The promotors of *KIX8* (2,087 bp), *KIX9* (1,714 bp), *PPD1* (1,797 bp), *PPD2* (2,153 bp), *MYC3* (2,180 bp), *MYC4* (2,132 bp), and *GIF1* (2,337 bp) and their CDSs were cloned into the *pMDC107*-*GFP* vector to generate *KIX8pro*:*KIX8*-*GFP*, *KIX9pro*:*KIX9*-*GFP*, *PPD1pro*:*PPD1*-*GFP, PPD2pro*:*PPD2*-*GFP*, *MYC3pro*:*MYC3*-*GFP*, *MYC4pro*:*MYC4*-*GFP*, and *GIF1pro*:*GIF1*-*GFP* constructs, respectively. GFP fluorescence from the outer integuments of seeds was observed during seed development. DAP days after pollination. Scale bars = 25 μm.

As *KIX8*, *KIX9*, *PPD1*, *PPD2*, *MYC3*, *MYC4* and *GIF1* function in a signalling pathway to regulate seed size, we asked whether they have the similar expression patterns during ovule and seed development. To test this, we generated the *KIX8pro*:*KIX8*-*GFP*, *KIX9pro*:*KIX9*-*GFP*, *PPD1pro*:*PPD1*-*GFP*, *PPD2pro*:*PPD2*-*GFP*, *MYC3pro*:*MYC3*-*GFP*, *MYC4pro*:*MYC4*-*GFP*, and *GIF1pro*:*GIF1*-*GFP* transgenic lines. As shown in Fig. 6 and Supplementary Fig. 14, KIX8, KIX9, PPD1, PPD2, MYC3, MYC4, and GIF1 expressed in the integument and chalazal region of ovules before fertilisation. As shown in Fig. 6, PPD1, KIX9 and GIF1 strongly expressed in the nuclei of outer integument cells before 2 DAP and became weak from 3 DAP during seed development. MYC4 strongly expressed in the nuclei of outer integument cells before 3 DAP and became weak from 4 DAP during seed development. PPD2, KIX8, and MYC3 expressed in the nuclei of outer integument cells before 6 DAP. As shown in Supplementary Fig. 14, PPD1, KIX9 and GIF1 also expressed in the chalazal domain cells of seeds before 2 DAP and were not observed at 4 DAP. KIX8, PPD2, MYC3, and MYC4 expressed in the chalazal domain cells of seeds before 4 DAP. In addition, MYC3 and GIF1 expressed in endosperms before 4 DAP. These results indicate that *KIX8*, *KIX9*, *PPD1*, *PPD2*, *MYC3*, *MYC4* and *GIF1* have overlapped expression patterns during ovule development and possess partially overlapped expression patterns during seed development, supporting that they function in a common pathway to control seed size. Moreover, the GFP fluorescence in the epidermal cells of *GIF1pro*:*GIF1*-*GFP*;*myc3 myc4* outer integuments was observed at 4 and 5 DAP, which was not observed in *GIF1pro*:*GIF1*-*GFP* plants (Fig. 6), supporting that MYC3/4 repress *GIF1* expression.

### GIF1 acts with the KIX-PPD-MYC module to control seed size

**Fig. 7:**
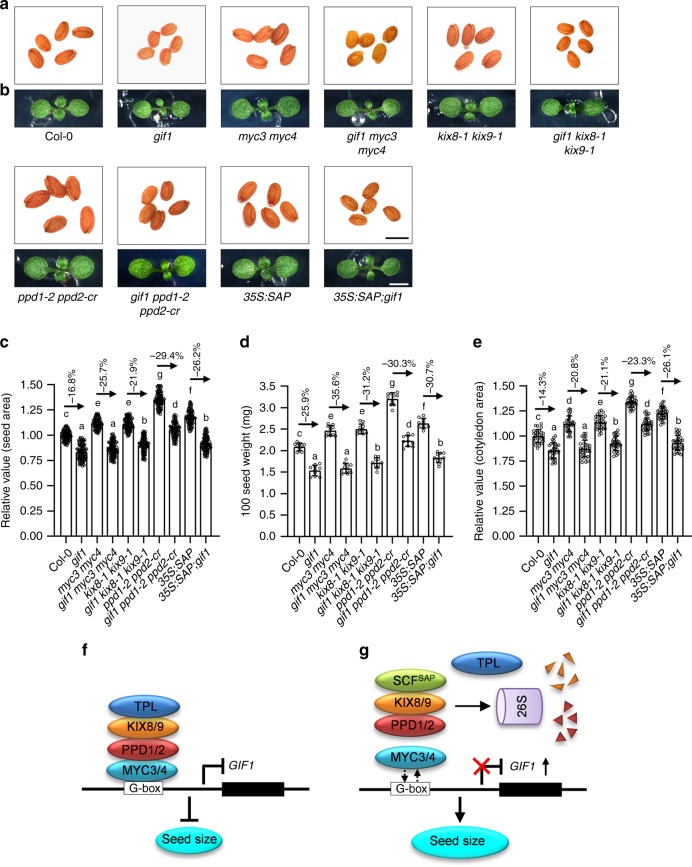
GIF1 acts genetically with the KIX-PPD-MYC complex to control seed size. **a**, **b** The seeds (**a**) and 8-day-old seedlings (**b**) of Col-0, *gif1*, *myc3 myc4*, *gif1 myc3 myc4*, *kix8*-*1 kix9*-*1*, *gif1 kix8*-*1 kix9*-*1*, *ppd1*-*2 ppd2*-*cr*, *gif1 ppd1*-*2 ppd2*-*cr*, *35S*:*SAP*, and *35S:SAP*;*gif1* plants. **c**–**e** The relative seed area (**c**, *n* = 100), 100 seed weight (**d**, *n* = 10), and cotyledon area (**e**, *n* = 30) of Col-0, *gif1*, *myc3 myc4*, *gif1 myc3 myc4*, *kix8*-*1 kix9*-*1*, *gif1 kix8*-*1 kix9*-*1*, *ppd1*-*2 ppd2*-*cr*, *gif1 ppd1*-*2 ppd2*-*cr*, *35S*:*SAP*, and *35S:SAP*;*gif1* plants. **f** The TPL-KIX-PPD-MYC complex associates with the G-box sequence of *GIF1* promoter and represses its expression. **g** The transcriptional repression of *GIF1* is relieved by SAP modulating the KIX-PPD module for 26S proteasome degradation. Without the KIX-PPD complex, the binding ability of MYC3/4 with the promoter of *GIF1* is decreased and the *GIF1* expression is increased. Seeds from the third to seventh silique on the stem of six plants were used for analysis. Cotyledons from the 8-day-old seedlings were used for analysis. Scale bars, 0.5 mm (**a**) and 0.2 cm (**b**). Error bars represent ±SE. Different lowercase letters above the columns indicate the significant difference among different groups, one-way ANOVA *P*-values: *P* < 0.05.

As the KIX-PPD-MYC complex associates with the promoter of *GIF1* through MYC3/4 and represses its expression, we asked whether GIF1 act in a common pathway with the KIX-PPD-MYC module to control seed size. We crossed *gif1* with *myc3 myc4*, *kix8*-*1 kix9*-*1*, and *ppd1*-*2 ppd2*-*cr* to generate *gif1 myc3 myc4*, *gif1 kix8*-*1 kix9*-*1*, and *gif1 ppd1*-*2 ppd2*-*cr* triple mutants, respectively. As shown in Fig. 7a–c, e, the *gif1* mutation entirely suppressed the large seed and cotyledon phenotypes of *myc3 myc4*, indicating that *gif1* is epistatic to *myc3 myc4* with respect to seed and cotyledon size. Similarly, *gif1* is also epistatic to *myc3 myc4* with respect to seed weight (Fig. 7d). The large size of seeds and cotyledons of *kix8*-*1 kix9*-*1* and *ppd1*-*2 ppd2*-*cr* plants was strongly but not entirely suppressed by the *gif1* mutation (Fig. 7a–c, e), indicating that KIX8/9 and PPD1/2 are strongly but not entirely dependent on GIF1 to control seed size. Similarly, the *gif1* mutation strongly but not entirely suppressed the seed weight phenotype of *kix8*-*1 kix9*-*1* and *ppd1*-*2 ppd2*-*cr* plants (Fig. 7d). These genetic analyses indicate that GIF1 acts in a common pathway with the KIX-PPD-MYC module to control seed size and weight.

The *ppd1*-*2 ppd2*-*cr* and *kix8*-*1 kix9*-*1* plants produced wider siliques than wild-type plants. By contract, *gif1* plants had narrower siliques than wild-type plants (Supplementary Fig. 15). The *gif1* mutation entirely suppressed the wide silique phenotype of *kix8*-*1 kix9*-*1* plants and strongly suppressed the wide silique phenotype of *ppd1*-*2 ppd2*-*cr* plants (Supplementary Fig. 15). In addition, the silique phenotype of *gif1 myc3 myc4* was similar to that of *gif1* (Supplementary Fig. 15). These genetic analyses indicate that GIF1 acts genetically with the KIX-PPD-MYC module to control silique development. The *gif1* and *ppd1*-*2 ppd2*-*cr* plants had lower fertility than wild-type plants (Supplementary Fig. 16). The *myc3 myc4* and *kix8*-*1 kix9*-*1* had similar fertility to wild-type plants, but the fertility of *gif1 myc3 myc4* and *gif1 kix8*-*1 kix9*-*1* was similar to that of *gif1* plants (Supplementary Fig. 16). Additionally, the *gif1* mutation decreased the fertility of *ppd1*-*2 ppd2*-*cr* plants (Supplementary Fig. 16). All of the genetic analyses indicate that GIF1 acts with the KIX-PPD-MYC module to control multiple biological processes.

### *GIF1* and *SAP* function in a common pathway to control seed size

STERILE APETALA (SAP/SUPPRESSOR OF DA1, SOD3) acts as a part of the E3 ubiquitin ligase complex to control organ size by regulating the stability of the KIX-PPD complex^22,23^. The *sod3*-*1* mutants produce small leaves, while *35S*:*SAP* plants have large leaves^22,23^. We found that *35S*:*SAP* plants also produced bigger seeds and cotyledons than the wild-type plants (Fig. 7a–c, e). Similarly, *35S*:*SAP* plants produced heavier seeds than wild-type plants (Fig. 7d). The *gif1* mutation strongly suppressed the large seed and cotyledon phenotype of *35S*:*SAP* plants (Fig. 7a–c, e). The *gif1* mutation also strongly suppressed the heavy seed phenotype of *35S*:*SAP* plants (Fig. 7d). Additionally, *35S*:*SAP* plants produced wider siliques than wild-type plants, while it was significantly suppressed by the *gif1* mutation (Supplementary Fig. 15). Although *35S*:*SAP* plants had similar fertility to wild-type plants, the fertility of *35S*:*SAP*;*gif1* plants was similar to that of *gif1* (Supplementary Fig. 16). Moreover, the *GIF1* expression was obviously higher in the 3 DAP siliques of *35S*:*SAP* plants than that in wild-type plants (Supplementary Fig. 17). These data indicate that GIF1 acts as a downstream factor of SAP to control seed size.

## Discussion

Seed size is the key agronomic trait that greatly determines the grain yield of plants. Although several factors have been reported to affect seed size in plants^4,5^, the genetic and molecular mechanisms that determine seed size remain elusive. In this study, we discover a genetic and molecular mechanism that the transcription factors MYC3/4 recruit the repressor complex KIX8/9-PPD1/2 to the promoter of *GIF1* and repress its expression, thereby determining seed size in Arabidopsis.

Previous studies reported that PPD1 and PPD2 act redundantly to regulate leaf size and shape by influencing both the primary and the secondary mitotic arrest fronts^20,21,23^. Considering that *ppd2*-*1* had large seeds, while *ppd1*-*2* did not obviously affect seed size, *ppd1*-*2 ppd2*-*1* double mutant will help understand the role of *PPD1*/*2* in seed size control. However, the *PPD1* gene (*AT4G14713*) is close to the *PPD2* gene (*AT4G14720*) in the chromosome, we could not isolate *ppd1*-*2 ppd2*-*1* double mutant. We therefore generated the *ppd2*-*cr* mutation in the *ppd1*-*2* mutant background and the *ppd1*-*cr* mutation in the *ppd2*-*1* mutant background to obtain the *ppd1*-*2 ppd2*-*cr* and *ppd1*-*cr ppd2*-*1* double mutants using the CRISPR-Cas9 technology, respectively (Supplementary Fig. 1)^27^. The *ppd1*-*2 ppd2*-*cr* and *ppd1*-*cr ppa2*-*1* mutants produced larger and heavier seeds than *ppd1*-*2* and *ppd2*-*1* single mutants (Fig. 1a, c, d), indicating that PPD1 and PPD2 function redundantly to control seed size and weight. KIX8/9 interact with PPD1/2 to form a transcriptional repressor complex and control leaf size^21,22^. However, it is unclear whether the KIX-PPD complex affects seed growth in Arabidopsis. Here, we found that *kix8*-*1 kix9*-*1 ppd1*-*2 ppd2*-*cr* plants produced significantly larger seeds than the wild type (Fig. 1a, c). Reciprocal crossing experiments showed that the KIX-PPD complex functions maternally to control seed size (Fig. 1f, g). Cellular observation indicated that the KIX-PPD complex predominantly represses cell proliferation and also slightly limiting cell elongation in the integuments (Fig. 3k, l). These results reveal that the KIX-PPD complex negatively regulates seed growth in Arabidopsis.

The transcriptional repressor complex usually interacts with the transcription factors to regulate gene expression^43,44^. We found that PPD1/2 could directly interact with the transcription factors MYC3/4 in vitro and in vivo, but not interact with MYC2. Although MYC3 and MYC4 share lots of overlapping functions with MYC2, distinct functions among them have been reported. For instance, MYC3 and MYC4 recognise similar *cis*-acting sequences (i.e. G-box and its variants) to MYC2, while the DNA-binding affinity of MYC3 and MYC4 differs from that of MYC2. MYC2 and MYC4 but not MYC3 interact with the JAZ4 protein. The expression levels of *VEGETATIVE STORAGE PROTEIN 2* (*VSP2*) and *PLANT DEFENSIN 1.2* (*PDF1.2*), two of JA marker genes, are significantly different in *myc2*, *myc3*, and *myc4* single mutants when treated with JA^33,34,45^. We further reveal that KIX8/9, PPD1/2, and MYC3/4 can form a complex in Arabidopsis (Fig. 2). Like *kix8*-*1 kix9*-*1* and *ppd1*-*2 ppd2*-*cr* mutants, *myc3 myc4* mutants produced bigger seeds than the wild type (Fig. 3a, c), consistent with a previous study^36^, further suggesting that MYC3/4 have the overlapped function with KIX8/9 and PPD1/2 in seed size control. Reciprocal crossing experiments indicate that MYC3/4 act maternally to limit seed growth. Cellular observations show that MYC3/4 influence both cell proliferation and cell elongation in the integuments, consistent with the role of the KIX-PPD complex in seed growth control. Therefore, the KIX-PPD-MYC module is crucial for seed size control in Arabidopsis.

To identify the targets of the KIX-PPD-MYC module in seed growth, we performed the RNA-seq and found that PPD1/2 and MYC3/4 repress the expression of *GIF1*. The expression levels of *GIF1* in 0, 2, and 4 DAF siliques from *kix8*-*1 kix9*-*1*, *ppd1*-*2 ppd2*-*cr* and *myc3 myc4* plants were significantly higher than those of wild-type plants (Fig. 4a). Consistent with this, overexpression of Myc-KIX8/9, Myc-PPD1/2, Myc-MYC3/4, and Myc-TPL could reduce the activity of *GIF1pro*:*LUC* (Fig. 4b). In addition, EMSA experiments showed that MYC3 and MYC4 directly bind to the G-box sequence in the promoter of *GIF1* (Fig. 4f, g). ChIP-qPCR analyses showed that KIX8/9 and PPD1/2 associate with the promoter of *GIF1* through MYC3/4 (Fig. 4d). These findings indicate that MYC3/4 may recruit the transcriptional repressor complex TPL-KIX-PPD to the promoter of *GIF1* to repress its expression (Fig. 7f). Overexpression of *KIX8*, *KIX9*, *PPD1*, *PPD2*, *MYC3*, or *MYC4* led to small seeds (Supplementary Figs. 5 and 6), consistent with the result of *PPD1OE* (*PPDOE*)^19^. It is possible that overexpression of *KIX8*, *KIX9*, *PPD1*, *PPD2*, *MYC3*, or *MYC4* in Arabidopsis might have more probability to form the TPL-KIX-PPD-MYC complex that represses the expression of *GIF1*, thereby resulting in small seeds. Interestingly, MYC proteins recruit the TPL-NINJIA-JAZ transcriptional repressor complex to regulate gene expression^43,46,47^. Overexpression of JAZ13 alone attenuates JA-induced defence responses in Arabidopsis leaves. Overexpression of NINJIA promotes root length when treated with MeJA^43^. Overexpression of MYC2, MYC3, or MYC4 accelerates JA-induced leaf senescence^46^. These results indicate that overexpression of the single complex components can cause phenotypes. The expression of *GIF2* and *GIF3* in the 3 DAP siliques of *kix8*-*1 kix9*-*1*, *ppd1*-*2 ppd2*-*cr*, and *myc3 myc4* plants was also upregulated compared with that in the wild type (Supplementary Fig. 18). Down-regulation of *PPDs* orthologs in legume *Medicago truncatula* and legume soybean leads to high expression of *MtGIF1* and *GmGIF1* in leaves, stipules, and seeds, respectively^40^. These results indicate that the expression of *GIFs* regulated by PPDs might be a common mechanism in dicotyledon plants. The loss-of-function mutation in *GIF1* produced smaller seeds and cotyledons than the wild type (Fig. 5a–c, e), consistent with previous studies^10,11^. The *gif1* mutant has fewer cells and longer cells in the integuments than the wild type (Fig. 5k, l), suggesting a compensation mechanism between cell proliferation and cell elongation. This phenomenon has been observed in several seed size mutants^28,41,42^. In addition, GIF1 was reported to play significant roles in leaf, flower, and root development in Arabidopsis^8–14^, indicating that GIF1 is required for normal plant organ growth. Surprisingly, a previous study showed that one mutant allele of *GIF1* (*an3*) promotes seed growth^48^. In this study, we have sufficient evidence to support that GIF1 is a positive regulator of seed size in Arabidopsis. For example, loss-of-function of *GIF1* formed small seeds, while overexpression of *GIF1* produced large seeds (Fig. 5a, c). The *gif1* mutation completely suppresses the large and heavy seed phenotypes of *myc3 myc4* (Fig. 7c, d). By contrast, the *gif1* mutation strongly but not entirely suppresses the seed size and weight phenotypes of *kix81*-*1 kix91*-*1* and *ppd1*-*2 ppd2*-*cr* (Fig. 7c, d), implying that *KIX8*/*9* and *PPD1*/*2* might have other mechanisms that act independently of *GIF1* to control seed development. These genetic analyses also reveal that GIF1 functions in a common pathway with the KIX-PPD-MYC module to control seed size in Arabidopsis. Consistent with this, *KIX8*, *KIX9*, *PPD1*, *PPD2*, *MYC3*, *MYC4* and *GIF1* have overlapped expression patterns during ovule development and possess partially overlapped expression patterns during early seed developmental stages (Fig. 6 and Supplementary Fig. 14).

STERILE APETALA (SAP/SUPPRESSOR OF DA1, SOD3) acts as a part of the E3 ubiquitin ligase complex to control organ size by regulating the stability of the PPD-KIX complex^22,23^. The expression of *GIF1* was obviously higher in *35S*:*SAP* plants than that in wild-type plants (Supplementary Fig. 17), indicating that the transcriptional repression of *GIF1* is released by the F-box protein SAP that modulates the KIX-PPD complex for 26S proteasome degradation (Fig. 7g). However, SAP did not modulate the stability of MYC3/4 proteins (Supplementary Fig. 19). Without the KIX-PPD complex, MYC3/4 could bind to the promoter of *GIF1*, but the binding ability is significantly decreased (Fig. 4d). Genetic analyses showed that the *gif1* mutation strongly suppresses the large and heavy seed phenotypes of *35S*:*SAP* (Fig. 7c, d), suggesting that *SAP* and *GIF1* act in a common pathway to regulate seed growth. Based on these genetic and biochemical analyses, we build up a genetic and molecular framework for the SAP-KIX-PPD-MYC-GIF1 module-mediated control of seed size and weight in Arabidopsis (Fig. 7f, g).

Seed size is one of the important targets for plant breeding. In this study, we found that the KIX-PPD-MYC-GIF1 pathway is crucial for seed size control in Arabidopsis. Interestingly, loss-of-function of PPD orthologs in legume *Medicago truncatula* and legume soybean increases seed size and weight as well as leaf size^40^. In pea, mutations in the PPD ortholog ELEPHANT-EAR-LIKE LEAF 1 or the KIX ortholog BIGGER ORGANS cause large flowers and leaves^49^. In rice, overexpression of *OsGIF1* results in large grains, leaves, and stems, while suppression of *OsGIF1* leads to small grains and organs^16,17^. These findings suggest that the KIX-PPD-MYC-GIF1 pathway may possess a conserved function in different plant species. Thus, it will be interesting to investigate the roles of the KIX-PPD-MYC-GIF1 pathway in crops and utilise their homologues to improve seed size in key crops.

## Methods

### Plant material and growth conditions

All of the mutants and transgenic plants were in *Arabidopsis thaliana* Col-0 ecotype. The seeds of *ppd1*-*2* (SALK_057237), *ppd2*-*1* (SALK_142698), *kix8*-*1* (GABI_422H04), *kix9*-*1* (SAIL_1168_G09), *gif1* (SALK_150407), *myc3* (GK_445B11) and *myc4* (GK_491E10) were obtained from the ABRC or NASC, and identified by PCR with T-DNA specific and flanking primers (Supplementary Table 1). *kix8-1 kix9-1* plants were obtained by crossing *kix8*-*1* with *kix9-1* plants and identified by PCR with primers of kix8-1F/1R and kix9-1F/1R (Supplementary Table 1). *gif1 kix8-1 kix9-1* plants were obtained by crossing *kix8-1 kix9-1* with *gif1* plants and identified by PCR with primers of kix8-1F/1R, kix9-1F/1R and gif1-F/R (Supplementary Table 1). *myc3 myc4* plants were obtained by crossing *myc3* with *myc4* plants and identified by PCR with primers of myc3-F/R and myc4F/R (Supplementary Table 1). *gif1 myc3 myc4* plants were obtained by crossing *myc3 myc4* with *gif1* plants and identified by PCR with primers of myc3-F/R, myc4F/R and gif1-F/R (Supplementary Table 1). *35S*:*SAP* plants were described before^23^.

The *ppd1*-*2 ppd2*-*cr* and *ppd1*-*cr ppd2*-*1* mutants were obtained by CRISPR-Cas9 mediated genome editing^27^. *PPD2*-*gRNA* and *PPD1*-*gRNA* was cloned into the *pBluescript*-*AtU6*-*SK* vector, and then *AtU6*-*gRNA* sequence was transferred to the *pCAMBIA1300*-*pYAO*:*Cas9* vector to generate *pCAMBIA1300*-*pYAO*:*Cas9*-*AtU6*-*gRNA* constructs. The final constructs were transferred into *ppd1*-*2* or *ppd2*-*1* plants by agrobacterium tumefaciens-mediated transformation^50^. Transgenic plants were screened out with 30 μg ml^−1^ hygromycin. Genome-edited *ppd1*-*2 ppd2*-*cr* and *ppd1*-*cr ppd2*-*1* mutants were identified by the sequencing of the PCR products with PPD1 and PPD2 specific primers (PPD1-CRIJD-F/R and PPD2-CRIJD-F/R) (Supplementary Fig. 1, Supplementary Table 1). *gif1 ppd1*-*2 ppd2*-*cr* plants were obtained by crossing *ppd1*-*2 ppd2*-*cr* with *gif1* plants. The *gif1* and *ppd1*-*2* mutations in *gif1 ppd1*-*2 ppd2*-*cr* plants were identified by PCR with primers of gif1-F/R and ppd1-2F/R. The *ppd2*-*cr* mutation in *gif1 ppd1*-*2 ppd2*-*cr* plants were identified by the sequencing of the PCR products with PPD2 specific primers (PPD2-CRIJD-F/R) (Supplementary Table 1).

The CDS of *GIF1* was obtained from the total RNA of the Col-0 plants with FastQuant RT Super Mix kit (TIANGEN, KR108) and cloned into the Kpn I and Spe I sites of *pMDC32* vector to generate *pMDC32*-*35S*:*GIF1* constructs with EZfusion kit (Genera, GR6086). *pMDC32*-*35S*:*GIF1* constructs were transferred into the Col-0 plants by agrobacterium tumefaciens-mediated transformation^50^. *35S*:*GIF1* transgenic plants were screened out with 30 μg ml^−1^ hygromycin.

Seeds were sterilised with ethanol (75% v/v) for 3 min, bleach (10% v/v) for 15 min, and washed with sterilised water three times, and then plated to Murashige and Skoog (MS) medium. After storing in dark for 4 days at 4 °C, seeds were grown at 22 °C with 16 h light (28 W/6500 K)/8 h dark.

### Plant transformation and screen

The transgenic plants were obtained by agrobacterium tumefaciens-mediated transformation^50^. Constructs were transferred into GV3101 agrobacterium cells. Agrobacterium cells were grown in LB medium containing 1% (m/v) Trypton, 0.5% (m/v) Yeast Extract, and 1% (m/v) NaCl (pH 7) at 28 °C overnight. Bacteria were pelleted and resuspended to 0.8 OD_600_ concentration with the solution containing 0.22% (m/v) MS, 0.05% (v/v) Silwet L-77, 5% (m/v) sucrose, and 0.02% (m/v) MES (pH 5.7). Inflorescences of Col-0 plants were used for transformation. After transformation, plants were immediately covered with plastic bags and grew overnight before bags were removed. The transgenic plants were screened out with 30 μg ml^−1^ hygromycin.

### Morphological and cellular analysis

Seeds were harvested from the third to seventh silique on the stem of plants. Cotyledons were harvested from the 8-day-old seedlings. Siliques were harvested at 14 DAF (days after flowering). Parameters of seeds, cotyledons, and siliques were measured by ImageJ software after photographing. For seed weight, 100 seeds were weighed at each experiment by Mettler Toledo XP6 (Mettler Toledo, Switzerland). As for seed integument observation, the stamens in the fourth flower on the stem of plants were removed before flowering. The plants were pollinated with their own pollens. The seeds were harvested at 0, 2, 4 and 6 DAP (days after pollination). Ovules from six siliques, which were from the fourth silique on the stem of six plants, were used for analysis. At least five representative ovules were analysed. Seeds from six siliques, which were from the fourth silique on the stem of six plants, were used for analysis. At least five representative seeds in a silique were used for analysis. Seeds were firstly cleared with FAA solution (90 mL 70% ethanol, 5 mL acetic acid, and 5 mL 37% formaldehyde), and then dealt with Hoyer’s solution (7.5 g gum arabic, 100 g chloral hydrate, 5 mL glycerol, 5 mL phenol, and 25 mL water) before used to observation under the differential interference contrast microscope (DIC, Leica DM2500). The integument cell number of seeds was counted in DIC. The integument length of seeds was matured by ImageJ.

### Subcellular localisation analysis

The promotors of *KIX8* (2,087 bp), *KIX9* (1,714 bp), *PPD1* (1,797 bp), *PPD2* (2,153 bp), *MYC3* (2,180 bp), *MYC4* (2,132 bp), and *GIF1* (2,337 bp) and their CDSs were cloned into the Asc I and Xba I sites of *pMDC107*-*GFP* vector to generate *KIX8pro*:*KIX8*-*GFP*, *KIX9pro*:*KIX9*-*GFP*, *PPD1pro*:*PPD1*-*GFP, PPD2pro*:*PPD2*-*GFP*, *MYC3pro*:*MYC3*-*GFP*, *MYC4pro*:*MYC4*-*GFP*, and *GIF1pro*:*GIF1*-*GFP* constructs with EZfusion kit (Genera, GR6086), respectively. The constructs of *KIX8pro*:*KIX8*-*GFP*, *KIX9pro*:*KIX9*-*GFP*, *PPD1pro*:*PPD1*-*GFP*, *PPD2pro*:*PPD2*-*GFP*, *MYC3pro*:*MYC3*-*GFP*, *MYC4pro*:*MYC4*-*GFP*, and *GIF1pro*:*GIF1*-*GFP* were transferred into Col-0 plants by agrobacterium tumefaciens-mediated transformation. Transgenic plants were screened out with 30 μg ml^−1^ hygromycin. The GFP fluorescence in ovules and seeds was observed by the confocal microscopy (LSM710, Zeiss, Germany).

### Split luciferase complementation assays

The CDSs of *MYC2*, *MYC3* and *MYC4* were obtained from the total RNA of the Col-0 plants with FastQuant RT Super Mix kit (TIANGEN, KR108) and cloned into the Sal I site of *pCAMBIA*-*split_nLUC* vector with EZfusion kit (Genera, GR6086). The CDSs of *KIX8*, *KIX9*, *PPD1*, and *PPD2* were obtained from the total RNA of the Col-0 plants with FastQuant RT Super Mix kit (TIANGEN, KR108) and cloned into the Kpn I site of *pCAMBIA*-*split_cLUC* vector with EZfusion kit (Genera, GR6086). Split luciferase complementation assay was conducted as described previously^51^. Constructs were transferred into GV3101 agrobacterium cells. Agrobacterium cells were grown in LB medium containing 1% (m/v) Trypton, 0.5% (m/v) Yeast Extract, and 1% (m/v) NaCl (pH 7) at 28 °C to 0.8 OD_600_ concentration. Bacteria were pelleted and resuspended to 0.5 OD_600_ concentration with the solution containing 10 mM MES (pH 5.7), 10 mM MgCl_2_, and 150 mM Acetosyringone. The combinations of *MYC2*-*nLUC*/*cLUC*-*PPD1*/*2*, *MYC2*-*nLUC*/*cLUC*-*KIX8*/*9*, *MYC3*-*nLUC*/*cLUC*-*PPD1*/*2*, *MYC3*-*nLUC*/*cLUC*-*KIX8*/*9*, *MYC4*-*nLUC*/*cLUC*-*PPD1*/*2*, *MYC4*-*nLUC*/*cLUC*-*KIX8*/*9*, *MYC2*/*3*/*4*-*nLUC*/*cLUC*, *nLUC*/*cLUC*-*PPD1*/*2* and *nLUC*/*cLUC*-*KIX8*/*9* were transferred into *N. benthamiana* leaves by agrobacterium tumefaciens-mediated transformation. The luciferase activity was detected 2 days later after infiltration. One millimolar luciferin (Sigma, 11626353001) was sprayed onto leaves, and the materials were kept in dark for 5 min. Images were obtained with CCD imaging apparatus (CHEMIPROHT 1300B/LND; Roper Scientific).

### FRET-FLIM analysis

The CFP and YFP sequences were cloned into the Sac I site of *pMDC32* vector to generate *pMDC32*-*CFP*/*YFP* constructs with EZfusion kit (Genera, GR6086). The CDSs of *MYC3* and *MYC4* were obtained from the total RNA of the Col-0 plants with FastQuant RT Super Mix kit (TIANGEN, KR108) and cloned into the Sac I and Kpn I sites of *pMDC32*-*CFP* construct to generate *pMDC32*-*MYC3*/*MYC4*-*CFP* constructs with EZfusion kit (Genera, GR6086). The CDSs of *PPD1*, *PPD2*, and DEL1 were obtained from the total RNA of the Col-0 plants with FastQuant RT Super Mix kit (TIANGEN, KR108) and cloned into the Sac I and Kpn I sites of *pMDC32*-*YFP* construct to generate *pMDC32*-*PPD1*/*PPD2*/*DEL1*-*YFP* constructs with EZfusion kit (Genera, GR6086). Constructs were transferred into GV3101 agrobacterium cells. Agrobacterium cells were grown in LB medium containing 1% (m/v) Trypton, 0.5% (m/v) Yeast Extract, and 1% (m/v) NaCl (pH 7) at 28 °C to 0.8 OD_600_ concentration. Bacteria were pelleted and resuspended to 0.5 OD_600_ concentration with the solution containing 10 mM MES (pH 5.7), 10 mM MgCl_2_, and 150 mM Acetosyringone. The different combinations of *35S*:*MYC3*-*CFP*, *35S*:*MYC4*-*CFP*, *35S*:*PPD1*-*YFP*, *35S*:*PPD2*-*YFP* and *35S*:*DEL1*-*YFP* constructs were coinfiltrated into *N. benthamiana* leaves by GV3101 agrobacterium cells. CFP fluorescence lifetime was obtained at 2 days later after coinfiltration by the confocal microscopy (LSM710, Zeiss, Germany) and TCSPC module and picosecond event timer (PicoQuant, Germany).

### Bimolecular fluorescence complementation assays

The *cYFP*-*MYC3*/*4* and *nYFP*-*PPD1*/*2* constructs were generated by transferring the CDSs of *MYC3*/*4* and *PPD1*/*2* into the Xba I and SalI sites of *pGWB414*-*cYFP* and *pGWB414*-*nYFP* vectors with EZfusion kit (Genera, GR6086), respectively. Constructs were transferred into GV3101 agrobacterium cells. Agrobacterium cells were grown in LB medium containing 1% (m/v) Trypton, 0.5% (m/v) Yeast Extract, and 1% (m/v) NaCl (pH 7) at 28 °C to 0.8 OD_600_ concentration. Bacteria were pelleted and resuspended to 0.5 OD_600_ concentration with the solution containing 10 mM MES (pH 5.7), 10 mM MgCl_2_, and 150 mM Acetosyringone. The combinations of *cYFP*-*MYC3*/*nYFP*-*PPD1*/*2*, *cYFP*-*MYC4*/*nYFP*-*PPD1*/*2*, *cYFP*-*MYC3*/*4*/*nYFP*, and *cYFP*/*nYFP*-*PPD1*/*2* were coinfiltrated into *N. benthamiana* leaves by GV3101 agrobacterium cells. The YFP fluorescence was observed with the confocal microscope (LSM710, Zeiss, Germany) 2 days later after infiltration. 4′,6-diamidino-2-phenylindole (DAPI, Sigma, D9542) with 2 µg/ml was used to stain the nuclei.

### Pull-down assays

The CDSs of *MYC3* and *MYC4* were obtained from the total RNA of the Col-0 plants with FastQuant RT Super Mix kit (TIANGEN, KR108) and cloned into the EcoR I site of *pGEX4T1*-*GST* vector to generate *GST*-*MYC3*/*4* constructs with EZfusion kit (Genera, GR6086). The CDSs of *PPD1* and *PPD2* were obtained from the total RNA of the Col-0 plants with FastQuant RT Super Mix kit (TIANGEN, KR108) and cloned into the BamH I site of *pMALC2*-*MBP* vector to generate *MBP*-*PPD1*/*2* constructs with EZfusion kit (Genera, GR6086). The CDSs of *KIX8* and *KIX9* were obtained from the total RNA of the Col-0 plants with FastQuant RT Super Mix kit (TIANGEN, KR108) and cloned into the Nco I site of *pET24a*-*His* vector to generate *His*-*KIX8*/*9* constructs with EZfusion kit (Genera, GR6086). Pull-down assays were carried out as a previous study^52^. Constructs were transferred into *E. coli* BL21 (DE3) cells. All proteins were expressed in *E. coli* BL21 (DE3) with 0.5 mM Isopropyl β-d-1-thiogalactopyranoside (IPTG) at 28 °C for 4 h. BL21 (DE3) cells were pelleted and resuspended with the solution containing 50 mM HEPES (pH 7.5), 150 mM NaCl, 1 mM EGTA, 1% (v/v) Triton X-100, 10% (v/v) glycerol, and 1 mM PMSF. Proteins were obtained from bacteria after sonicating for 5 min (5 s on, 10 s stop) at 20 amplitude. The combinations of GST-MYC3/4, MBP-PPD1/2, and His-KIX8/9 proteins were incubated at 4 °C for 1 h and pulled down with anti-MBP agarose beads (NEB, E8037s) or Ni-NTA agarose beads (Invitrogen, R90115). Beads were washed 4 times with the solution containing 50 mM HEPES (pH 7.5), 150 mM NaCl, 1 mM EGTA, 0.5% (v/v) Triton X-100, 10% (v/v) glycerol, and 1 mM PMSF. Proteins were extracted with the solution containing 250 mM Tris-HCl (pH 6.8), 10% (w/v) SDS, 30% (v/v) glycerol, 5% (v/v) mercaptoethanol, 0.02% (m/v) bromophenol blue after boiling at 98 °C for 5 min. Proteins were detected by western blot with anti-GST (1:5000, Abmart, M20007), anti-His (1:2000, Abmart, M20001), or anti-MBP antibody (1:5000, Abmart, T40007).

### Co-immunoprecipitation

The *35S*:*GFP*-*MYC2*/*3*/*4* constructs were generated by transferring the CDSs of *MYC2*/*3*/*4* into the Asc I and Sal I sites of *pMDC43* vector with EZfusion kit (Genera, GR6086). *35S*:*Myc*-*KIX8*/*9* and *35S*:*Myc*-*PPD1*/*2* constructs were generated by transferring the CDSs of *KIX8*/*9* and *PPD1*/*2* into the Kpn I site of *pCambia1300*-*221*-*Myc* vector*. 35S*:*GFP*-*MYC2*/*3*/*4*, *35S*:*Myc*-*KIX8*/*9*, and *35S*:*Myc*-*PPD1*/*2* transgenic Arabidopsis plants were obtained by agrobacterium tumefaciens-mediated transformation. *35S*:*Myc*-*PPD1*/*2*;*35S*:*GFP*-*MYC2*/*3*/*4*, *35S*:*Myc*-*KIX8*/*9*;*35S*:*GFP*-*MYC3*/*4*, *35S*:*Myc*-*PPD1*/*2*;*35S*:*GFP*, and *35S*:*Myc*-*KIX8*/*9*;*35S*:*GFP* plants were obtained by crossing *35S*:*Myc*-*PPD1*/*2* or *35S*:*Myc*-*KIX8*/*9* with *35S*:*GFP*-*MYC2*/*3*/*4* and *35S*:*GFP*, respectively. The co-immunoprecipitation was conducted as described previously. Plants were ground with liquid nitrogen and proteins were dissolved with the buffer (50 mM Tris-HCl, 20% (v/v) glycerol, 150 mM NaCl, 2% (v/v) Triton X-100, 1 mM EDTA, 1× protease inhibitor cocktail, pH 7.5). The combinations of GFP, GFP-MYC2/3/4, Myc-KIX8/9, and Myc-PPD1/2 proteins were incubated at 4 °C for 1 h and pulled down with GFP-Trap agarose beads (Qualityard, QYA03914AAC). Beads were washed 4 times with the solution containing 50 mM Tris-HCl (pH 7.5), 20% (v/v) glycerol, 150 mM NaCl, 0.5% (v/v) Triton X-100, 1 mM EDTA, and 1× protease inhibitor cocktail. Proteins were extracted with the solution containing 250 mM Tris-HCl (pH 6.8), 10% (w/v) SDS, 30% (v/v) glycerol, 5% (v/v) mercaptoethanol, 0.02% (m/v) bromophenol blue after boiling at 98 °C for 5 min. Proteins were detected by western blot with anti-Myc (1:5000, Abmart, M20002) or anti-GFP antibody (1:8000, Invitrogen, MA5-15256).

### qPCR and RNA-Seq analysis

Total RNA was extracted according to the procedure of RNAprep pure kit (TIANGEN, DP439-H). The first cDNA was produced according to the procedure of FastQuant RT Super Mix kit (TIANGEN, KR108). qPCR was conducted with the SYBR Green І (ROCHE) by the realplex^2^ (eppendorf). Data were normalised with *ACTIN2*. For RNA-seq, the first pair of leaves from 9-day-old *myc3 myc4* and *ppd1*-*2 ppd2*-*cr* seedlings were harvested. The RNA extraction, sequencing, and analysis were conducted by Biomarker Technologies Corporation (China). Three biological replicates were performed for RNA-seq analyses. In brief, total RNA was extracted according to the procedure of RNeasy Plant Mini Kit (Qiagen). RNA-seq was performed with the Illumina HiSeq X platform (Illumina Inc., San Diego, CA) using 150-bp double-ended reads. The reads were aligned to the Arabidopsis reference genome (version TAIR10) using TopHat (version 2.0.12). *P*-values were adjusted using the Benjamini-Hochberg procedure. Differentially expressed genes were defined based on the *P*-values: *P* < 0.05.

### Dual-luciferase transient expression assay

Dual-luciferase transient expression assays were carried out in the Arabidopsis protoplast. The CDSs of *KIX8*, *KXI9*, *PPD1*, *PPD2*, *MYC3*, *MYC4*, and *TOPLESS* were cloned into the Kpn I site of *pCambia1300*-*221*-*Myc* vector to generate *35S*:*Myc*-*KIX8*, *35S*:*Myc*-*KIX9*, *35S*:*Myc*-*PPD1*, *35S*:*Myc*-*PPD2*, *35S*:*Myc*-*MYC3*, *35S*:*Myc*-*MYC4*, and *35S*:*Myc*-*TOPLESS* constructs with EZfusion kit (Genera, GR6086). The 2337 bp sequence of *GIF1* promoter was cloned into the Hind III site of *pGreen II_0800*-*LUC* vector to generate the *GIF1pro*:*LUC* contruct with EZfusion kit (Genera, GR6086). Arabidopsis protoplast was isolated as described before. *GIF1pro*:*LUC* was cotransfected with different combinations of *35S*:*Myc*-*KIX8*, *35S*:*Myc*-*KIX9*, *35S*:*Myc*-*PPD1*, *35S*:*Myc*-*PPD2*, *35S*:*Myc*-*MYC3*, *35S*:*Myc*-*MYC4*, and *35S*:*Myc*-*TOPLESS* into the Arabidopsis protoplast. Protoplasts were incubated at 23 °C for 36 h. LUC and REN luciferase activities were measured using the dual luciferase assay kit (Promega, E1500). The analysis was performed using the Luminoskan Ascent Microplate Luminometer (Thermo Fisher Scientific).

### Chromatin immunoprecipitation analysis

In all, 1–4 DAF siliques *of 35S*:*GFP*, *35S*:*GFP*-*MYC3*;*myc3*, *35S*:*GFP*-*MYC4*;*myc4*, *35S*:*GFP*-*MYC3*;*myc3 ppd1*-*2 ppd2*-*cr*, *35S*:*GFP*-*MYC4*;*myc4 ppd1*-*2 ppd2*-*cr*, *35S*:*GFP*-*MYC3*;*myc3 kix8*-*1 kix9*-*1*, *35S*:*GFP*-*MYC4*;*myc4 kix8*-*1 kix9*-*1*, *35S*:*GFP*-*PPD1*;*ppd1*-*2*, *35S*:*GFP*-*PPD2*;*ppd2*-*1*, *35S*:*GFP*-*PPD1*;*ppd1*-*2 myc3 myc4*, *35S*:*GFP*-*PPD2*;*ppd2*-*1 myc3 myc4*, *35S*:*GFP*-*KIX8*;*kix8*-*1*, *35S*:*GFP*-*KIX9*;*kix9*-*1*, *35S*:*GFP*-*KIX8*;*kix8*-*1 myc3 myc4* and *35S*:*GFP*-*KIX9*;*kix9*-*1 myc3 myc4* were used for Chromatin extraction. The *35S*:*GFP*-*KIX8*;*kix8*-*1*, *35S*:*GFP*-*KIX9*;*kix9*-*1*, *35S*:*GFP*-*PPD1*;*ppd1*-*2*, *35S*:*GFP*-*PPD2*;*ppd2*-*1*, *35S*:*GFP*-*MYC3*;*myc3* and *35S*:*GFP*-*MYC4*;*myc4* plants with small seeds were used for the analysis (Supplementary Fig. 12). Chromatin immunoprecipitation analyses (ChIP-qPCR) were carried out as described before^53^. Siliques were cross-linked with the buffer (0.4 M sucrose, 15 mM PIPESl (pH 6.8), 1 mM EDTA, 1 mM PMSF, 1% (v/v) formaldehyde) and vacuumized for 15 min at room temperature. The cross-linking was stop by adding 2 M glycine to final concentration of 100 mM. Siliques were washed three times in sterile deionized water and ground to a fine powder with liquid nitrogen. Nuclei were isolation with the buffer (0.25 M sucrose, 15 mM PIPES (pH 6.8), 5 mM MgCl_2_, 60 mM KCl, 15 mM NaCl, 1 mM CaCl_2_, 1% (v/v) Triton X-100, 2.5% (v/v) Ficoll 400, 3.12 µL/mL mercaptoethanol, 1× protease inhibitor coctail). Chromatins were extracted with cold nuclei lysis buffer containing 50 mM Tris-HCl (pH 8), 10 mM EDTA, 1% (m/v) SDS after centrifugation at 4 °C × 3000 g for 20 min, and sonicated five times for 10 s at power 6. Chromatins were incubated with ChIP anti-GFP antibody (1:100, Invitrogen, MA5-15256) at 4 °C overnight. The coupled-chromatin fragments were pulled down by ChIP protein A + G magnetic beads (1:50, Magna, 16-663) at 4 °C for 4 h. The beads were washed for 5 min each time at 4 °C with 1 ml of each of the following buffers: 2 times with low salt wash buffer (150 mM NaCl, 20 mM Tris–HCl (pH 8), 0.2% (m/v) SDS, 0.5% (v/v) Triton X-100, and 2 mM EDTA), two times with high salt wash buffer (500 mM NaCl, 20 mM Tris–HCl (pH 8), 0.2% (m/v) SDS, 0.5% (v/v) Triton X-100, and 2 mM EDTA), two times with LiCl wash buffer (0.25 M LiCl, 1% (m/v) sodium deoxycholate, 10 mM Tris–HCl (pH 8), 1% (v/v) NP-40, and 1 mM EDTA), and two times with TE buffer (1 mM EDTA and 10 mM Tris–HCl pH 8). DNA was extracted from the beads with elution buffer containing 0.5% (m/v) SDS and 0.1 M NaHCO_3_ at 65 °C for 15 min and reversely cross-linked with 192 mM NaCl at 65 °C for overnight. Proteins were removed with equal volume of phenol/chloroform/isoamyl alcohol (25:24:1). DNA was precipitated with 2.5 volume of 100% ethanol, 1/10 volume of 3 M sodium acetate (pH 5.2), and dissolved to TE buffer containing 1 mM EDTA and 10 mM Tris–HCl (pH 8). qPCR analysis was used to detected the enrichment of the chromatin fragments. *35S*:*GFP* plants were used as the control. The promoter of *ACTIN7* was used as a negative control.

### Electrophoretic mobility shift assay

Electrophoretic mobility shift assay (EMSA) was performed according to the procedure of EMSA kit (Thermo). The CDSs of *MYC3* and *MYC4* were cloned into the *pMALC2*-*MBP* vector to generate *MBP*-*MYC3* and *MBP*-*MYC4* constructs, respectively. Proteins were expressed in *E. coli* BL21 (DE3) with 0.5 mM Isopropyl β-D-1-thiogalactopyranoside (IPTG) at 28 °C for 4 h. BL21 (DE3) cells were pelleted at 4 °C × 5000 g for 15 min and resuspended with the solution containing 50 mM HEPES (pH 7.5), 150 mM NaCl, 1 mM EGTA, 1% (v/v) Triton X-100, 10% (v/v) glycerol, and 1 mM PMSF. Proteins were obtained from bacteria after sonicating for 5 min (5 s on, 10 s stop) at 20 amplitude. The biotin-labelled and unlabelled probes were synthesised and incubated with MBP, MBP-MYC3, or MBP-MYC4 at room temperature for 20 min. Proteins were purified with anti-MBP agarose beads (NEB, E8037s). The interactions were detected with an anti-biotin antibody (1:3000, Invitrogen, 03-3720).

### Reporting summary

Further information on research design is available in the Nature Research Reporting Summary linked to this article.

## Supplementary information

Description of Additional Supplementary Files

Supplementary Information

Supplementary Data 1

Reporting Summary

## Data Availability

The RNA-seq data are available from the NCBI Sequence Read Archive under accession code PRJNA610584 [http://www.ncbi.nlm.nih.gov/bioproject/610584]. The source data underlying Figs. 1c–g, i–l, 3c–g, i–l, 4a, b, d, 5c–g, i–l, 7c–e, Supplementary Figs. 3a–d, 9a, b, 10a, b, 12 and 15b are provided in a Source Data file.

## Source data

Source Data
