# Supplementary material for: Transcriptional repression of GIF1 by the KIX-PPD-MYC repressor complex controls seed size in Arabidopsis
Source: Nat Commun. 2020 Apr 15;11:1846. doi: 10.1038/s41467-020-15603-3 (PMC7160150; doi:10.1038/s41467-020-15603-3)
Supplement: Supplementary file 1 — Description of Additional Supplementary Files [file 41467_2020_15603_MOESM1_ESM.pdf]

**Description of Additional Supplementary Files**

File Name: Supplementary Data 1
Description: Genes with significantly changed expression in both the first pair of leaves of myc3 myc4 and ppd1-2 ppd2-cr plants
