# Supplementary material for: Transcriptional repression of GIF1 by the KIX-PPD-MYC repressor complex controls seed size in Arabidopsis
Source: Nat Commun. 2020 Apr 15;11:1846. doi: 10.1038/s41467-020-15603-3 (PMC7160150; doi:10.1038/s41467-020-15603-3)
Supplement: Supplementary file 2 — Supplementary Information [file 41467_2020_15603_MOESM2_ESM.pdf]

Transcriptional repression of *GIF1* by the KIX-PPD-MYC repressor complex controls seed size in Arabidopsis

Liu et al.

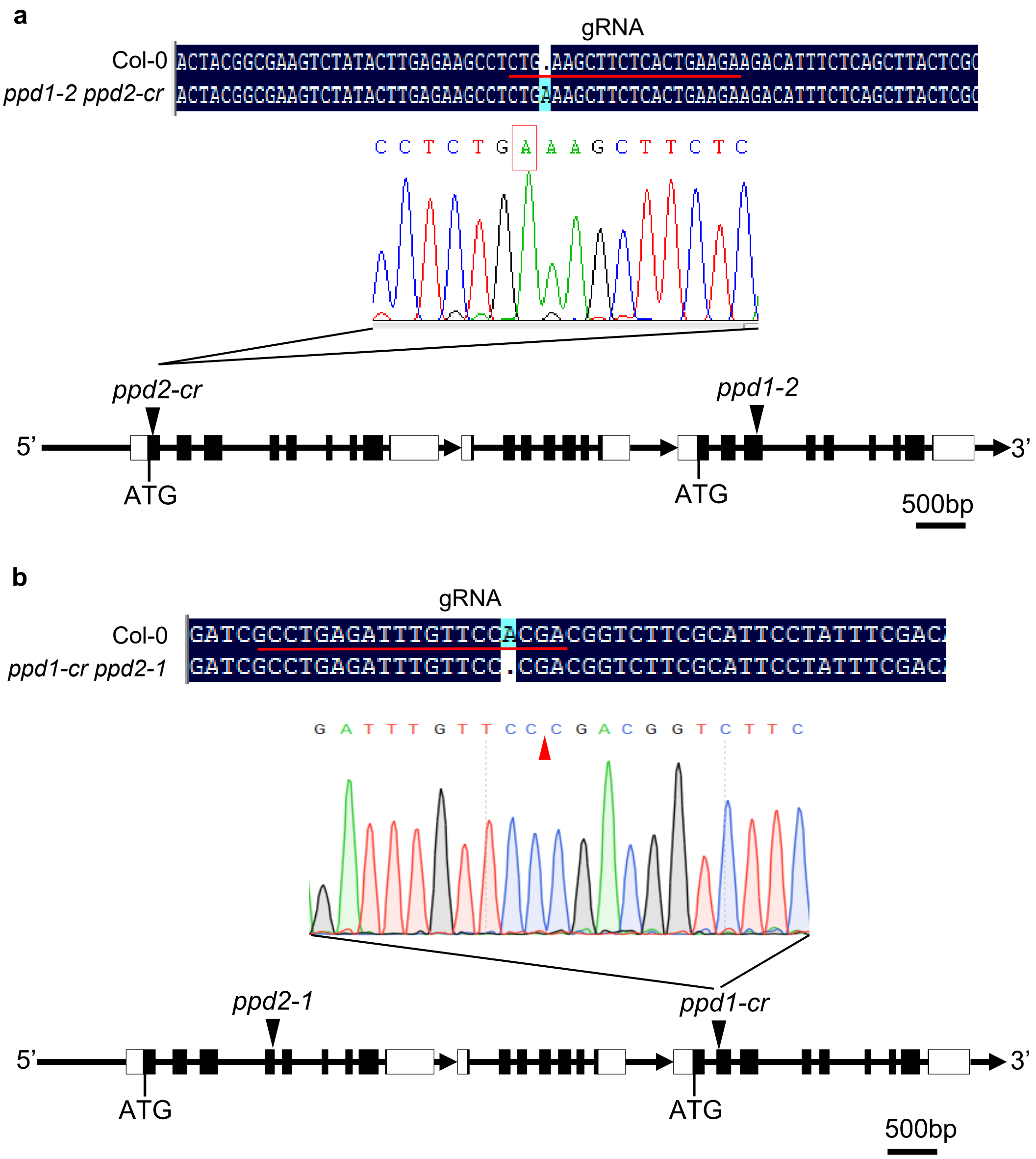

Supplementary Fig. 1 Identification of the *ppd1-2 ppd2-cr* and *ppd1-cr ppd2-1* mutants. **a** The *ppd1-2 ppd2-cr* plants were generated by CRISPR-Cas9 mediated genome editing in the *ppd1-2* background. Alignment analysis of Col-0 and *ppd1-2 ppd2-cr* genomic DNA (top). The gRNA with red line is used for CRISPR-Cas9 mediated genome editing. Sequencing chromatogram of the genomic DNA from *ppd1-2 ppd2-cr* plants (middle). The A base with a red box represents the insertion base and causes a frameshift mutation of *PPD2.* The mutation sites of *ppd1-2* and *ppd2-cr* in the *ppd1-2 ppd2-cr* plants are showed (bottom). **b** The *ppd1-cr ppd2-1* plants were generated by CRISPR-Cas9 mediated genome editing in the *ppd2-1* background. Alignment analysis of Col-0 and *ppd1-cr ppd2-1* genomic DNA (top). The gRNA with red line is used for CRISPR-Cas9 mediated genome editing. Sequencing chromatogram of the genomic DNA from *ppd1-cr ppd2-1* plants (middle). The red arrow position represents where the missing A base should be in.The mutation sites of *ppd1-cr* and *ppd2-1* in the *ppd1-cr ppd2-1* plants are showed (bottom).

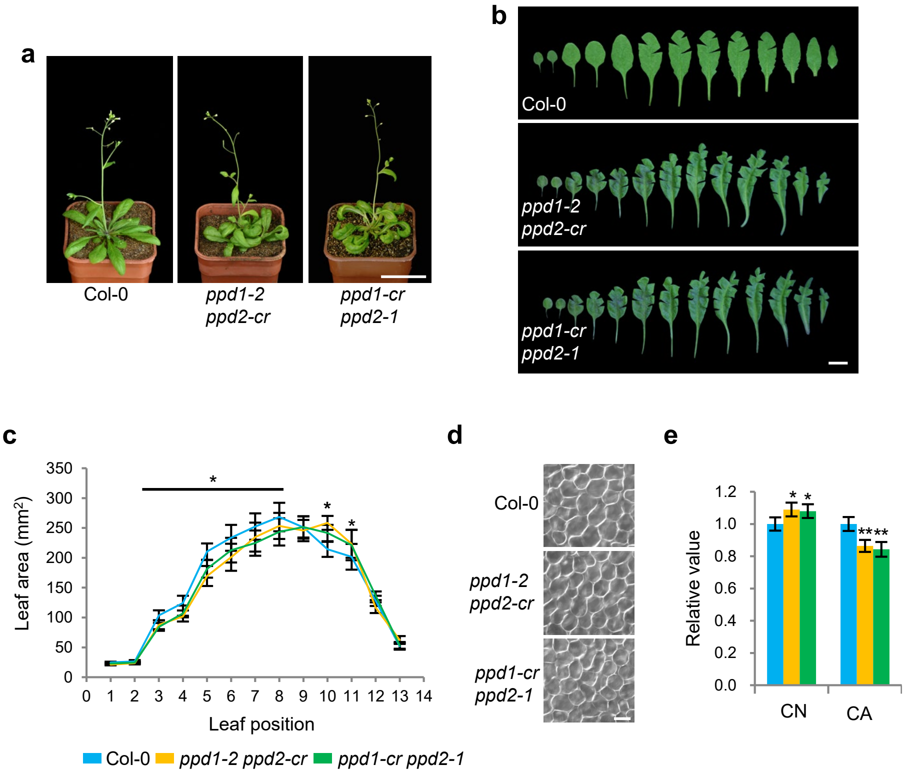

Supplementary Fig. 2 The phenotypes of *ppd1-2 ppd2-cr* and *ppd1-cr ppd2-1* plants. **a** 32-day-old plants of Col-0, *ppd1-2 ppd2-cr*, and *ppd1-cr ppd2-1* plants. **b** Leaf series of Col-0, *ppd1-2 ppd2-cr,* and *ppd1-cr ppd2-1*. **c** Area of individual leaves from Col-0, *ppd1-2 ppd2-cr,* and *ppd1-cr* (n = 12). **d** Palisade cells in the fifth leaf of Col-0, *ppd1-2 ppd2-cr*, and *ppd1-cr ppd2-1*. **e** The palisade cell number (CN, n = 8) and palisade cell area (CA, n = 300) in the fifth leaf of Col-0, *ppd1-2 ppd2-cr*, and *ppd1-cr ppd2-1*. Scale bars, 4 cm (**a**), 1 cm (**b**), and 50 μm (**d**). Error bars represent ±SE. *, significant difference from the Col-0, one-way ANOVA *P*-values: * $P < 0.05$ and ** $P < 0.01$.

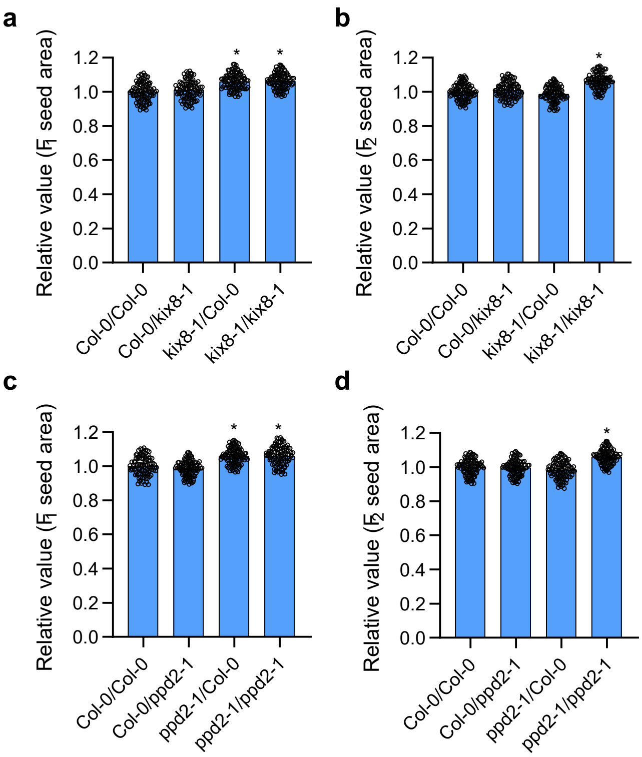

Supplementary Fig. 3 KIX8 and PPD2 act maternally to control seed development. **a-b** The relative area of F_1 seeds (**a**) and F_2 seeds (**b**) from Col-0/Col-0, Col-0/*kix8-1*, *kix8-1*/Col-0, and *kix8-1*/*kix8-1* plants (n = 100). **c-d** The relative area of F_1 seeds (**c**) and F_2 seeds (**d**) from Col-0/Col-0, Col-0/*ppd2-1*, *ppd2-1*/Col-0, and *ppd2-1*/*ppd2-1* plants (n = 100). Seeds from the third to seventh silique on the stem of six plants were used for analysis. Error bars represent ±SE. *, significant difference from the Col-0, one-way ANOVA *P*-values: * $P < 0.05$.

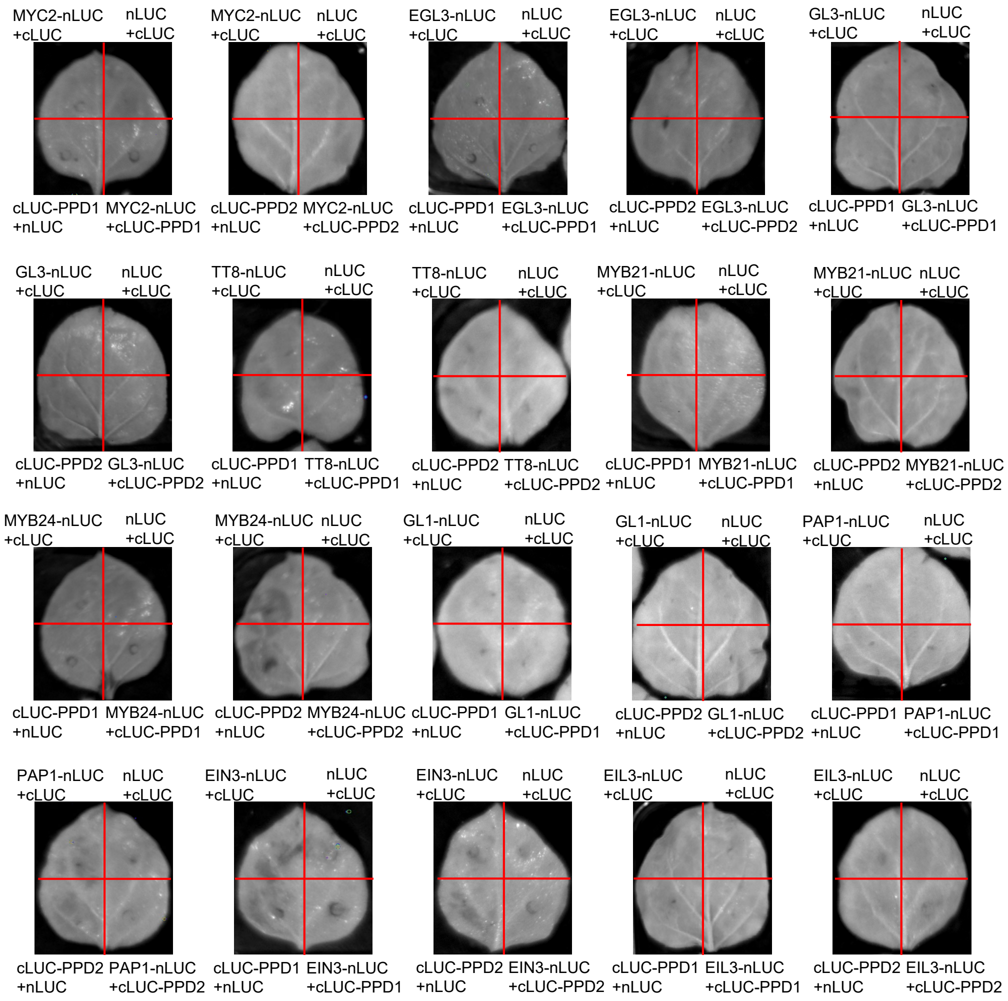

Supplementary Fig. 4 The PPD1/2-interacting proteins finding by split luciferase complementation assays.

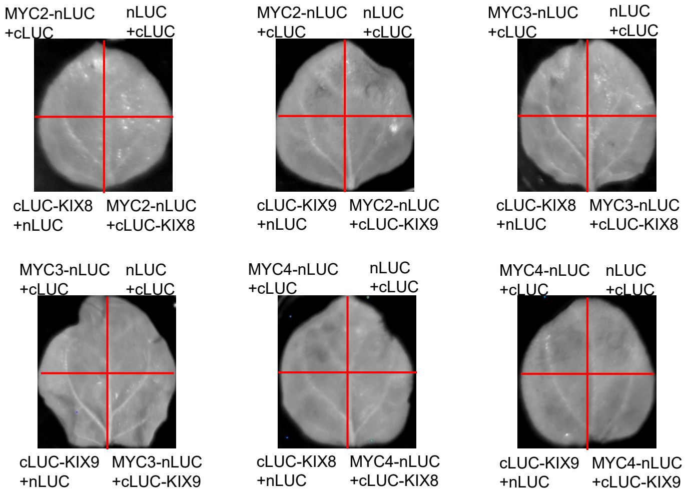

Supplementary Fig. 5 KIX8 and KIX9 are not interact with MYC2/3/4 by split luciferase complementation assays .

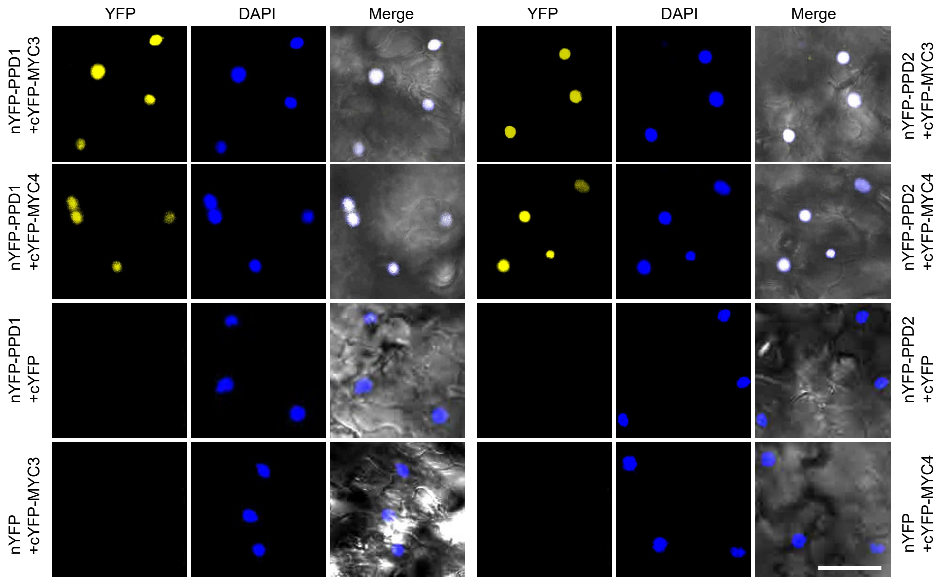

Supplementary Fig. 6 The bimolecular fluorescence complementation assays showing that PPD1/2 interact with MYC3/4 in *N. benthamiana* leaves. YFP fluorescence was observed at 2 days later after coinfiltrating with different combinations of *nYFP-PPD1/2*, *cYFP-MYC3/4*, *nYFP*, and *cYFP* constructs. Blue dots represent the nuclei after staining with DAPI . Scale bar = 50 μm.

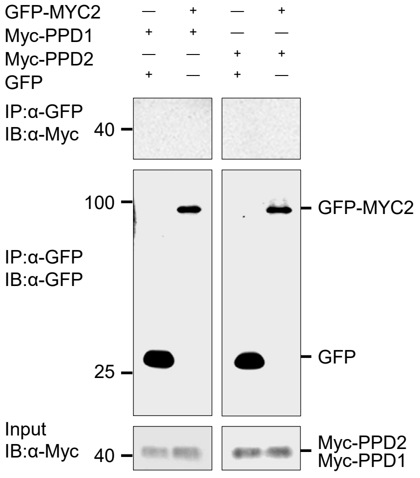

Supplementary Fig. 7 PPD1 and PPD2 are not interact with MYC2 in Arabidopsis by co-immunoprecipitation analyses. Total protein extracts of *35S:Myc-PPD1/2*;*35S*:*GFP* and *35S:Myc-PPD1/2*;*35S:GFP-MYC2* plants were incubated with GFP-Trap-A agarose beads. Precipitates were detected by Western blot with anti-GFP or anti-Myc antibody.

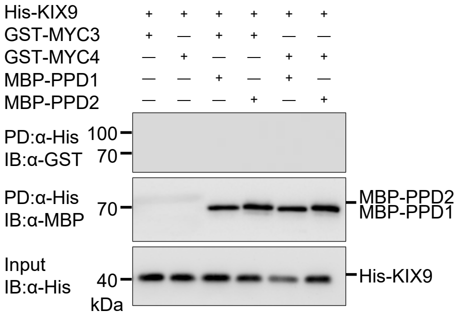

Supplementary Fig. 8 KIX9 is not interact with MYC3/4 by pull-down analyses. His-KIX9 was incubated with GST-MYC3 or GST-MYC4 and MBP-PPD1 or MBP-PPD2. Proteins were pulled down by the Ni-NTA agarose beads and detected by Western blot with anti-GST, anti-MBP, or anti-His antibody.

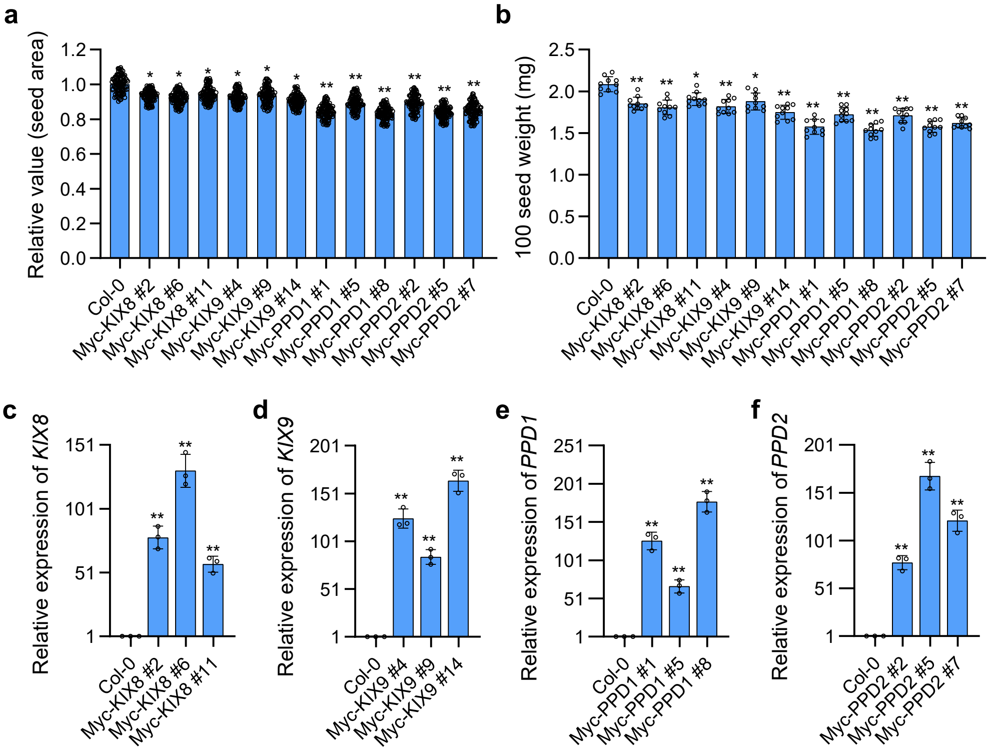

Supplementary Fig. 9 The seed phenotype of the plants with overexpression of *KIX8*, *KIX9*, *PPD1*, and *PPD2*. **a-b** Seed area (**a**, n = 100), and 100 seed weight (**b**, n = 10) of Col-0, *35S*:*Myc-KIX*8 (Myc-KIX8 #2, #6, #11), *35S*:*Myc-KIX*9 (Myc-KIX9 #4, #9, #14), *35S*:*Myc-PPD1* (Myc-PPD1 #1, #5, #8), and *35S*:*Myc-PPD2* (Myc-PPD2 #2, #5, #7) plants. **c** The relative expression levels of *KIX8* in the 2 DAF (days after flowering) siliques of Col-0, and *35S*:*Myc-KIX*8 (Myc-KIX8 #2, #6, #11) (n = 3). **d** The relative expression levels of *KIX9* in the 2 DAF siliques of Col-0, and *35S*:*Myc-KIX*9 (Myc-KIX9 #4, #9, #14) . **e** The relative expression levels of *PPD1* in the 2 DAF siliques of Col-0, and *35S*:*Myc-PPD1* (Myc-PPD1 #1, #5, #8) (n = 3). **f** The relative expression levels of *PPD2* in the 2 DAF siliques of Col-0, and *35S*:*Myc-PPD2* (Myc-PPD2 #2, #5, #7) (n = 3). Seeds from the third to seventh silique on the stem of six plants were used for analysis. Scale bars = 0.5 mm. Error bars represent ±SE. *, significant difference from the Col-0, one-way ANOVA *P*-values: * $P < 0.05$ and ** $P < 0.01$.

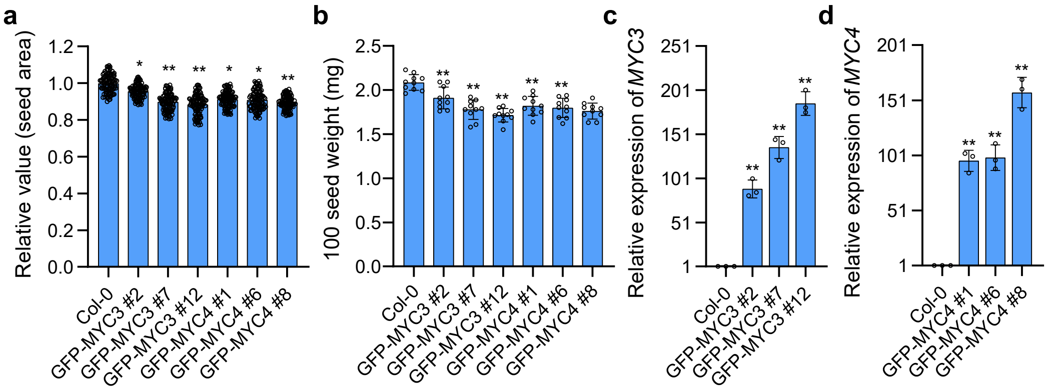

Supplementary Fig. 10 The seed phenotype of the plants with overexpression of *MYC3* and *MYC4*. **a-b** Seed area (**a**, n = 100), and 100 seed weight (**b**, n = 10) of Col-0, *35S*:*GFP-MYC3* (GFP-MYC3 #2, #7, #12), and *35S*:*GFP-MYC4* (GFP-MYC4 #1, #6, #8) plants. **c** The relative expression levels of *MYC3* in the 2 DAF (days after flowering) siliques of Col-0, and *35S*:*GFP-MYC3* (GFP-MYC3 #2, #7, #12) (n = 3). **d** The relative expression levels of *MYC4* in the 2 DAF siliques of Col-0, and *35S*:*GFP-MYC4* (GFP-MYC4 #1, #6, #8) (n = 3). Seeds from the third to seventh silique on the stem of six plants were used for analysis. Scale bars = 0.5 mm. Error bars represent ±SE. *, significant difference from the Col-0, one-way ANOVA *P*-values: * $P < 0.05$ and ** $P < 0.01$.

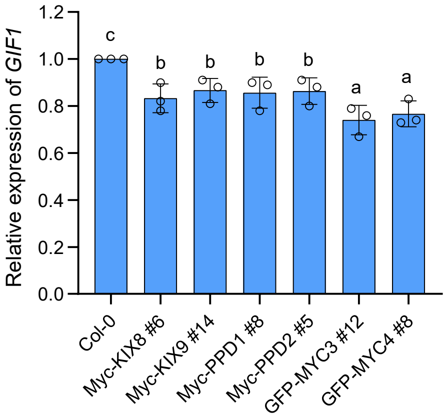

Supplementary Fig. 11 The relative expression levels of *GIF1* in the 2 DAF siliques of *35S*:*Myc-KIX*8 #6, *35S*:*Myc-KIX*9 #14, *35S*:*Myc-PPD1* #8, *35S*:*Myc-PPD2* #5, *35S*:*GFP-MYC3* #12, and *35S*:*GFP-MYC4* #8 were detected by qPCR (n = 3). Data was normalized with *ACTIN2.* Error bars represent ±SE. Different lowercase letters above the columns indicate the significant difference among different groups, one-way ANOVA *P*-values: $P < 0.05$.

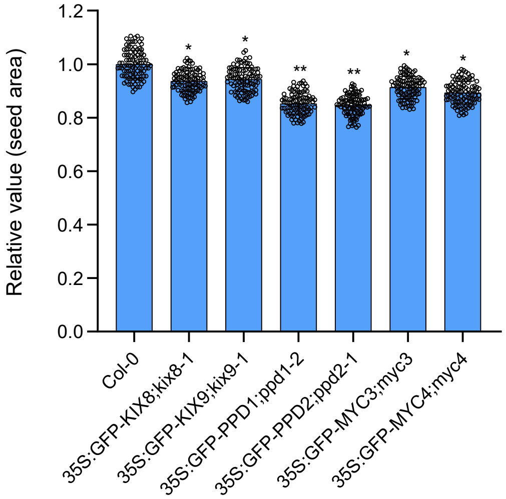

Supplementary Fig. 12 The seed phenotype of *35S*:*GFP-KIX8*;*kix8-1*, *35S*:*GFP-KIX9*;*kix9-1*, *35S*:*GFP-PPD1*;*ppd1-2*, *35S*:*GFP-PPD2*;*ppd2-1*, *35S*:*GFP-MYC3*;*myc3*, and *35S*:*GFP-MYC4*;*myc4* plants. Seed area (n = 100) of *35S*:*GFP-KIX8*;*kix8-1*, *35S*:*GFP-KIX9*;*kix9-1*, *35S*:*GFP-PPD1*;*ppd1-2*, *35S*:*GFP-PPD2*;*ppd2-1*, *35S*:*GFP-MYC3*;*myc3*, and *35S*:*GFP-MYC4*;*myc4* plants. Seeds from the third to seventh silique on the stem of six plants were used for analysis. Scale bars = 0.5 mm. Error bars represent ±SE. *, significant difference from the Col-0, one-way ANOVA *P*-values: * $P < 0.05$ and ** $P < 0.01$.

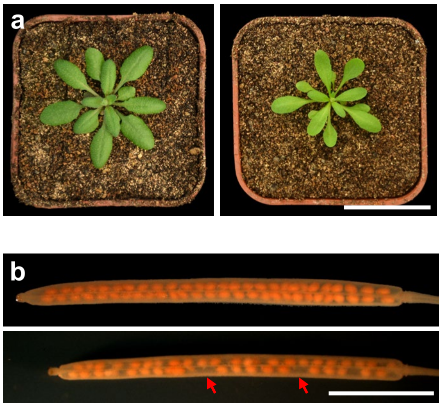

Supplementary Fig. 13 The phenotypes of *gif1* plants. **a** The 25-day-old plants of Col-0 and *gif1* (from left to right). **b** The 10-DAP-old siliques of Col-0 and *gif1* (from up to down). Red arrows show the abortion sites. Scale bars, 4 cm (**a**) and 0.5 cm (**b**).

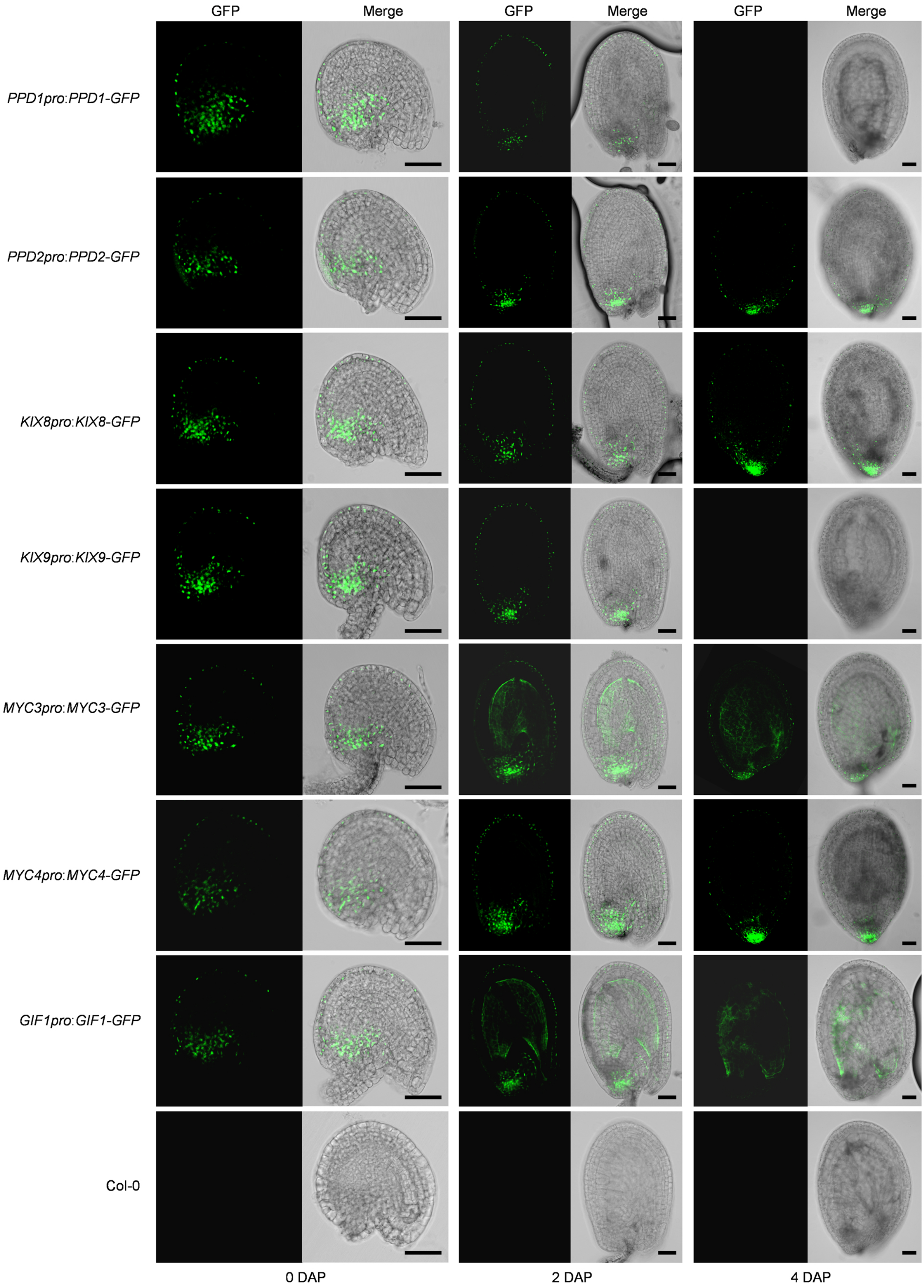

Supplementary Fig. 14 Expression patterns of *KIX8*, *KIX9*, *PPD1*, *PPD2*, *MYC3*, *MYC4*, and *GIF1* in seeds during seed development. The promotors of *KIX8* (2,087 bp), *KIX9* (1,714 bp), *PPD1* (1,797 bp), *PPD2* (2,153 bp), *MYC3* (2,180 bp), *MYC4* (2,132 bp), and *GIF1* (2,337 bp) and their CDSs were cloned into the *pMDC107-GFP* vector to generate *KIX8pro*:*KIX8-GFP*, *KIX9pro*:*KIX9-GFP*, *PPD1pro*:*PPD1-GFP, PPD2pro*:*PPD2-GFP*, *MYC3pro*:*MYC3-GFP*, *MYC4pro*:*MYC4-GFP*, and *GIF1pro*:*GIF1-GFP* constructs, respectively. GFP fluorescence was observed by the confocal microscopy. DAP, days after pollination. Scale bars = 50 μm.

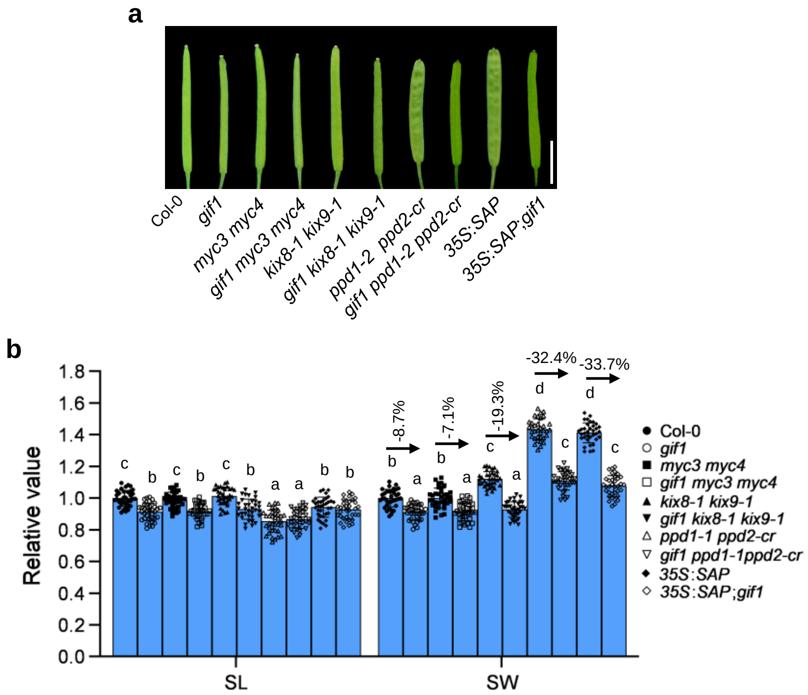

Supplementary Fig. 15 The siliques (**a**) and silique length (SL) and silique width (SW) (**b**) of Col-0, *gif1*, *myc3 myc4*, *gif1 myc3 myc4*, *kix8-1 kix9-1*, *gif1 kix8-1 kix9-1*, *ppd1-2 ppd2-cr*, *gif1 ppd1-2 ppd2-cr*, *35S*:*SAP*, and *35S*:*SAP*;*gif1* plants (n = 35). Siliques from the fourth silique on the stem of plants were used for analysis. Scale bar = 0.5 cm. Error bars represent ±SE. Different lowercase letters above the columns indicate the significant difference among different groups, one-way ANOVA *P*-values: $P < 0.05$.

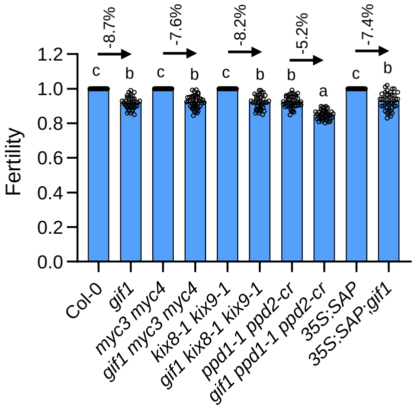

Supplementary Fig. 16 The fertility of Col-0, *gif1*, *myc3 myc4*, *gif1 myc3 myc4*, *kix8-1 kix9-1*, *gif1 kix8-1 kix9-1*, *ppd1-2 ppd2-cr*, *gif1 ppd1-2 ppd2-cr*, *35S*:*SAP*, and *35S*:*SAP*;*gif1* plants (n = 40). Error bars represent ±SE. Different lowercase letters above the columns indicate the significant difference among different groups, one-way ANOVA *P*-values: $P < 0.05$.

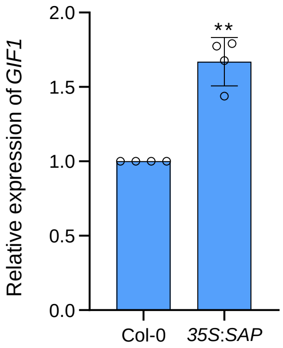

Supplementary Fig. 17 The *GIF1* expression in the *35S*:*SAP* plants. The relative expression levels of *GIF1* in the 3 DAP siliques of Col-0, and *35S:SAP* were detected by qPCR (n = 4). Data are normalized with *ACTIN2.* Error bars represent ±SE. *, significant difference from the Col-0, one-way ANOVA *P*-values: ** *P* < 0.01.

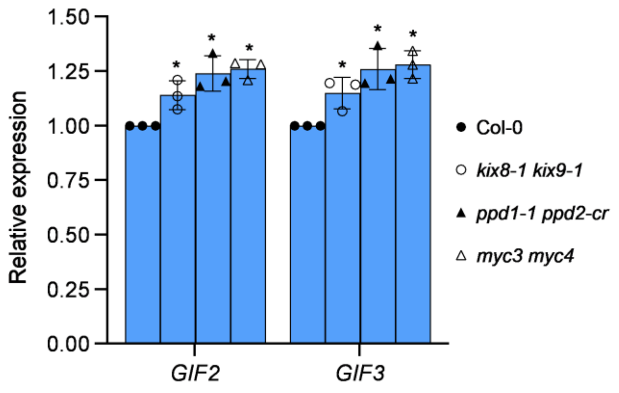

Supplementary Fig. 18 The expression levels of *GIF2* and *GIF3* in the Col-0, *kix8-1 kix9-1*, *ppd1-2 ppd2-cr*, and *myc3 myc4*. The expression levels of *GIF2* and *GIF3* in 3 DAP siliques of the Col-0, *kix8-1 kix9-1*, *ppd1-2 ppd2-cr*, and *myc3 myc4* were detected by qPCR (n = 3). Data was normalized with *ACTIN2.* Error bars represent ±SE. *, significant difference from the Col-0, one-way ANOVA *P*-values: * $P < 0.05$.

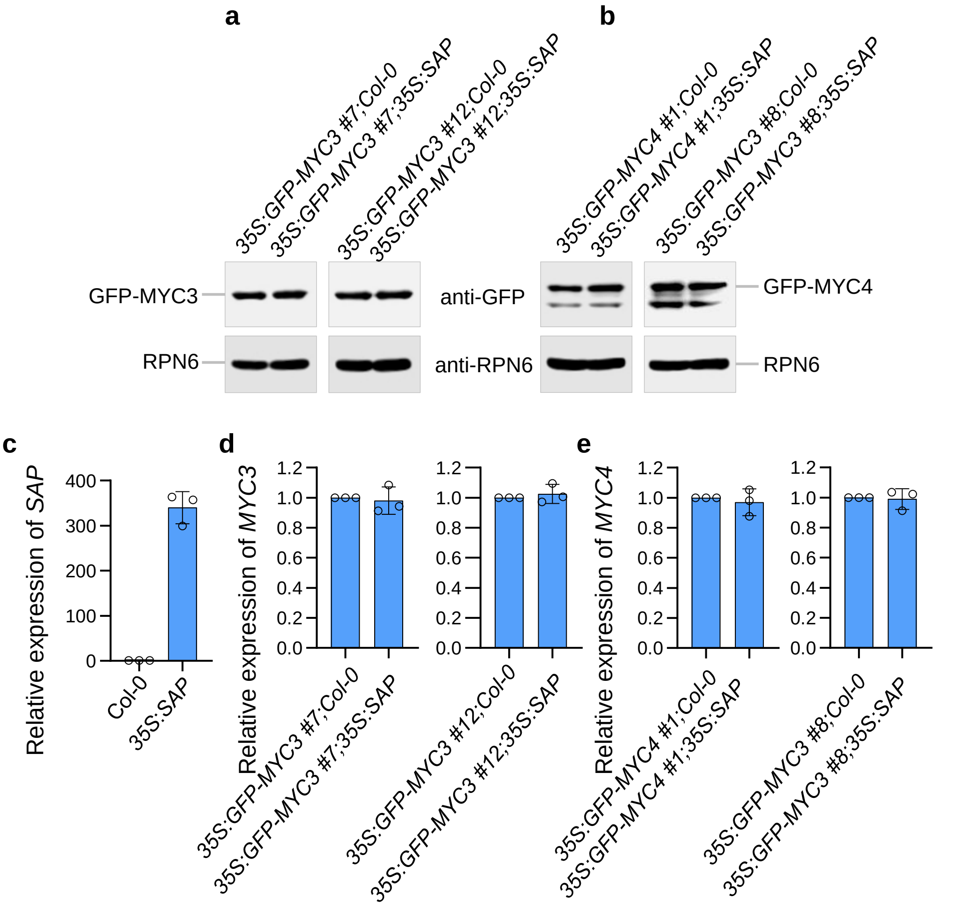

Supplementary Fig. 19 The stabilities of MYC3 and MYC4 are not regulated by SAP. **a-b** The amounts of GFP-MYC3 and GFP-MYC4 in the plants with SAP overexpression. *35S:GFP-MYC3* (#7 and #12) or *35S:GFP-MYC4* (#1 and #8) transgenic line was crossed with Col-0 and *35S:SAP* plants to generate *35S:GFP-MYC3 #7;*Col-0, *35S:GFP-MYC3 #7*;35S:SAP, *35S:GFP-MYC3 #12;*Col-0, *35S:GFP-MYC3 #12*;*35S:SAP*, *35S:GFP-MYC4 #1;*Col-0, *35S:GFP-MYC4 #1;35S:SAP*, *35S:GFP-MYC3 #8;*Col-0, and *35S:GFP-MYC3 #8*;*35S:SAP*, respectively. 10-day-old F_1 seedlings were used for analysis with anti-GFP and anti-RPN6 antibodies. RPN6 was used as loading controls. **c** The relative expression of *SAP* in *35S:SAP* seedlings (n = 3). 10-day-old seedlings were used for analysis by qPCR. Data was normalized with *ACTIN2.* Error bars represent ±SE. **d** The relative expression of *MYC3* in *35S:GFP-MYC3 #7;*Col-0, *35S:GFP-MYC3 #7*;35S:SAP, *35S:GFP-MYC3 #12;*Col-0, and *35S:GFP-MYC3 #12*;*35S:SAP* seedlings (n = 3). 10-day-old F_1 seedlings were used for analysis by qPCR. Data was normalized with *ACTIN2.* Error bars represent ±SE. **e** The relative expression of *MYC4* in *35S:GFP-MYC4 #1;*Col-0, *35S:GFP-MYC4 #1;35S:SAP*, *35S:GFP-MYC3 #8;*Col-0, and *35S:GFP-MYC3 #8*;*35S:SAP* seedlings (n = 3). 10-day-old F_1 seedlings were used for analysis by qPCR. Data was normalized with *ACTIN2.* Error bars represent ±SE.

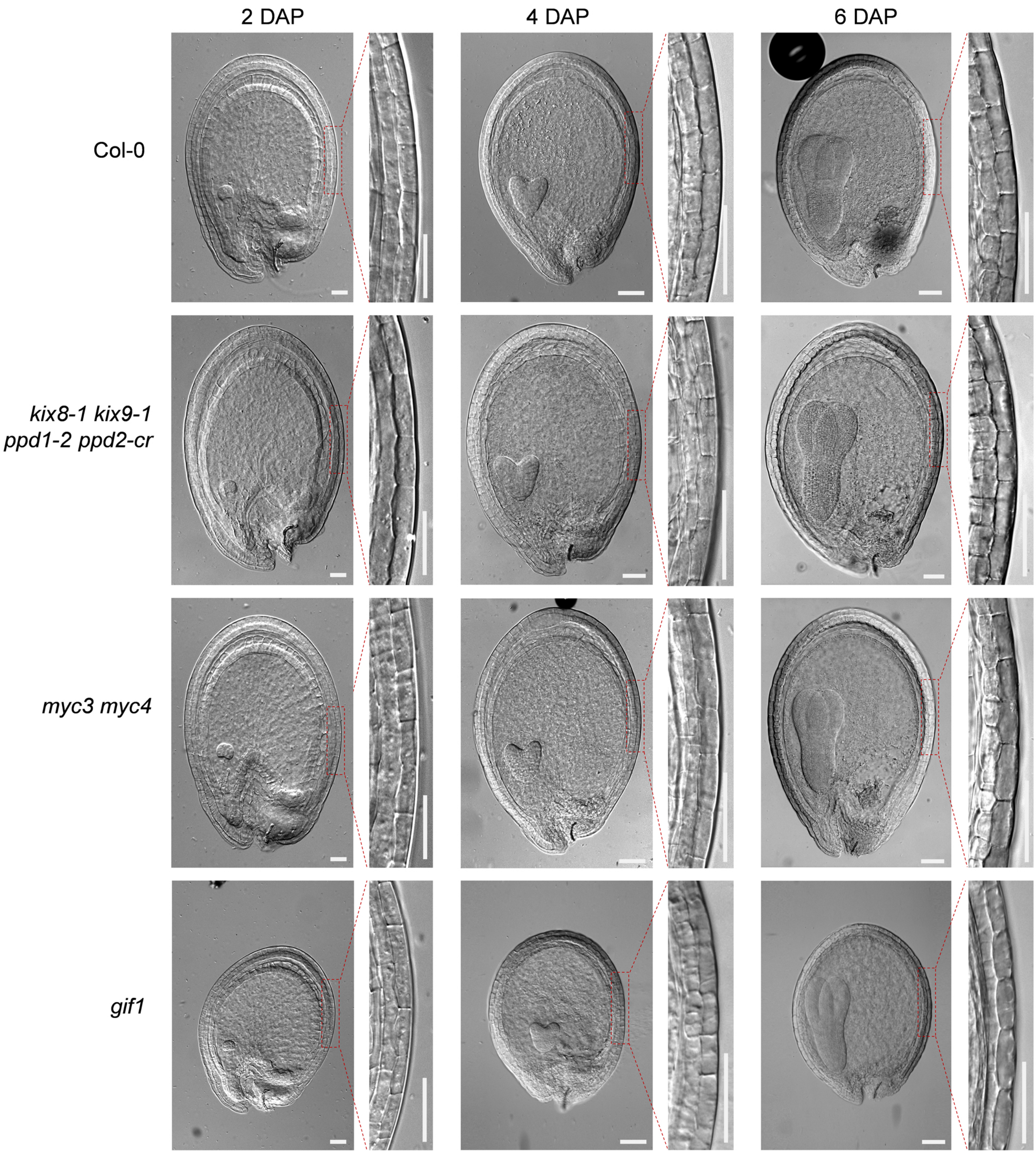

Supplementary Fig. 20 The seeds of Col-0, *kix8-1 kix9-1 ppd1-2 ppd2-cr*, *myc3 myc4*, and *gif1* at 2, 4 and 6 DAP. The seeds were obtained under 20x magnification by the differential interference contrast microscope. The outer integument cells were obtained under 40x magnification by the differential interference contrast microscope. Scale bars, 25 μm (images at 2 DAP) and 50 μm (images at 4 and 6 DAP). Supports for Fig. 1i-l, Fig. 3i-l and Fig. 5i-l.

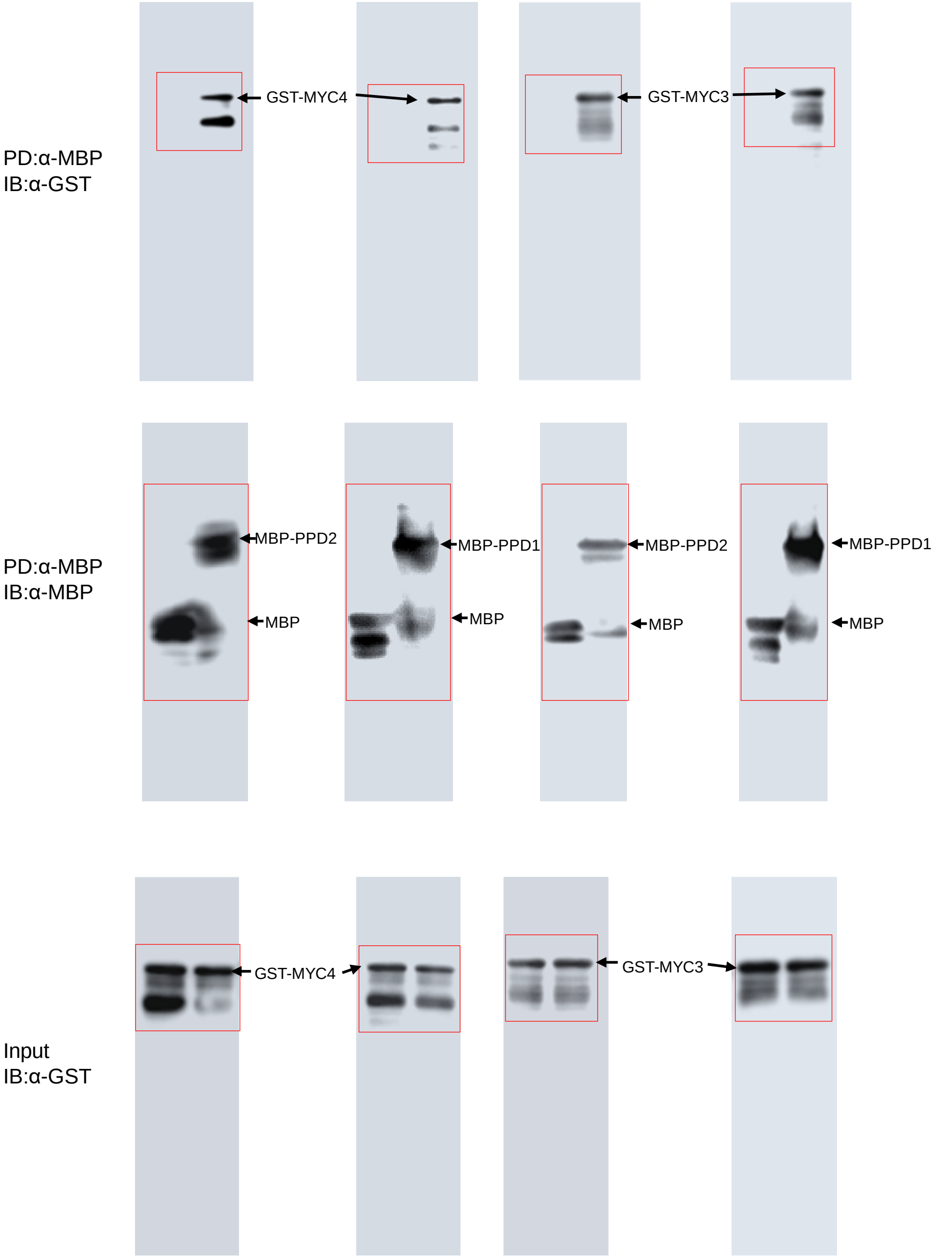

Supplementary Fig. 21 Original images of Fig. 2c.

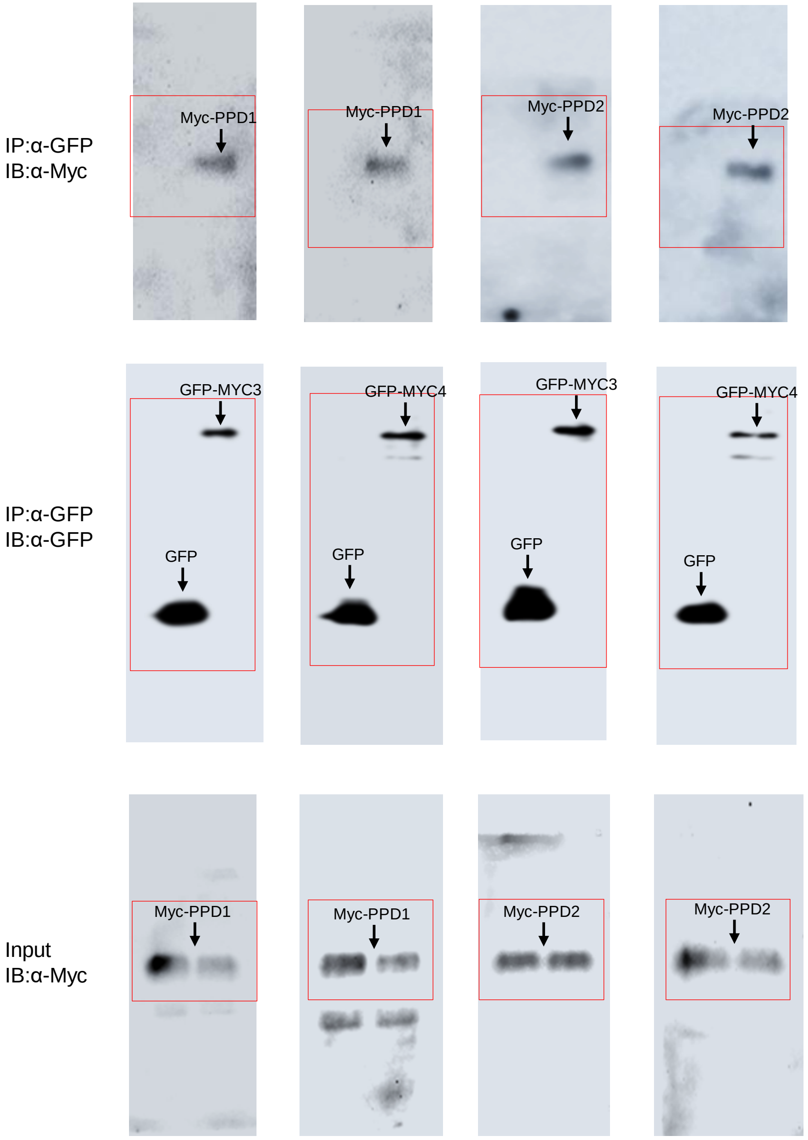

Supplementary Fig. 22 Original images of Fig. 2d.

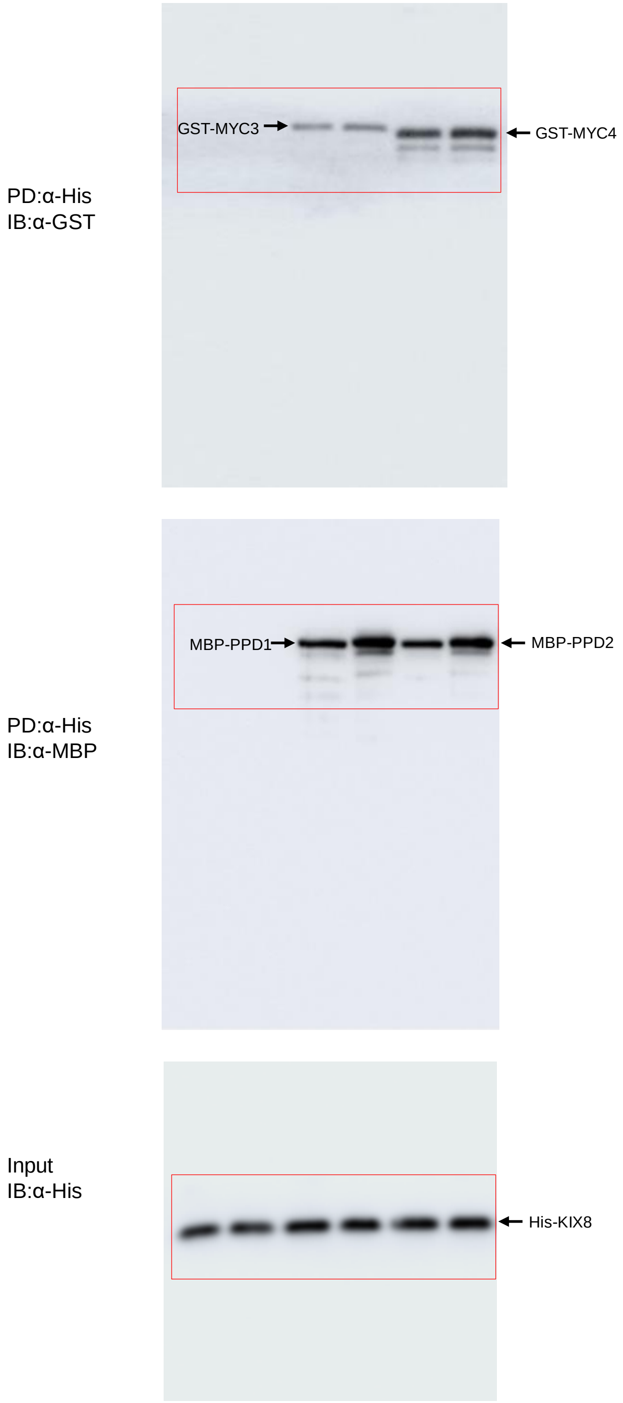

Supplementary Fig. 23 Original images of Fig. 2e**.**

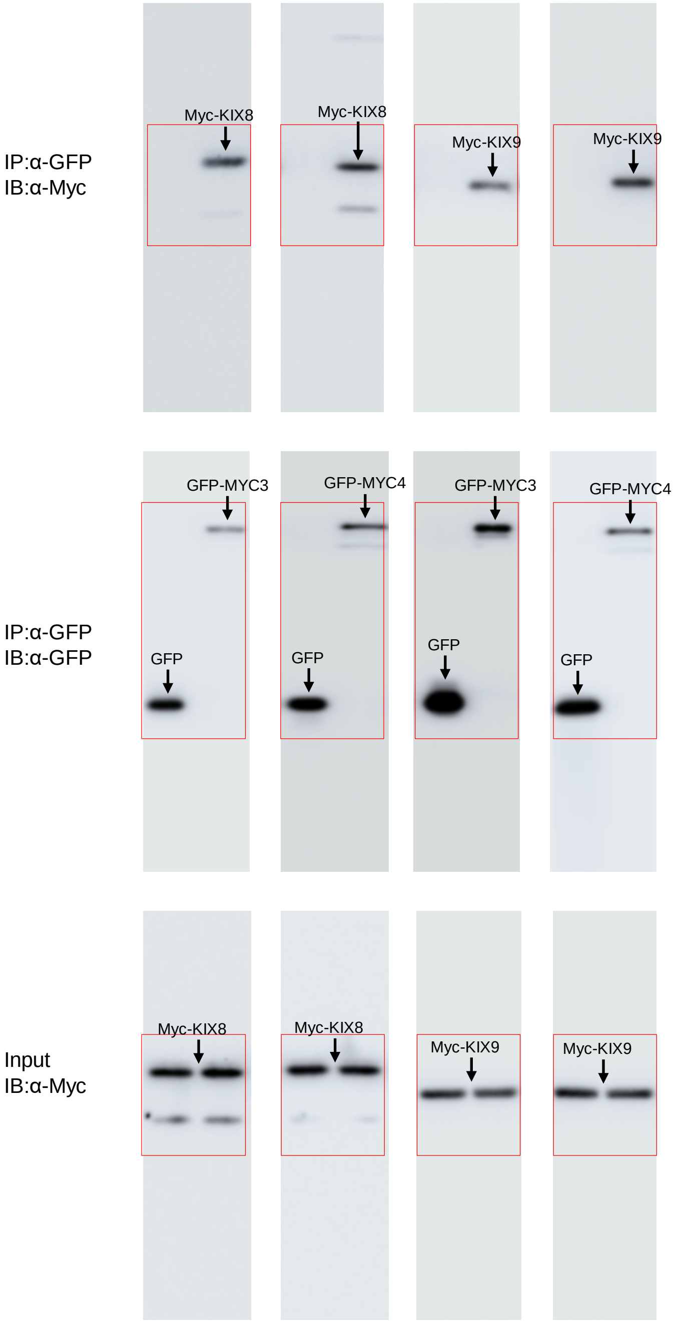

Supplementary Fig. 24 Original images of Fig. 2f.

Supplementary Table 1 | List of primers used in this study

Primer Name	Sequence	Purpose
ppd1-2F	ACGAGAAGAATTCGCCTTCTC	genotype analysis
ppd1-2R	TGGCCACAAGACGACTATTTC	genotype analysis
ppd2-1F	CGCTCTCAGGTGTTTTAAAGC	genotype analysis
ppd2-1R	GAATCATGGTTTTGATGGTGG	genotype analysis
kix8-1F	TTGGTGCCACTTAGACCAAAC	genotype analysis
kix8-1R	TGAAAATCTGGCGAATAATCG	genotype analysis
kix9-1F	ATTTGACGCAAGTTATCCACG	genotype analysis
kix9-1R	CTTGAAGCTCTCCTTGGTGTG	genotype analysis
gif1-F	AGGGTCCAGTTGTGTTTTGAGTTC	genotype analysis
gif1-R	GCTCGAGTCCTTGTGCCACACTCTT	genotype analysis
myc3-F	AGGCAAAACCCATTTACAACC	genotype analysis
myc3-R	TGAAGCAGAGAGGCAGAGAAG	genotype analysis
myc4-F	CTCCTTGACAAATTTGATCCG	genotype analysis
myc4-R	CGCTACACACACCATTGTTTG	genotype analysis
LBa1	TGGTTCACGTAGTGGGCCATCG	genotype analysis
8474	ATAATAACGCTGCGGACATCTACATT	genotype analysis
LB1	CAGAAATGGATAAATAGCCTTG	genotype analysis
LBb1.3	ATTTTGCCGATTTCGGAAC	genotype analysis
0849	ATTGACCATCATACTCATTGC	genotype analysis
PPD1-CRIJD-F	GGATTCCGGTGAAGAGGAGG	genotype analysis
PPD1-CRIJD-R	TCAATCAACGTTGTTGTGAC	genotype analysis
PPD2-CRIJD-F	AATCTCTGTTTTCTTTCGTGTC	genotype analysis
PPD2-CRIJD-R	AGATAAAACTTGCTGGATCGC	genotype analysis
GIF1-QF	CTACCCCAGCAATGTTACCTC	qPCR
GIF1-QR	ATTCGCTAAGCTTTCCAGAGT	qPCR
GIF2-QF	TACAGTTTGGTAGCCCACTCC	qPCR
GIF2-QR	TGGTCTAATCCCCATGTGTCC	qPCR
GIF3-QF	TTCCTTCATTTCCGCCCAC	qPCR
GIF3-QR	CTGATTTTCCAAGATCGCCAT	qPCR
V159-MYC3F	GGGACGAGCTCGGTACCCGGGGATCCATGAACGGCACAACA TCATCAATC	*MYC3-nLUC*
V159-MYC3R	GGGACGCGTACGAGATCTGGTCGACATAGTTTTCTCCGACTT TC	*MYC3-nLUC*
V45-MYC3F	GGTTCCGCGTGGATCCCCGGAATTCATGAACGGCACAACAT CATCAATC	*GST-MYC3*
V45-MYC3R	GCCGCTCGAGTCGACCCGGGAATTCTCAATAGTTTTCTCCGA CTTTC	*GST-MYC3*
YC-MYC3F	CTTACGATGTTCCTGACTATGCGATGAACGGCACAACATCAT CAATC	*cYFP-MYC3*
YC-MYC3R	AACATATCCAGTCACTATGGTCAATAGTTTTCTCCGACTTTC	*cYFP-MYC3*

GW-MYC3F	GGGGACAAGTTTGTACAAAAAAGCAGGCTTCATGAACGGCACAACATCATCAATC	*GFP- MYC3*
GW-MYC3R	GGGGACCACTTTGTACAAGAAAGCTGGGTCTCAATAGTTTTCTCCGACTTTC	*GFP- MYC3*
GW-MYC4F	GGGGACAAGTTTGTACAAAAAAGCAGGCTTCATGTCTCCGACGAATGTTCAAG	*GFP- MYC4*
GW-MYC4R	GGGGACCACTTTGTACAAGAAAGCTGGGTCTCATGGACATTCTCCAACTTTC	*GFP- MYC4*
V159-MYC4F	GGGACGAGCTCGGTACCCGGGGATCCATGTCTCCGACGAATGTTCAAG	*MYC4-nLUC*
V159-MYC4R	GGGACGCGTACGAGATCTGGTCGACTGGACATTCTCCAACTTTC	*MYC4-nLUC*
V45-MYC4F	GGTTCCGCGTGGATCCCCGGAATTCATGTCTCCGACGAATGTTCAAG	*GST-MYC4*
V45-MYC4R	GCCGCTCGAGTCGACCCGGGAATTCTCATGGACATTCTCCAACTTTC	*GST-MYC4*
YC-MYC4F	CTTACGATGTTCCTGACTATGCGATGTCTCCGACGAATGTTCAAG	*cYFP-MYC4*
YC-MYC4R	AACATATCCAGTCACTATGGTCATGGACATTCTCCAACTTTC	*cYFP-MYC4*
V158-PPD1F	GTCCCGGGGCGGTACCCGGGGATCCATGGATGTCGGAGTTTCACC	*cLUC-PPD1*
V158-PPD1R	TCCTTGTAGTCCATTTGTTGGATCCTTAAATGCCTTCACTGTTTAGA	*cLUC-PPD1*
V158-PPD2F	GTCCCGGGGCGGTACCCGGGGATCCATGGATGTAGGAGTTACTACGG	*cLUC-PPD2*
V158-PPD2R	TCCTTGTAGTCCATTTGTTGGATCCTTAATTATCTTCGCTGTTTAGATCA	*cLUC-PPD2*
MYC3-F2	CTAGAGGATCCCCGGGTACCATGAACGGCACAACATCATC	*MYC3-CFP*
MYC3-R2	GCGGCCGCTCTAGAACTAGTATAGTTTTCTCCGACTTTCG	*MYC3-CFP*
MYC4-F2	CTAGAGGATCCCCGGGTACCATGTCTCCGACGAATGTTC	*MYC4-CFP*
MYC4-R2	GCGGCCGCTCTAGAACTAGTTGGACATTCTCCAACTTTC	*MYC4-CFP*
PPD1-F2	CTAGAGGATCCCCGGGTACCATGGATGTCGGAGTTTCACC	*PPD1-YFP*
PPD1-R2	GCGGCCGCTCTAGAACTAGTAATGCCTTCACTGTTTAGATC	*PPD1-YFP*
PPD2-F2	CTAGAGGATCCCCGGGTACC ATGGATGTAGGAGTTACTAC	*PPD2-YFP*
PPD2-R2	GCGGCCGCTCTAGAACTAGTATTATCTTCGCTGTTTAGATC	*PPD2-YFP*
DEL1-F2	CTAGAGGATCCCCGGGTACCATGTCAGATCTATCGCCAG	*DEL1-YFP*
DEL1-R2	GCGGCCGCTCTAGAACTAGTACGGTGTTGTGATGTATTAG	*DEL1-YFP*
V47-PPD1F	GGGAAGGATTTCAGAATTCGGATCCATGGATGTCGGAGTTTCACC	*MBP-PPD1*
V47-PPD1R	CCTGCAGGTCGACTCTAGAGGATCCTTAAATGCCTTCACTGTTTAGAT	*MBP-PPD1*
V47-PPD2F	GGGAAGGATTTCAGAATTCGGATCCATGGATGTAGGAGTTACTACGG	*MBP-PPD2*

V47-PPD2R	CCTGCAGGTCGACTCTAGAGGATCCTTAATTATCTTCGCTGTTTAGATC	*MBP-PPD2*
YN-PPD1F	GAGGAAGAGTATATGCCTATGGAAATGGATGTCGGAGTTTCACC	*nYFP-PPD1*
YN-PPD1R	AACATATCCAGTCACTATGGTTAAATGCCTTCACTGTTTAGAT	*nYFP-PPD1*
YN-PPD2F	GAGGAAGAGTATATGCCTATGGAAATGGATGTAGGAGTTACTACGG	*nYFP-PPD2*
YN-PPD2R	AACATATCCAGTCACTATGGTTAATTATCTTCGCTGTTTAGATC	*nYFP-PPD2*
V6-PPD1F	GAGGACTTGAATTCGGTACCCATGGATGTCGGAGTTTCACC	*Myc-PPD1*
V6-PPD1R	CGATTTCGAACCCGGGGTACCTTAAATGCCTTCACTGTTTAG	*Myc-PPD1*
V6-PPD2F	GAGGACTTGAATTCGGTACCCATGGATGTAGGAGTTACTACG	*Myc-PPD2*
V6-PPD2R	CGATTTCGAACCCGGGGTACCTTAATTATCTTCGCTGTTTAGA	*Myc-PPD2*
V6-KIX8F	GAGGACTTGAATTCGGTACCCATGCCGAGGCCAGGACCAAG	*Myc-KIX8*
V6-KIX8R	CGATTTCGAACCCGGGGTACCCTAAAGGAAGTCTCCACACA	*Myc-KIX8*
V6-KIX9F	GAGGACTTGAATTCGGTACCCATGCCGAGGCCAGGGCCAAG	*Myc-KIX9*
V6-KIX9R	CGATTTCGAACCCGGGGTACCTCAGTTGTTATTGTTGCTGCT	*Myc-KIX9*
V6-MYC3F	GAGGACTTGAATTCGGTACCCATGAACGGCACAACATCATC	*Myc-MYC3*
V6-MYC3R	CGATTTCGAACCCGGGGTACCTCAATAGTTTTCTCCGACT	*Myc-MYC3*
V6-MYC4F	GAGGACTTGAATTCGGTACCCATGTCTCCGACGAATGTTC	*Myc-MYC4*
V6-MYC4R	CGATTTCGAACCCGGGGTACCTCATGGACATTCTCCAACT	*Myc-MYC4*
V6-TPLF	GAGGACTTGAATTCGGTACCCATGTCTTCTCTTAGTAGAG	*Myc-TPL*
V6-TPLR	CGATTTCGAACCCGGGGTACCTCATCTCTGAGGCTGATCAG	*Myc-TPL*
GIF1pro-F	CGCGGTGGAGATCGAATTCCCATGGAGAGGATTCAACATCAACCTCAC	*GIF1pro:LUC*
GIF1pro-R	TTTATGTTTTTGGCGTCTTCCATGGGTGCTGTTGCATTTCTTTTGC	*GIF1pro:LUC*
V46-KIX8F	AATGGGTCGCGGATCCGAATTCATGCCGAGGCCAGGACCAAGAC	*His-KIX8*
V46-KIX8R	TTGTCGACGGAGCTCGAATTCATGCCGAGGCCAGGACCAAGAC	*His-KIX8*
V46-KIX9F	AATGGGTCGCGGATCCGAATTCATGCCGAGGCCAGGGCCAAGAC	*His-KIX9*
V46-KIX9R	TTGTCGACGGAGCTCGAATTCTCAGTTGTTATTGTTGCTGCT	*His-KIX9*
GW-GIF1F	GGGGACAAGTTTGTACAAAAAAGCAGGCTTC ATGCAACAGCACCTGATGCAGAT	*35S:GIF1*
GW-GIF1R	GGGGACCACTTTGTACAAGAAAGCTGGGTC TCAATTCCCATCATCTGATG	*35S:GIF1*
KIX8pro-F	TAGAGGATCCCCGGGTACGTAATACAGCAACCTCTCCCAC	*KIX8pro:KIX8-GFP*
KIX8pro-R	CTTGGTCCTGGCCTCGGCAT	*KIX8pro:KIX8-GFP*
KIX8-F	ATGCCGAGGCCAGGACCAAG	*KIX8pro:KIX8-GFP*
KIX8-R	GTACCGGTAGAAAAAATGAGAAGGAAGTCTCCACACAAAG	*KIX8pro:KIX8-GFP*
KIX9pro-F	TAGAGGATCCCCGGGTACTTCGTCTTCTCTCGAGTTGTC	*KIX9pro:KIX9-GFP*
KIX9pro-R	CTTGGCCCTGGCCTCGGCAT	*KIX9pro:KIX9-GFP*

KIX9-F	ATGCCGAGGCCAGGGCCAAG	*KIX9pro:KIX9-GFP*
KIX9-R	GTACCGGTAGAAAAAATGAGGTTGTTATTGTTGCTGCTTATC	*KIX9pro:KIX9-GFP*
PPD1pro-F	TAGAGGATCCCCGGGTACGGAGACTTGATGCTGATAAC	*PPD1pro:PPD1-GFP*
PPD1pro-R	GGTGAAACTCCGACATCCAT	*PPD1pro:PPD1-GFP*
PPD1-F	ATGGATGTCGGAGTTTCACC	*PPD1pro:PPD1-GFP*
PPD1-R	GTACCGGTAGAAAAAATGAGAATGCCTTCACTGTTTAGATC	*PPD1pro:PPD1-GFP*
PPD2pro-F	TAGAGGATCCCCGGGTACGATCCATTTCTTCGATAACGAC	*PPD2pro:PPD2-GFP*
PPD2pro-R	CGTAGTAACTCCTACATCCAT	*PPD2pro:PPD2-GFP*
PPD2-F	ATGGATGTAGGAGTTACTACG	*PPD2pro:PPD2-GFP*
PPD2-R	GTACCGGTAGAAAAAATGAGATTATCTTCGCTGTTTAGATC	*PPD2pro:PPD2-GFP*
MYC3pro-F	TAGAGGATCCCCGGGTACAAGTCACACAATTCTAAATC	*MYC3pro:MYC3-GFP*
MYC3pro-R	GATGATGTTGTGCCGTTCAT	*MYC3pro:MYC3-GFP*
MYC3-F	ATGAACGGCACAACATCATC	*MYC3pro:MYC3-GFP*
MYC3-R	GTACCGGTAGAAAAAATGAGATAGTTTTCTCCGACTTTCG	*MYC3pro:MYC3-GFP*
MYC4pro-F	TAGAGGATCCCCGGGTACCAACGTAATGCTACACATTAG	*MYC4pro:MYC4-GFP*
MYC4pro-R	CTTGAACATTCGTCGGAGACAT	*MYC4pro:MYC4-GFP*
MYC4-F	ATGTCTCCGACGAATGTTCAAG	*MYC4pro:MYC4-GFP*
MYC4-R	GTACCGGTAGAAAAAATGAGTGGACATTCTCCAACTTTCTC	*MYC4pro:MYC4-GFP*
GIF1pro-F	TAGAGGATCCCCGGGTACAGAGGATTCAACATCAACCTCAC	*GIF1pro:GIF1-GFP*
GIF1pro-R	CTGCATCAGGTGCTGTTGCAT	*GIF1pro:GIF1-GFP*
GIF1-F	ATGCAACAGCACCTGATGCAG	*GIF1pro:GIF1-GFP*
GIF1-R	GTACCGGTAGAAAAAATGAGATTCCCATCATCTGATGATTTC	*GIF1pro:GIF1-GFP*
ChIPF1F	TGTGTACATATTGATAAGTAG	ChIP-qPCR
ChIPF1R	CTTTCTGTCTCATCCTATCTC	ChIP-qPCR
ChIPF2F	CCAAAACAAAATAAGACGAC	ChIP-qPCR
ChIPF2R	GGATTTTATAATAAAGTAATGC	ChIP-qPCR
ChIPF3F	AGATAACTATTGGTAAGCGTAAG	ChIP-qPCR
ChIPF3R	TTGTTTTAAAAGTAGCGCATG	ChIP-qPCR
ChIPF4F	GAATTGATCATTTGTAAGCG	ChIP-qPCR
ChIPF4R	CAATAGTGTTTCTATGCCATC	ChIP-qPCR
Actin7-ChIPF	TGTTGGTCGTCCTAGGCAC	ChIP-qPCR
Actin7-ChIPR	ATGCCATATCTTTTCCATGTC	ChIP-qPCR
