# Supplementary material for: Transcriptional repression of GIF1 by the KIX-PPD-MYC repressor complex controls seed size in Arabidopsis
Source: Nat Commun. 2020 Apr 15;11:1846. doi: 10.1038/s41467-020-15603-3 (PMC7160150; doi:10.1038/s41467-020-15603-3)
Supplement: Supplementary file 4 — Reporting Summary [file 41467_2020_15603_MOESM4_ESM.pdf]

nature research

Corresponding author(s): Yunhai Li
Last updated by author(s): Mar 9, 2020

Reporting Summary

Nature Research wishes to improve the reproducibility of the work that we publish. This form provides structure for consistency and transparency in reporting. For further information on Nature Research policies, see Authors & Referees and the Editorial Policy Checklist .

Statistics

For all statistical analyses, confirm that the following items are present in the figure legend, table legend, main text, or Methods section.

n/a	Confirmed	
☐	☒	The exact sample size (*n*) for each experimental group/condition, given as a discrete number and unit of measurement
☐	☒	A statement on whether measurements were taken from distinct samples or whether the same sample was measured repeatedly
☐	☒	The statistical test(s) used AND whether they are one- or two-sided<br>*Only common tests should be described solely by name; describe more complex techniques in the Methods section.*
☒	☐	A description of all covariates tested
☒	☐	A description of any assumptions or corrections, such as tests of normality and adjustment for multiple comparisons
☐	☒	A full description of the statistical parameters including central tendency (e.g. means) or other basic estimates (e.g. regression coefficient) AND variation (e.g. standard deviation) or associated estimates of uncertainty (e.g. confidence intervals)
☐	☒	For null hypothesis testing, the test statistic (e.g. *F*, *t*, *r*) with confidence intervals, effect sizes, degrees of freedom and *P* value noted<br>*Give P values as exact values whenever suitable.*
☒	☐	For Bayesian analysis, information on the choice of priors and Markov chain Monte Carlo settings
☒	☐	For hierarchical and complex designs, identification of the appropriate level for tests and full reporting of outcomes
☒	☐	Estimates of effect sizes (e.g. Cohen's *d*, Pearson's *r*), indicating how they were calculated

Our web collection on statistics for biologists contains articles on many of the points above.

Software and code

Policy information about availability of computer code

Data collection	The Differential Interference Contrast microscope (DIC, Leica DM2500) was used for the seed and embryo collection. Confocal microscopy (LSM710, Zeiss, Germany) was used for the GFP fluorescence observation. The CCD imaging apparatus (CHEMIPROHT 1300B/LND; Roper Scientific) was used for the luciferase activity observation. Sequencing reads were undertaken with the BGISEQ-500 sequencer.
Data analysis	GraphPad Prism and Microsoft Excel statistical softwares were used for statistical analysis. The ImageJ was used for the integument length, seed size, and cotyledon size analyses.

For manuscripts utilizing custom algorithms or software that are central to the research but not yet described in published literature, software must be made available to editors/reviewers. We strongly encourage code deposition in a community repository (e.g. GitHub). See the Nature Research guidelines for submitting code & software for further information.

Data

Policy information about availability of data

All manuscripts must include a data availability statement. This statement should provide the following information, where applicable:

- Accession codes, unique identifiers, or web links for publicly available datasets
- A list of figures that have associated raw data
- A description of any restrictions on data availability

The RNA-seq data are available from the NCBI Sequence Read Archive (PRJNA610584 [http://www.ncbi.nlm.nih.gov/bioproject/610584]). Other supporting data was shown in Supplementary information and Supplementary Dataset

Field-specific reporting

Please select the one below that is the best fit for your research. If you are not sure, read the appropriate sections before making your selection.

☒ Life sciences ☐ Behavioural & social sciences ☐ Ecological, evolutionary & environmental sciences

For a reference copy of the document with all sections, see nature.com/documents/nr-reporting-summary-flat.pdf

Life sciences study design

All studies must disclose on these points even when the disclosure is negative.

Sample size	Required experimental sample sizes were estimated based on our past experience performing similar experiments, and we have stated it in figure legends.
Data exclusions	No data was excluded from the analyses.
Replication	All experiments were repeated at least three times, and we have stated it in figure legends.
Randomization	All samples were arranged randomly into experimental groups.
Blinding	All the experiments were blinded to group allocation during data collection and/or analysis.

Reporting for specific materials, systems and methods

We require information from authors about some types of materials, experimental systems and methods used in many studies. Here, indicate whether each material, system or method listed is relevant to your study. If you are not sure if a list item applies to your research, read the appropriate section before selecting a response.

Materials & experimental systems

n/a	Involved in the study	
☐	☒	Antibodies
☒	☐	Eukaryotic cell lines
☒	☐	Palaeontology
☒	☐	Animals and other organisms
☒	☐	Human research participants
☒	☐	Clinical data

Methods

n/a	Involved in the study	
☒	☐	ChIP-seq
☒	☐	Flow cytometry
☒	☐	MRI-based neuroimaging

Antibodies

Antibodies used	anti-biotin antibody (Invitrogen, 03-3720), anti-GFP antibody (Invitrogen, MA5-15256), anti-Myc antibody (Abmart, M20002), anti-GST antibody (Abmart, M20007), anti-His antibody (Abmart, M20001), and anti-MBP antibody (Abmart, T40007).
Validation	Information of anti-biotin validation can be found at the product website. <https://www.thermofisher.com/antibody/product/Biotin-Antibody-clone-Z021-Monoclonal/03-3720><br>Information of anti-GFP validation can be found at the product website.<https://www.thermofisher.com/antibody/product/GFP-Antibody-clone-GF28R-Monoclonal/MA5-15256><br>Information of anti-Myc validation can be found at the product website.<http://www.ab-mart.com.cn/page.aspx?node=60&id=962><br>Information of anti-GST validation can be found at the product website.<http://www.ab-mart.com.cn/page.aspx?node=60&id=967><br>Information of anti-His validation can be found at the product website.<http://www.ab-mart.com.cn/page.aspx?node=60&id=959><br>Information of anti-MBP validation can be found at the product website.<http://www.ab-mart.com.cn/page.aspx?node=60&id=1027>
