# Supplementary material for: Transcriptional repression of GIF1 by the KIX-PPD-MYC repressor complex controls seed size in Arabidopsis
Source: Nat Commun. 2020 Apr 15;11:1846. doi: 10.1038/s41467-020-15603-3 (PMC7160150; doi:10.1038/s41467-020-15603-3)
Supplement: Supplementary file 5 — Source Data [file 41467_2020_15603_MOESM5_ESM.zip › List titles of source data.docx]

**Excel 1:** The relative seed area of the Col-0, *kix8-1*, *kix9-1*, *kix8-1 kix9-1*, *ppd1-2*,*ppd2-1*, *ppd1-2 ppd2-cr*, *ppd1-cr ppd2-1* and *kix8-1 kix9-1 ppd1-2 ppd2-cr* plants. (corresponds to Fig. 1c).

**Excel 2:** The relative seed area of the Col-0, *myc3*, *myc4* and *myc3 myc4* plants. (corresponds to Fig. 3c).

**Excel 3:** The relative seed area of the Col-0, *gif1, 35S:GIF1 #4 and* *35S:GIF1 #7* plants. (corresponds to Fig. 5c).

**Excel 4:** The relative seed area of the Col-0, *gif1*, *myc3 myc4*, *gif1 myc3 myc4*, *kix8-1 kix9-1, gif1 kix8-1 kix9-1, ppd1-2 ppd2-cr, gif1 ppd1-2 ppd2-cr, 35S:SAP and* *35S:SAP;gif1* plants. (corresponds to Fig. 7c).

**Excel 5:** The relative seed area of the Col-0, *35S:Myc-KIX8 #2, 35S:Myc-KIX8 #6, 35S:Myc-KIX8 #11, 35S:Myc-KIX9 #4, 35S:Myc-KIX9 #9, 35S:Myc-KIX9 #14, 35S:Myc-PPD1 #1, 35S:Myc-PPD1 #5, 35S:Myc-PPD1 #8, 35S:Myc-PPD2 #2, 35S:Myc-PPD2 #5* and *35S:Myc-PPD2 #7* plants. (corresponds to Supplementary Fig. 9a).

**Excel 6:** The relative seed area of the Col-0, *35S:GFP-MYC3 #2, 35S:GFP-MYC3 #7, 35S:GFP-MYC3 #12, 35S:GFP-MYC4 #1, 35S:GFP-MYC4 #6* and *35S:GFP-MYC4 #8* plants. (corresponds to Supplementary Fig. 10a).

**Excel 7:** The relative seed area of the Col-0, *35S:GFP-KIX8;kix8-1, 35S:GFP-KIX9;kix9-1, 35S:GFP-PPD1;ppd1-2, 35S:GFP-PPD2;ppd2-1, 35S:GFP-MYC3;myc3* and *35S:GFP-MYC4;myc4* plants. (corresponds to Supplementary Fig. 12).

**Excel 8:** 100 seed weight (mg) of the Col-0, *kix8-1*, *kix9-1*, *kix8-1 kix9-1*, *ppd1-2*, *ppd2-1*, *ppd1-2 ppd2-cr*, *ppd1-cr ppd2-1* and *kix8-1 kix9-1 ppd1-2 ppd2-cr* plants. (corresponds to Fig. 1d).

**Excel 9:** 100 seed weight (mg) of the Col-0, *myc3*, *myc4* and *myc3 myc4* plants. (corresponds to Fig. 3d).

**Excel 10:** 100 seed weight (mg) of the Col-0, *gif1, 35S:GIF1 #4 and* *35S:GIF1 #7* plants. (corresponds to Fig. 5d).

**Excel 11:** 100 seed weight (mg) of the Col-0, *gif1*, *myc3 myc4*, *gif1 myc3 myc4*, *kix8-1 kix9-1, gif1 kix8-1 kix9-1, ppd1-2 ppd2-cr, gif1 ppd1-2 ppd2-cr, 35S:SAP and* *35S:SAP;gif1* plants. (corresponds to Fig. 7d).

**Excel 12:** 100 seed weight (mg) of the Col-0, *35S:Myc-KIX8 #2, 35S:Myc-KIX8 #6, 35S:Myc-KIX8 #11, 35S:Myc-KIX9 #4, 35S:Myc-KIX9 #9, 35S:Myc-KIX9 #14, 35S:Myc-PPD1 #1, 35S:Myc-PPD1 #5, 35S:Myc-PPD1 #8, 35S:Myc-PPD2 #2, 35S:Myc-PPD2 #5* and *35S:Myc-PPD2 #7* plants. (corresponds to Supplementary Fig. 9b).

**Excel 13:** 100 seed weight (mg) of the Col-0, *35S:GFP-MYC3 #2, 35S:GFP-MYC3 #7, 35S:GFP-MYC3 #12, 35S:GFP-MYC4 #1, 35S:GFP-MYC4 #6* and *35S:GFP-MYC4 #8* plants. (corresponds to Supplementary Fig. 10b).

**Excel 14:** The relative cotyledon area of the Col-0, *kix8-1*, *kix9-1*, *kix8-1 kix9-1*, *ppd1-2*, *ppd2-1*, *ppd1-2 ppd2-cr*, *ppd1-cr ppd2-1* and *kix8-1 kix9-1 ppd1-2 ppd2-cr* plants. (corresponds to Fig. 1e).

**Excel 15:** The relative cotyledon area of the Col-0, *myc3*, *myc4* and *myc3 myc4* plants. (corresponds to Fig. 3e).

**Excel 16:** The relative cotyledon area of the Col-0, *gif1, 35S:GIF1 #4 and* *35S:GIF1 #7* plants. (corresponds to Fig. 5e).

**Excel 17:** The relative cotyledon area of the Col-0, *gif1*, *myc3 myc4*, *gif1 myc3 myc4*, *kix8-1 kix9-1, gif1 kix8-1 kix9-1, ppd1-2 ppd2-cr, gif1 ppd1-2 ppd2-cr, 35S:SAP and* *35S:SAP;gif1* plants. (corresponds to Fig. 7e).

**Excel 18:** The relative area of F_1_ seeds and F_2_ seeds from Col-0/Col-0 (C/C), Col-0/*kix8-1* *kix9-1* *ppd1-2* *ppd2-cr* (C/kkpp), *kix8-1* *kix9-1* *ppd1-2* *ppd2-cr*/Col-0 (kkpp/C), and *kix8-1* *kix9-1* *ppd1-2* *ppd2-cr*/*kix8-1* *kix9-1* *ppd1-2* *ppd2-cr* (kkpp/kkpp) plants. (corresponds to Fig. 1f-g).

**Excel 19:** The relative area of F_1_ seeds and F_2_ seeds from the Col-0/Col-0 (C/C), Col-0/*myc3* *myc4* (C/mm), *myc3* *myc4*/Col-0 (mm/C), and *myc3* *myc4*/*myc3* *myc4* (mm/mm) plants. (corresponds to Fig. 3f-g).

**Excel 20:** The relative area of F_1_ seeds and F_2_ seeds from Col-0/Col-0 (C/C), Col-0/*gif1* (C/g), *gif1*/Col-0 (g/C), and *gif1*/*gif1*(g/g) plants. (corresponds to Fig. 5f-g).

**Excel 21:** The seed area (×10^4^ um^2^), outer integument length (um), outer integument cell number, and outer integument cell length (um) of Col-0, *kix8-1* *kix9-1* *ppd1-2* *ppd2-cr*, *myc3* *myc4* and *gif1* plants at 0, 2, 4, and 6 DAP. (corresponds to Figs 1i-l, 3i-l and 5i-l).

**Excel 22:** The relative expression levels of *GIF1* in the 0, 2, and 4 DAF (days after flowering) siliques of Col-0, *kix8-1* *kix9-1*, *ppd1-2* *ppd2-cr*, and *myc3* *myc4* were detected by qPCR (n = 3). (corresponds to Fig. 4a).

**Excel 23:** ChIP-qPCR assays showing that KIX8/9 and PPD1/2 associate with the promoter of *GIF1* by *MYC3*/4 in Arabidopsis (n = 4). (corresponds to Fig. 4d).

**Excel 24:** Silique length (SL) and silique width (SW) of Col-0, *gif1*, *myc3* *myc4*, *gif1* *myc3* *myc4*, *kix8-1* *kix9-1*, *gif1* *kix8-1* *kix9-1*, *ppd1-2* *ppd2-cr*, *gif1* *ppd1-2* *ppd2-cr*, *35S:SAP*, and *35S:SAP*;*gif1* plants (n = 35). (corresponds to Supplementary Fig. 15).

**Excel 25:** The LUC activity of GIF1pro:LUC from the transient expression analysis in the Col-0 and myc3 myc4 protoplast (n = 5). (corresponds to Fig. 4b).

**Excel 26:** The relative area of F_1_ seeds and F_2_ seeds from Col-0/Col-0, Col-0/kix8-1, kix8-1/Col-0, and kix8-1/kix8-1 plants (n = 100). (corresponds to Supplementary Fig. 3a-b).

**Excel 27:** The relative area of F_1_ seeds and F_2_ seeds from Col0/Col-0, Col-0/ppd2-1, ppd2-1/Col-0, and ppd2-1/ppd2-1 plants (n = 100). (corresponds to Supplementary Fig. 3c-d).
