# Supplementary figures and images for: Transcriptional repression of GIF1 by the KIX-PPD-MYC repressor complex controls seed size in Arabidopsis
Source: Nat Commun. 2020 Apr 15;11:1846. doi: 10.1038/s41467-020-15603-3 (PMC7160150; doi:10.1038/s41467-020-15603-3)

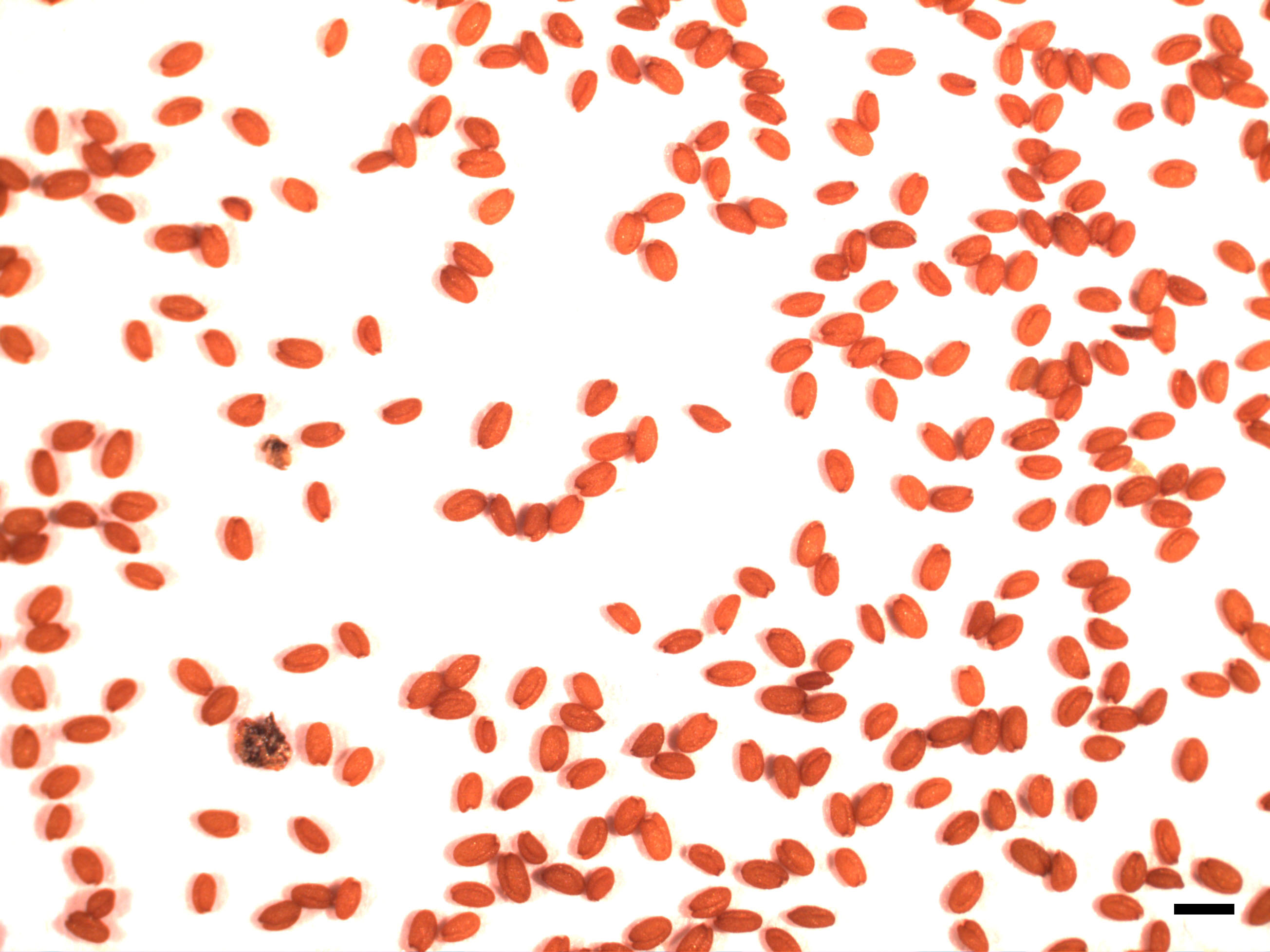

Supplement: Supplementary file 5 — Source Data [file 41467_2020_15603_MOESM5_ESM.zip › seed photos/35S=GFP-MYC3 #12/35S=GFP-MYC3 #12.jpg]

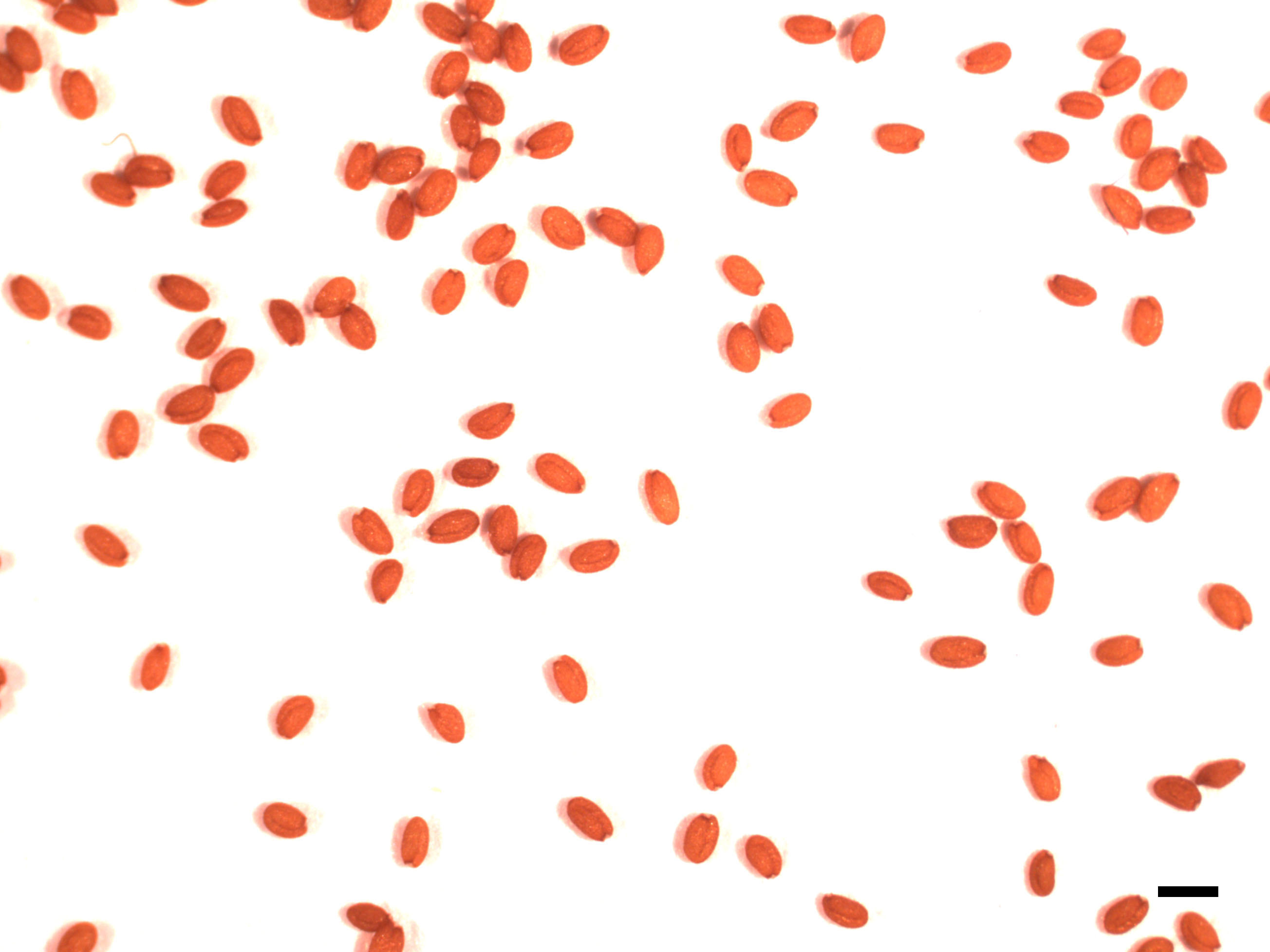

Supplement: Supplementary file 5 — Source Data [file 41467_2020_15603_MOESM5_ESM.zip › seed photos/35S=GFP-MYC3 #2/35S=GFP-MYC3 #2-1.jpg]

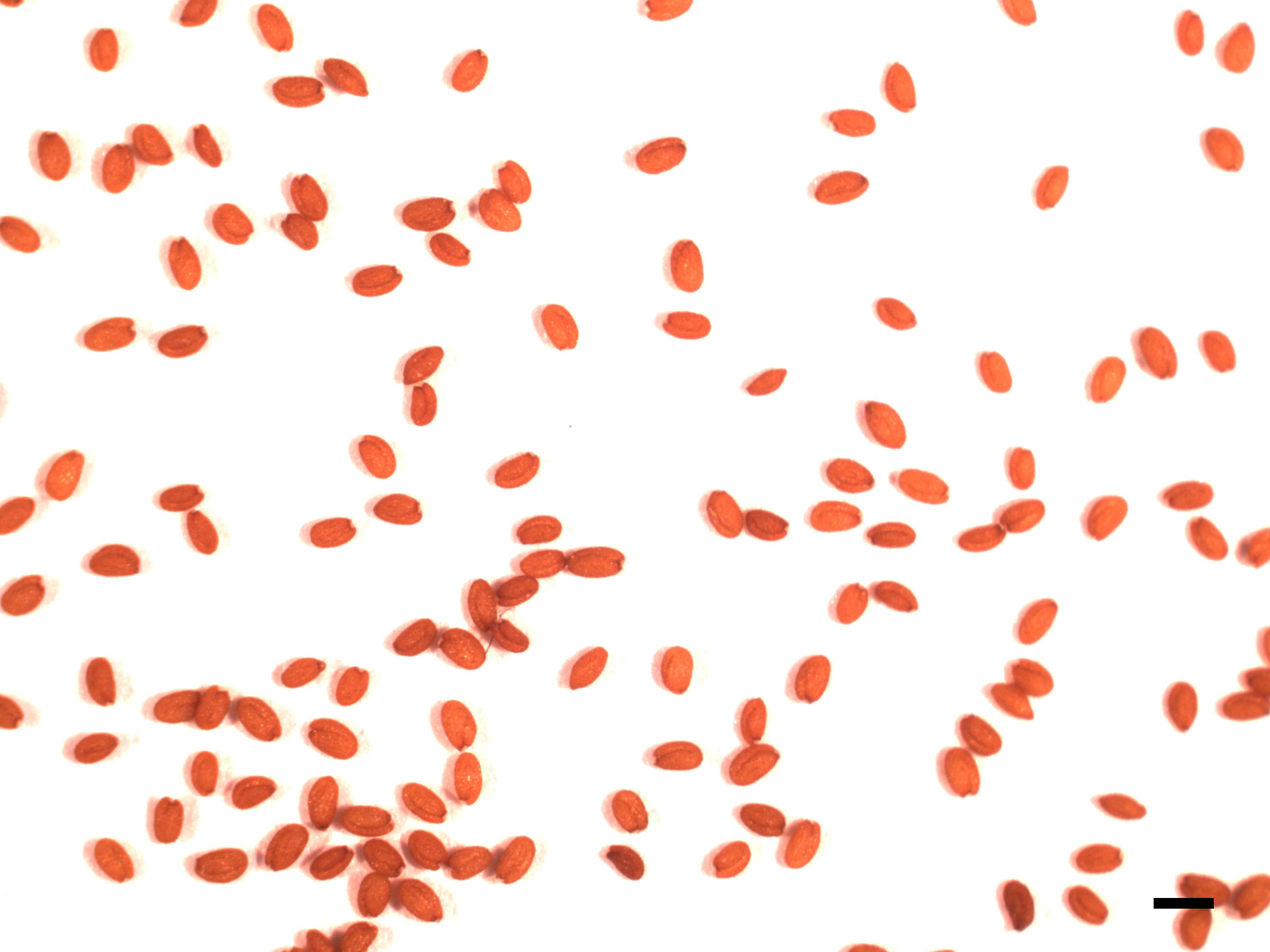

Supplement: Supplementary file 5 — Source Data [file 41467_2020_15603_MOESM5_ESM.zip › seed photos/35S=GFP-MYC3 #2/35S=GFP-MYC3 #2-2.jpg]

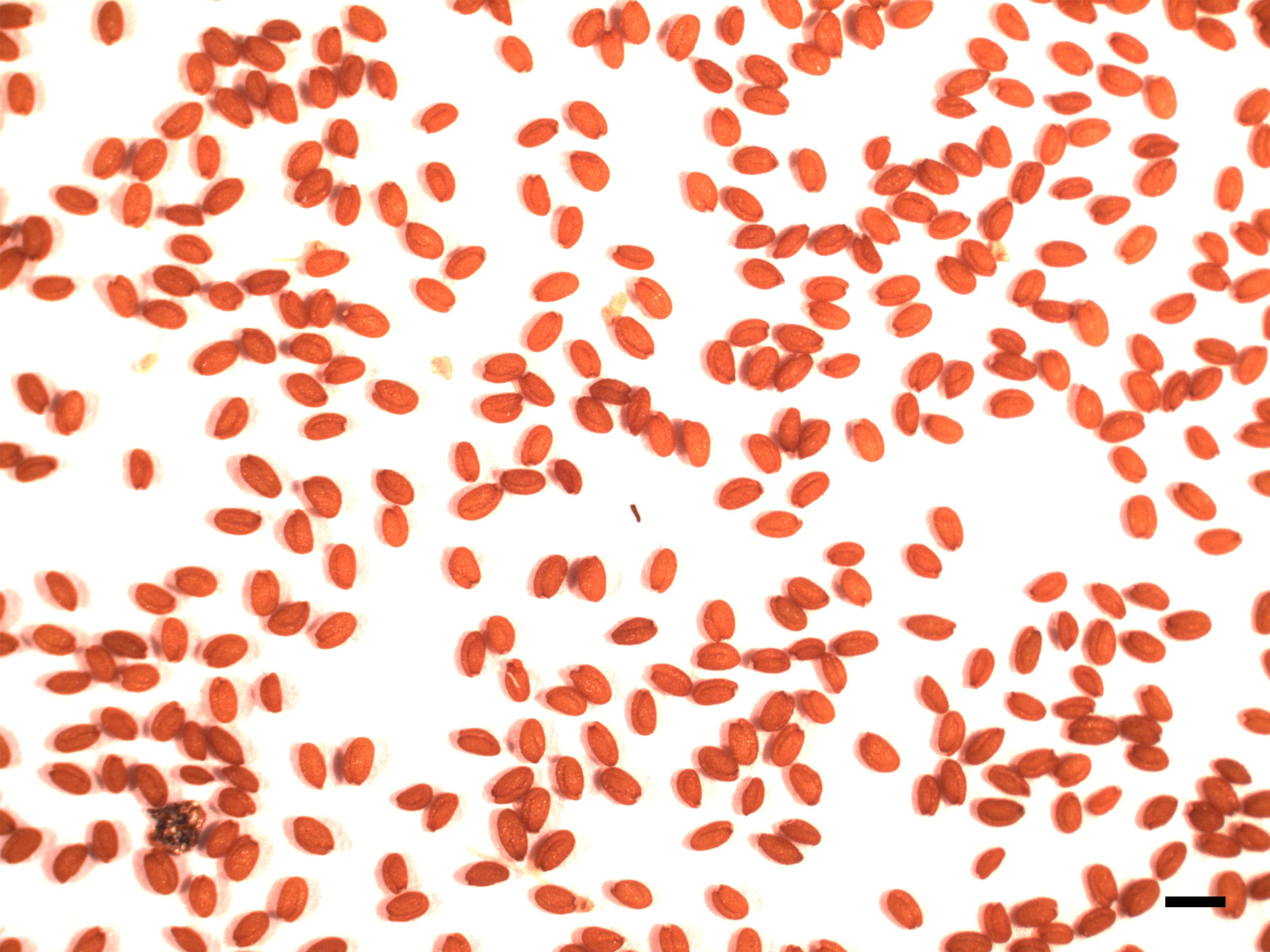

Supplement: Supplementary file 5 — Source Data [file 41467_2020_15603_MOESM5_ESM.zip › seed photos/35S=GFP-MYC3 #7/35S=GFP-MYC3 #7.jpg]

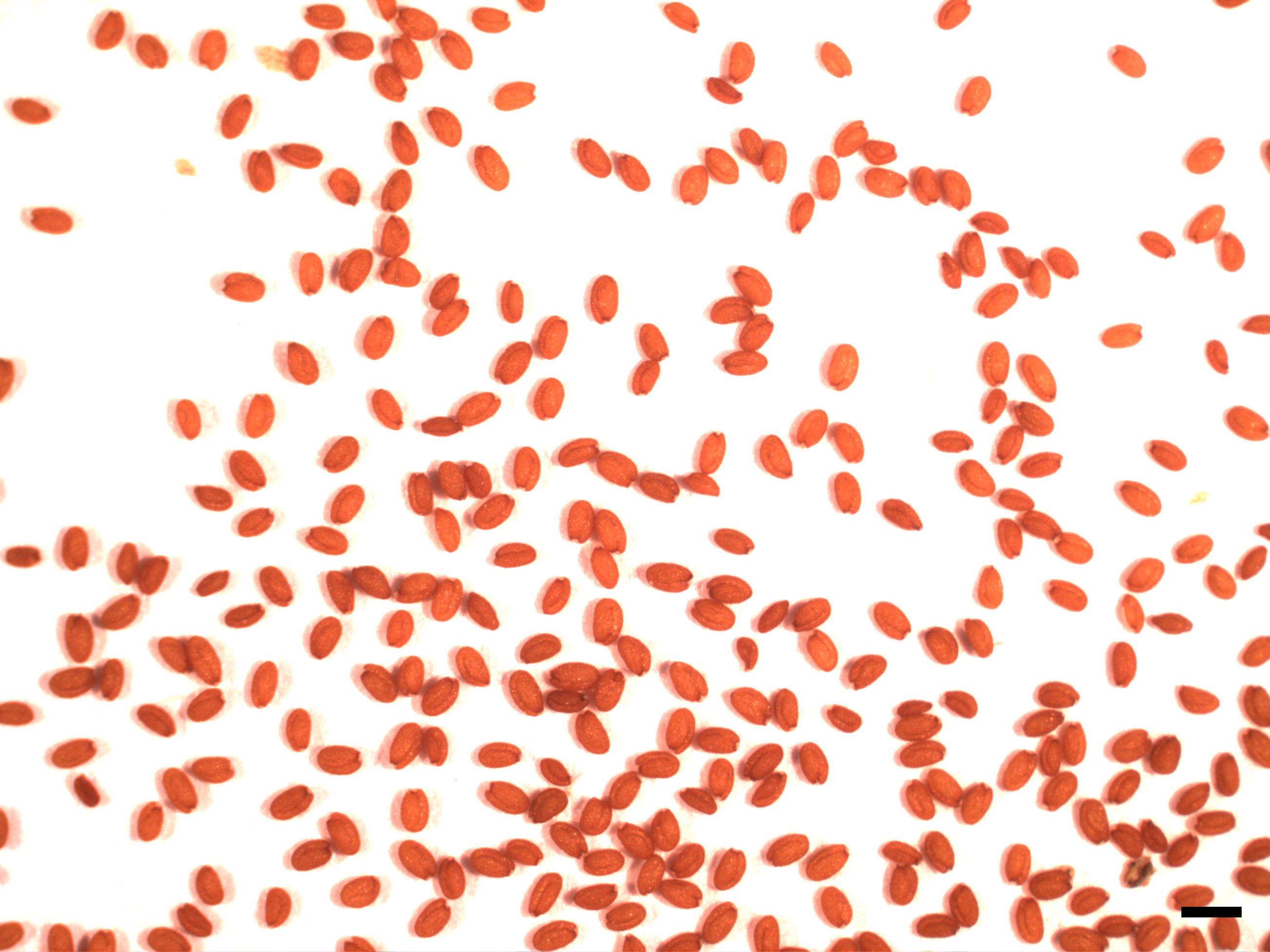

Supplement: Supplementary file 5 — Source Data [file 41467_2020_15603_MOESM5_ESM.zip › seed photos/35S=GFP-MYC4 #1/35S=GFP-MYC4 #1.jpg]

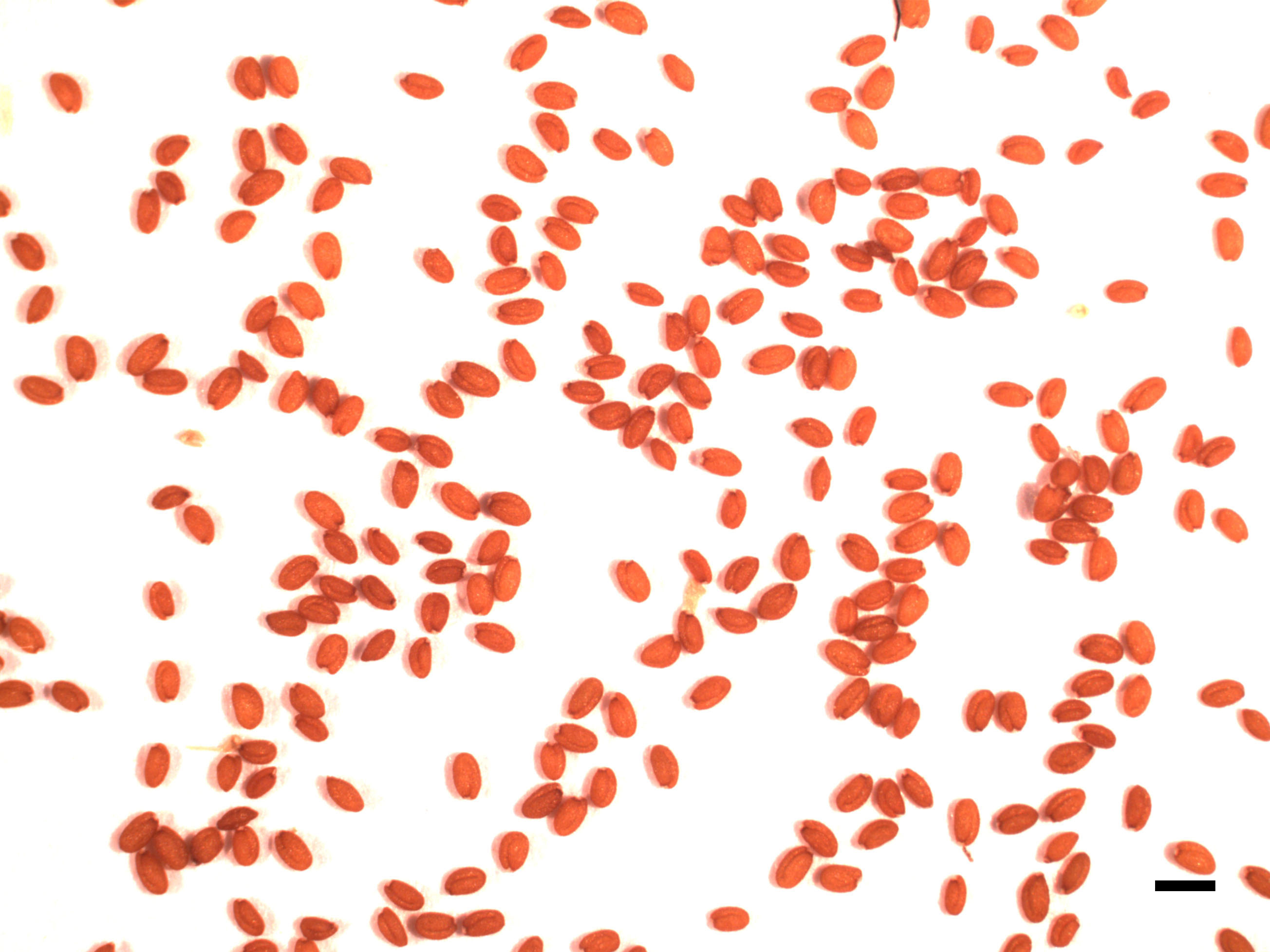

Supplement: Supplementary file 5 — Source Data [file 41467_2020_15603_MOESM5_ESM.zip › seed photos/35S=GFP-MYC4 #6/35S=GFP-MYC4 #6.jpg]

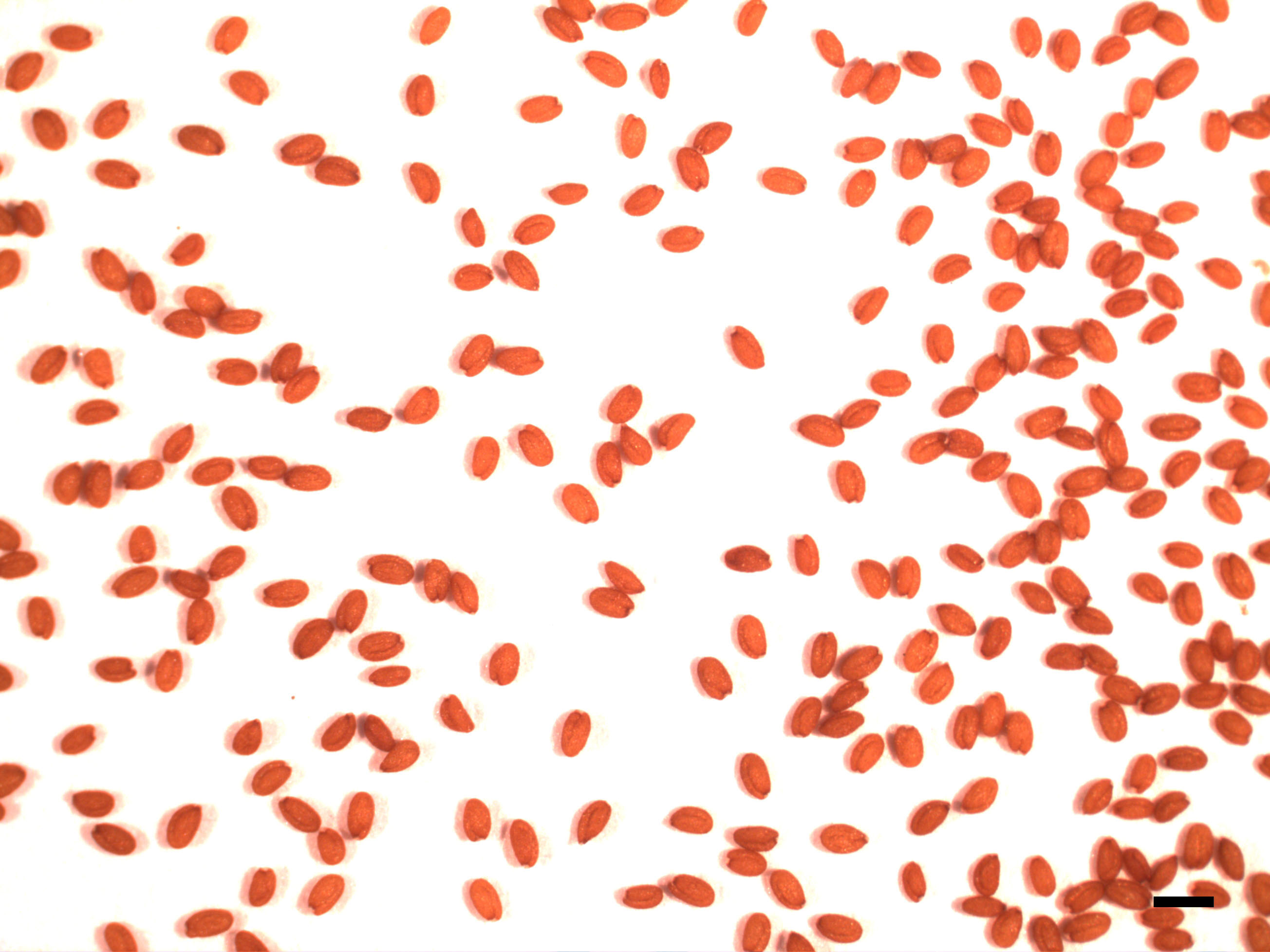

Supplement: Supplementary file 5 — Source Data [file 41467_2020_15603_MOESM5_ESM.zip › seed photos/35S=GFP-MYC4 #8/35S=GFP-MYC4 #8.jpg]

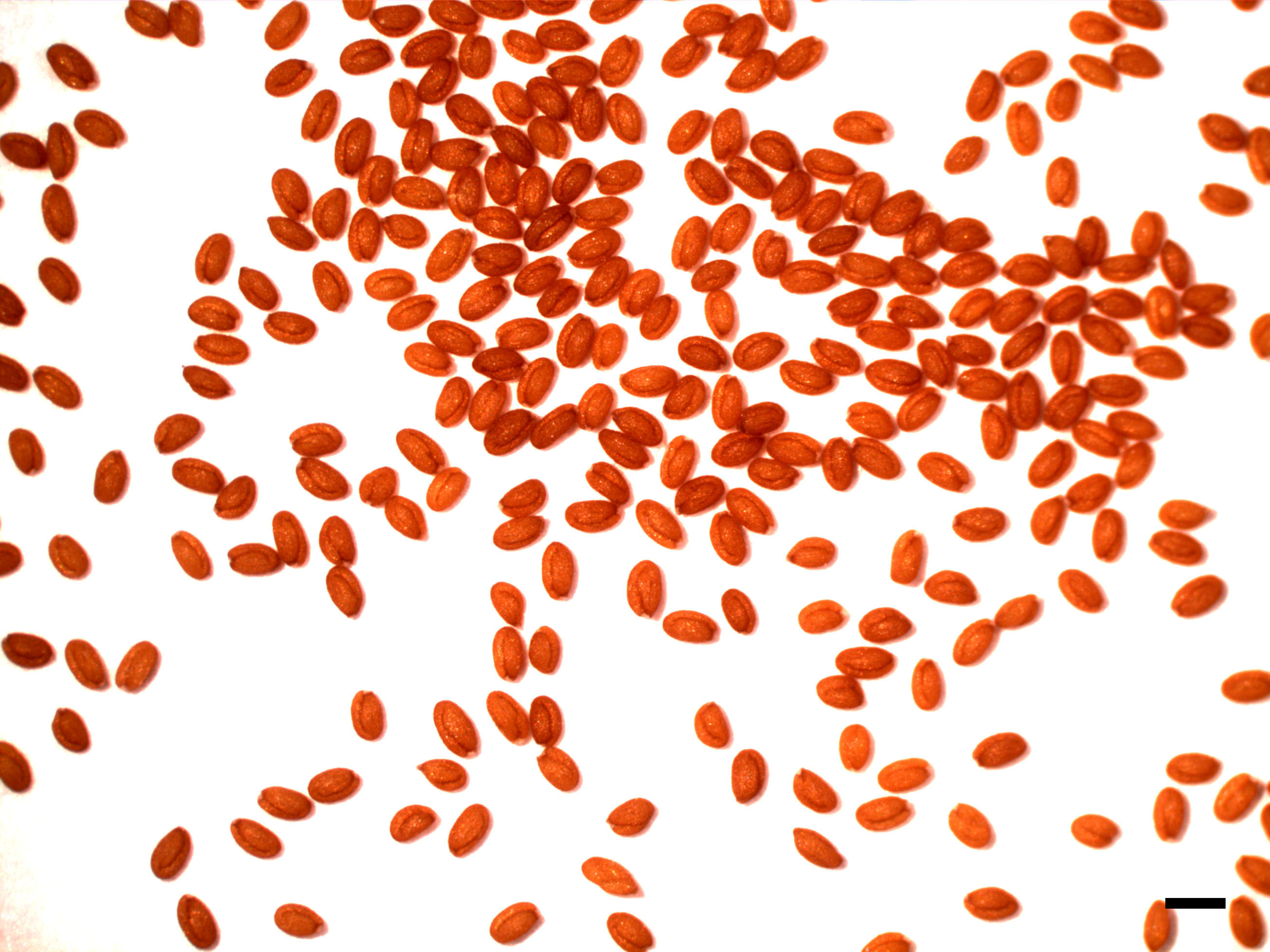

Supplement: Supplementary file 5 — Source Data [file 41467_2020_15603_MOESM5_ESM.zip › seed photos/35S=GIF1 #4/35S=GIF1 #4.jpg]

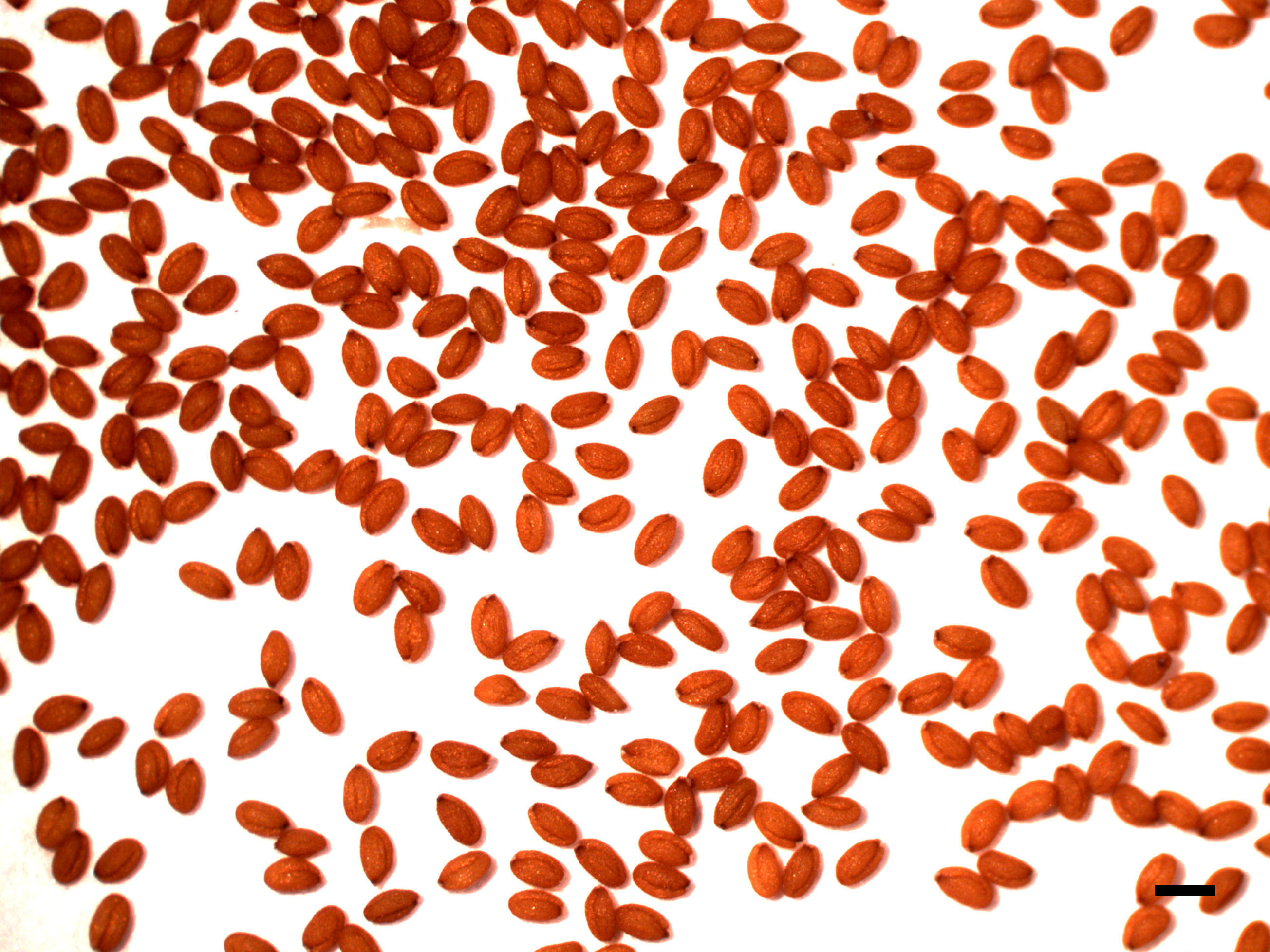

Supplement: Supplementary file 5 — Source Data [file 41467_2020_15603_MOESM5_ESM.zip › seed photos/35S=GIF1 #7/35S=GIF1 #7.jpg]

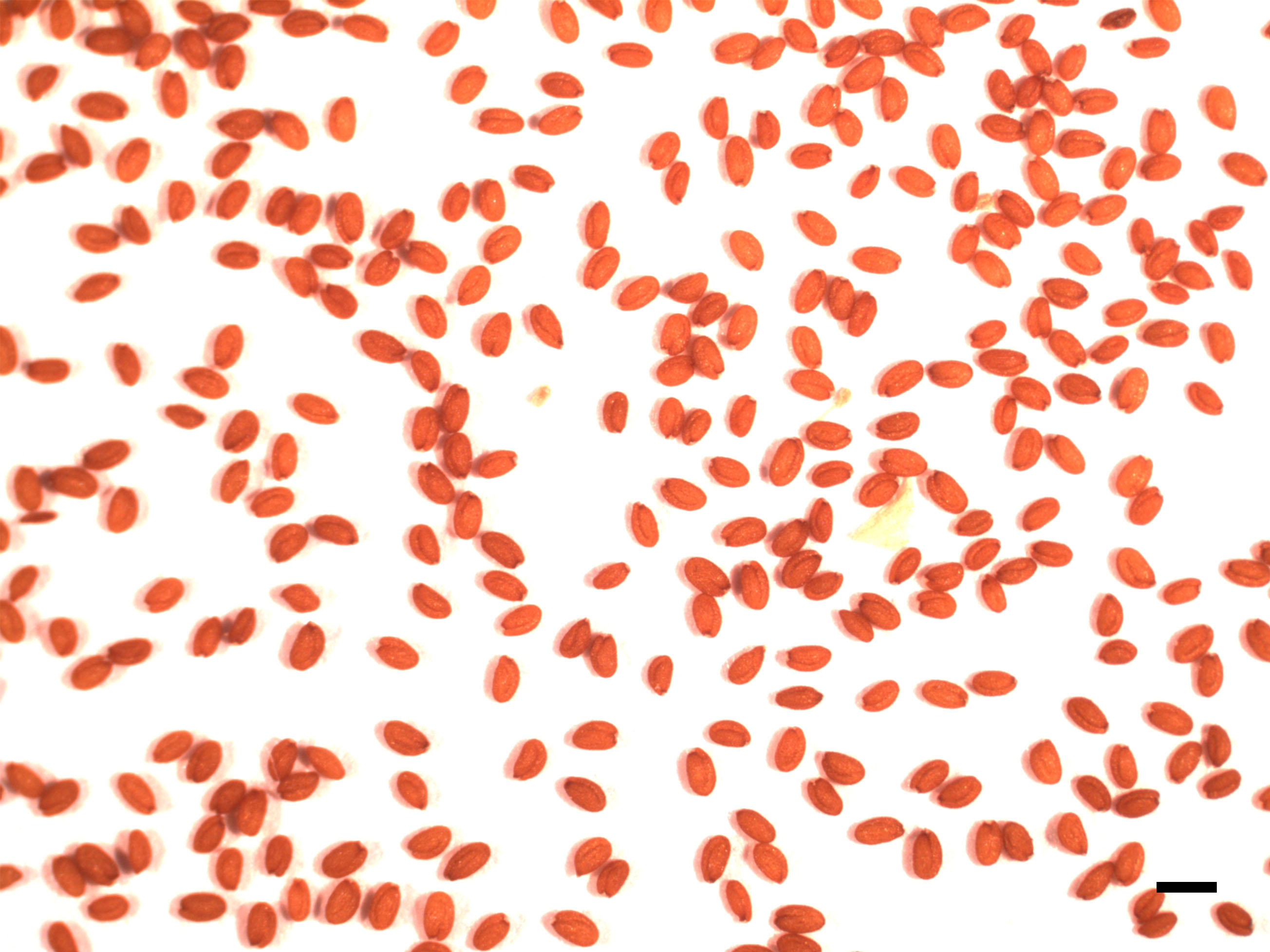

Supplement: Supplementary file 5 — Source Data [file 41467_2020_15603_MOESM5_ESM.zip › seed photos/35S=Myc-KIX8 #11/35S=Myc-KIX8 #11-1.jpg]

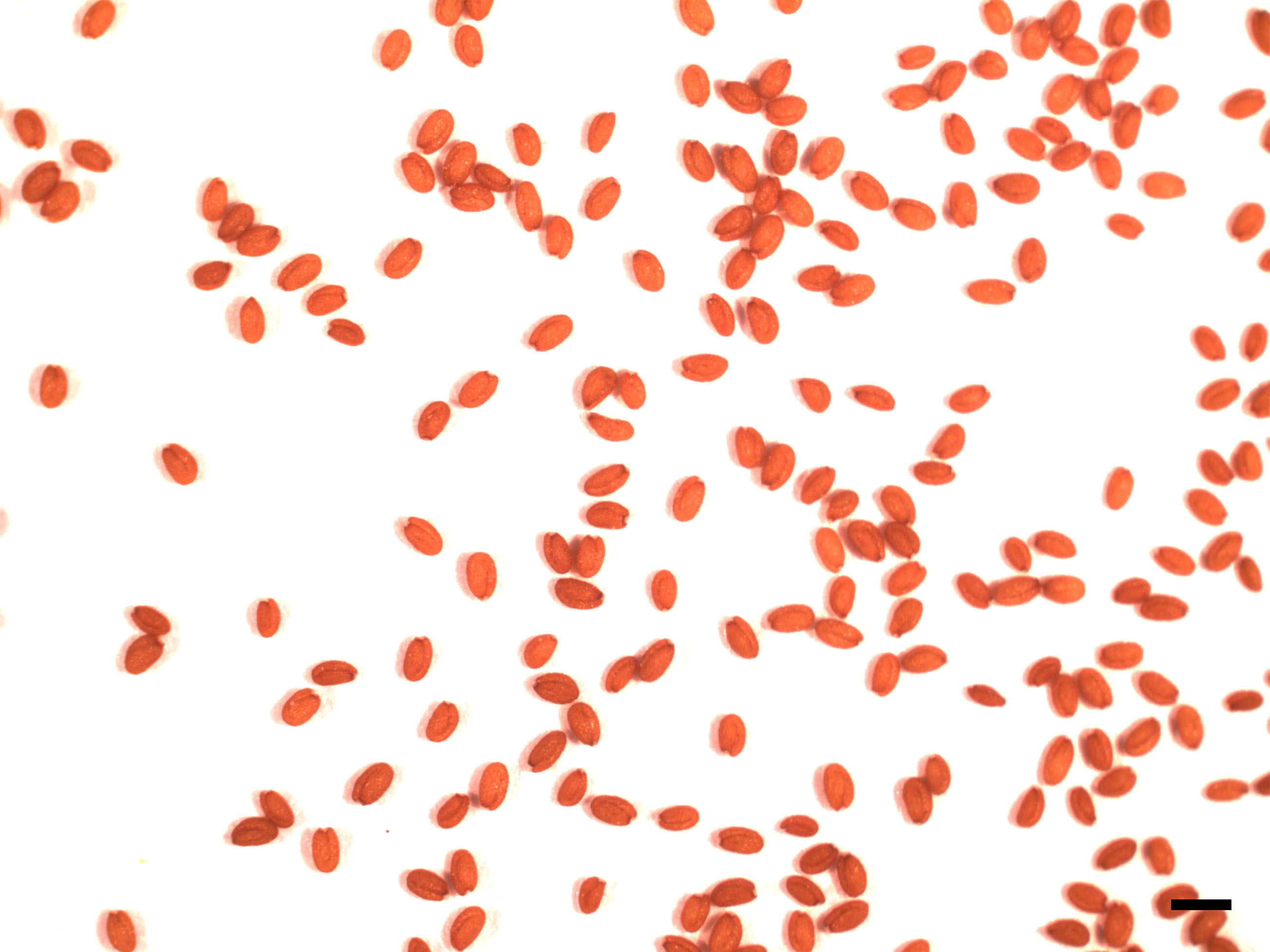

Supplement: Supplementary file 5 — Source Data [file 41467_2020_15603_MOESM5_ESM.zip › seed photos/35S=Myc-KIX8 #11/35S=Myc-KIX8 #11-2.jpg]

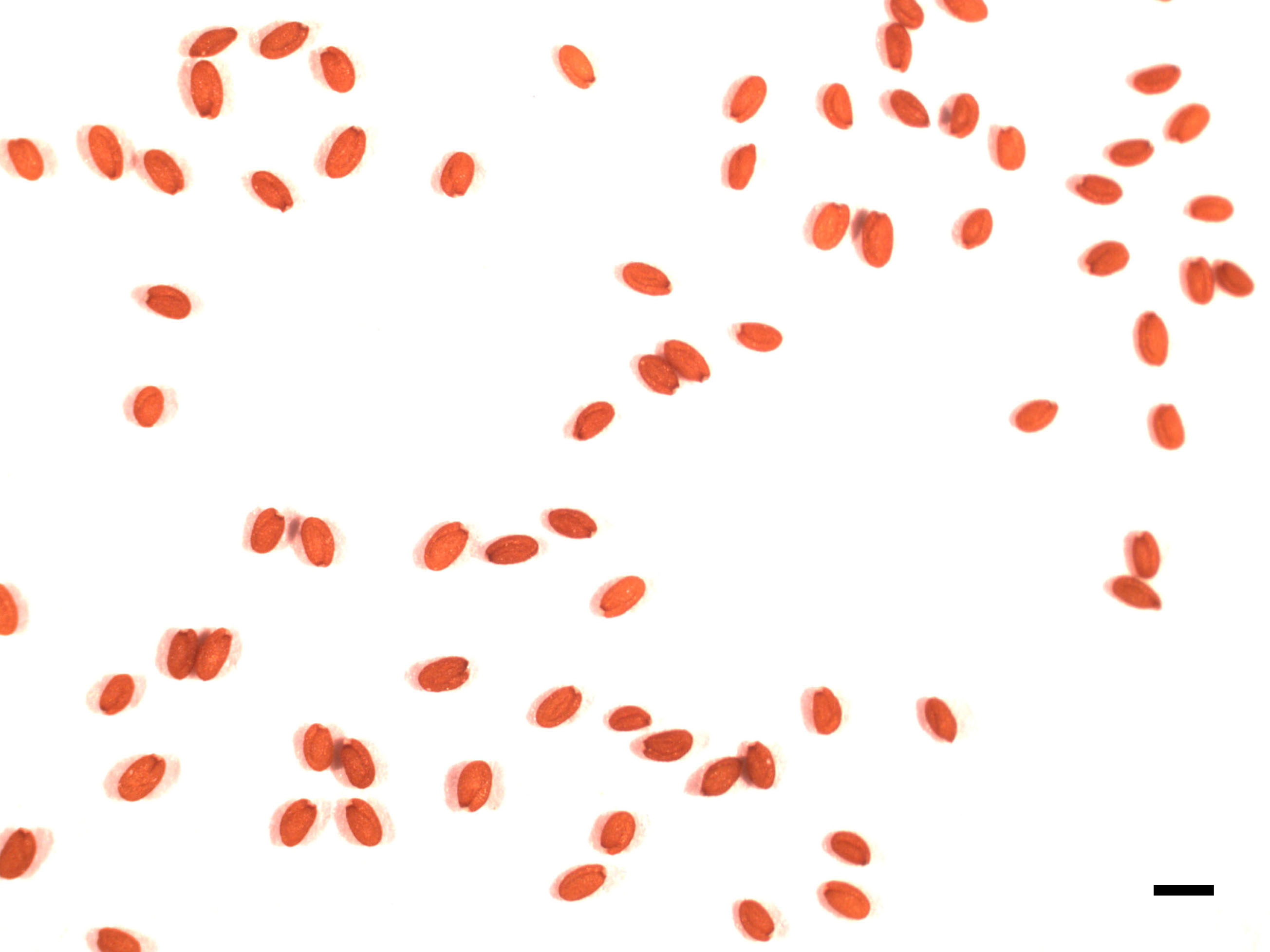

Supplement: Supplementary file 5 — Source Data [file 41467_2020_15603_MOESM5_ESM.zip › seed photos/35S=Myc-KIX8 #2/35S=Myc-KIX8 #2-1.jpg]

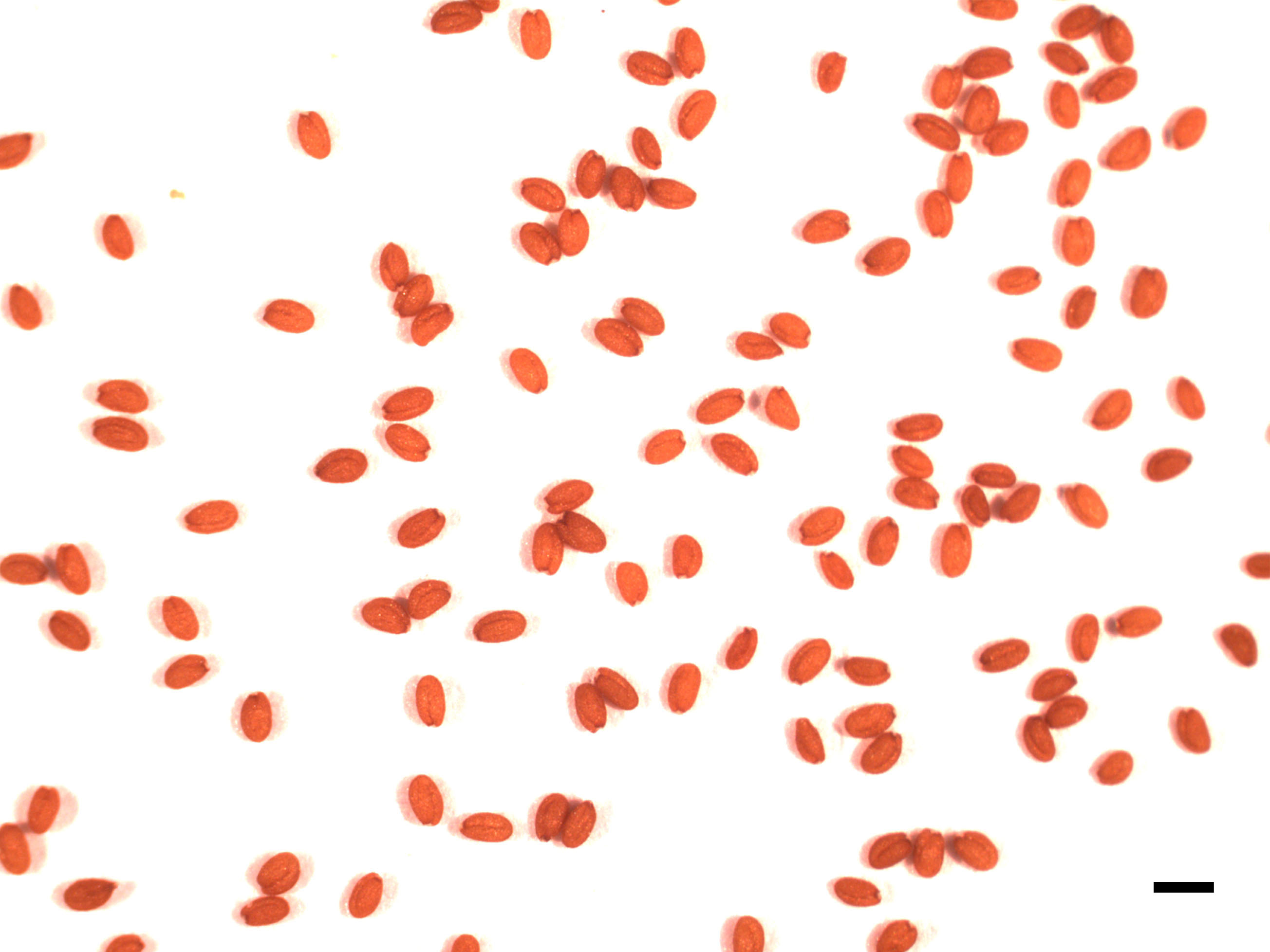

Supplement: Supplementary file 5 — Source Data [file 41467_2020_15603_MOESM5_ESM.zip › seed photos/35S=Myc-KIX8 #2/35S=Myc-KIX8 #2-2.jpg]

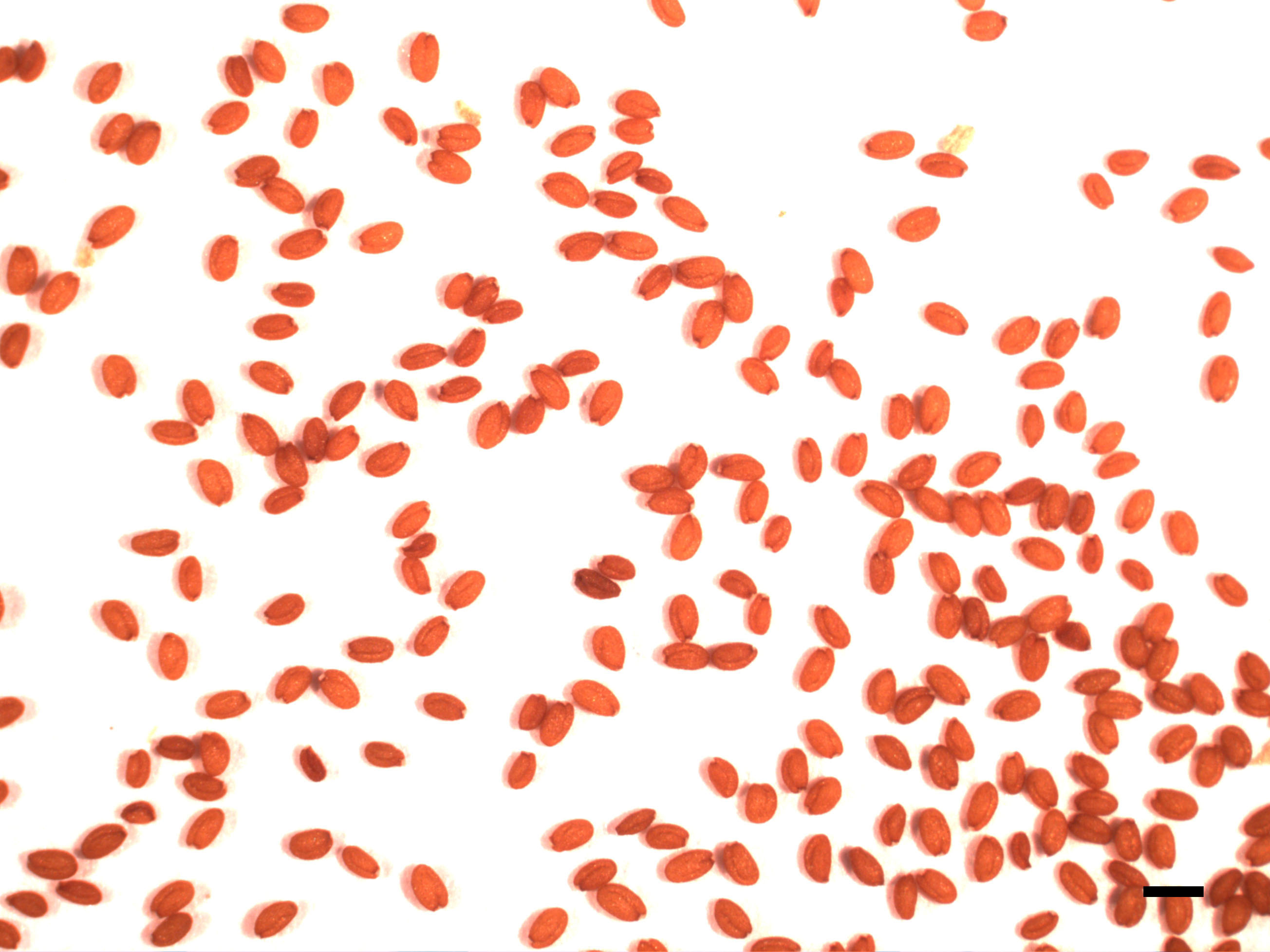

Supplement: Supplementary file 5 — Source Data [file 41467_2020_15603_MOESM5_ESM.zip › seed photos/35S=Myc-KIX8 #6/35S=Myc-KIX8 #6.jpg]

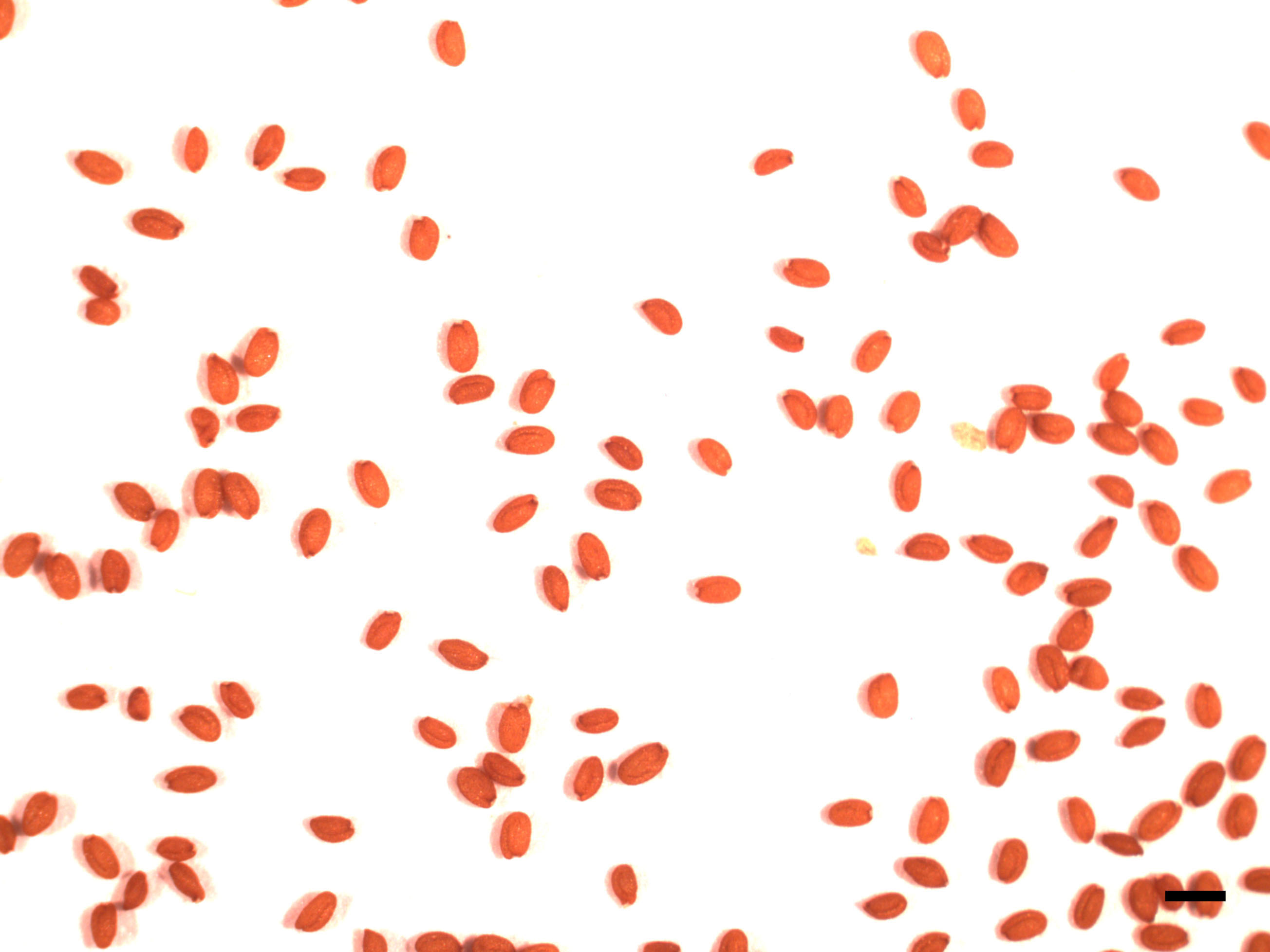

Supplement: Supplementary file 5 — Source Data [file 41467_2020_15603_MOESM5_ESM.zip › seed photos/35S=Myc-KIX9 #14/35S=Myc-KIX9 #14-1.jpg]

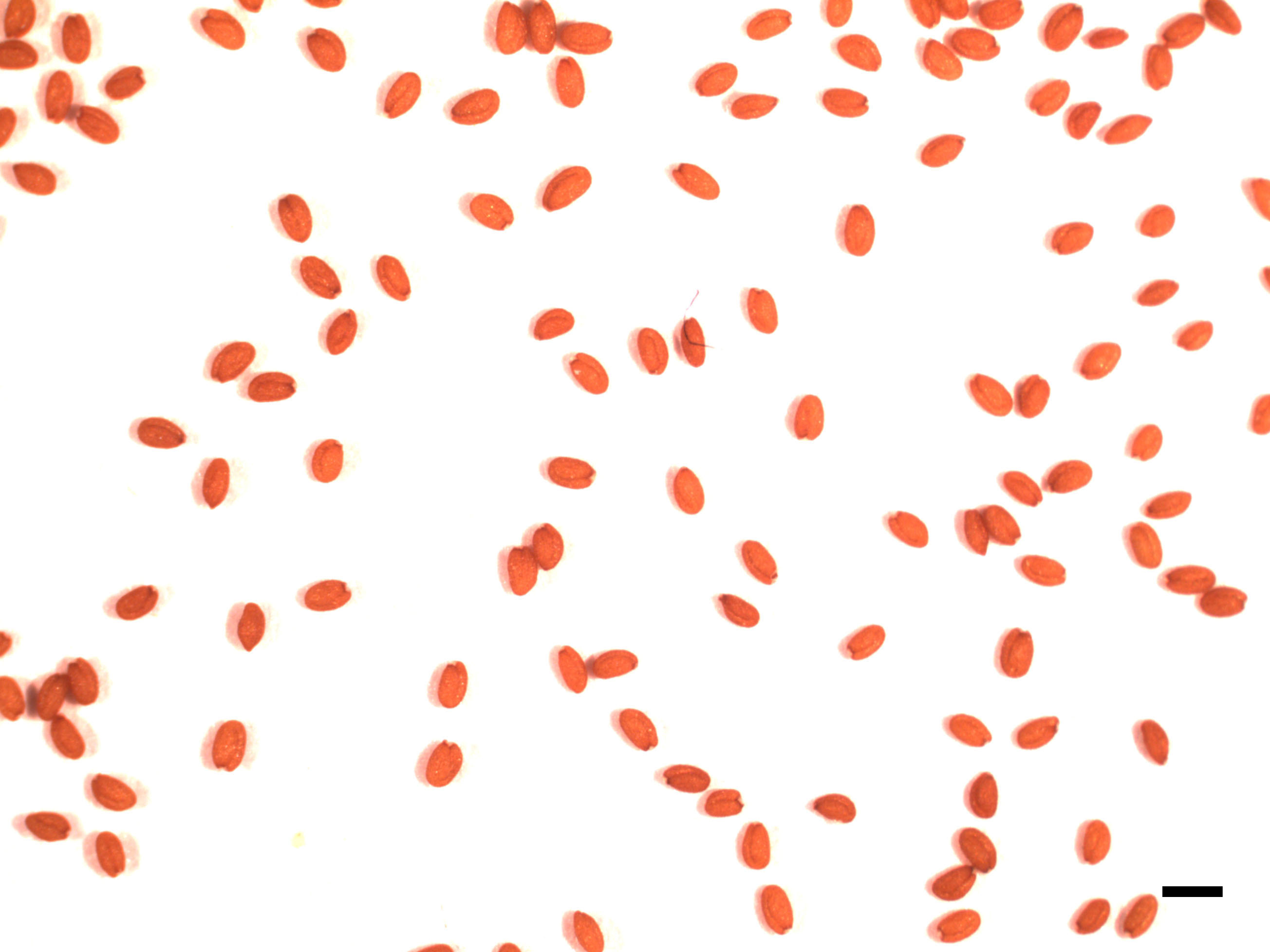

Supplement: Supplementary file 5 — Source Data [file 41467_2020_15603_MOESM5_ESM.zip › seed photos/35S=Myc-KIX9 #14/35S=Myc-KIX9 #14-2.jpg]

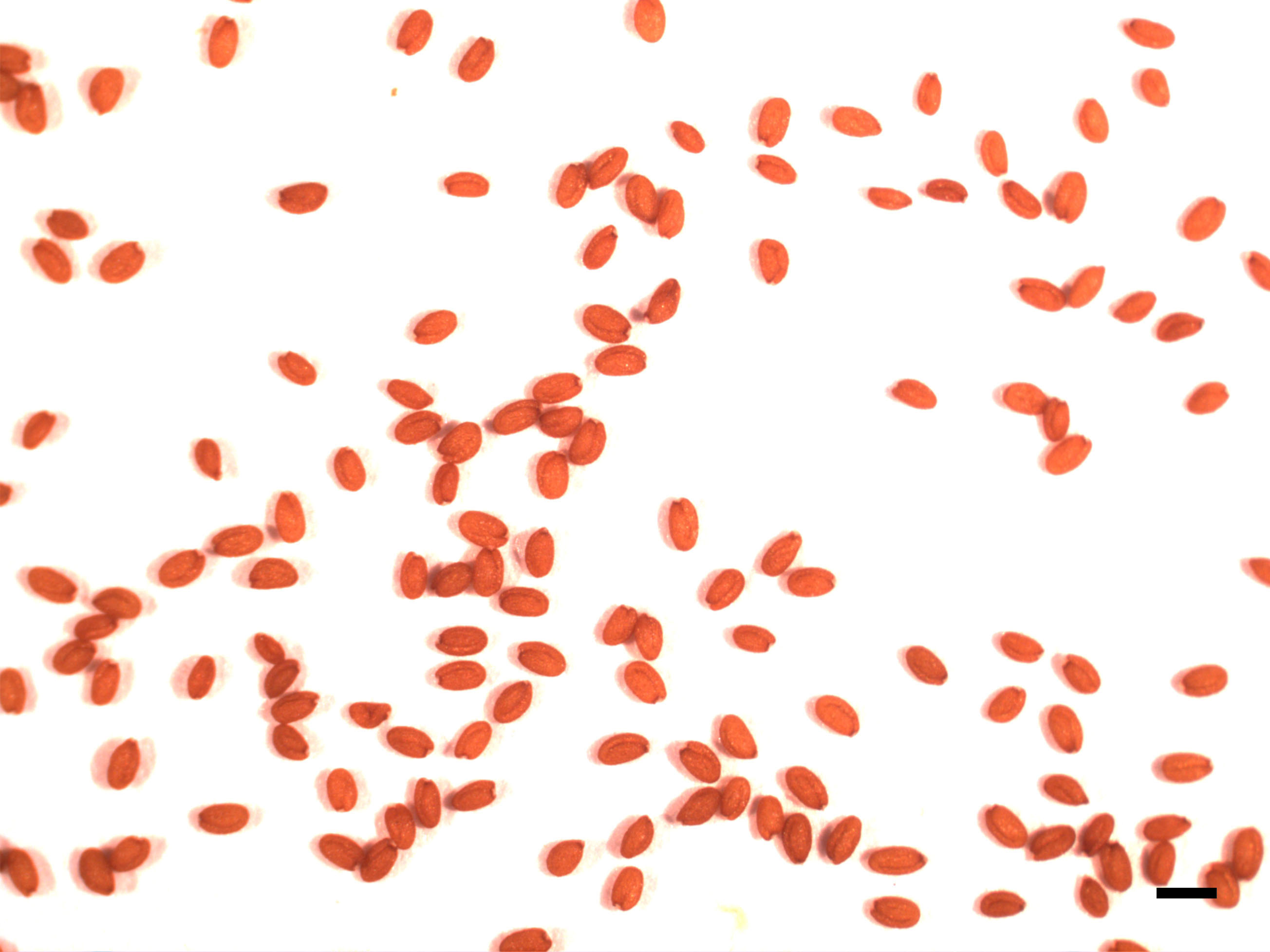

Supplement: Supplementary file 5 — Source Data [file 41467_2020_15603_MOESM5_ESM.zip › seed photos/35S=Myc-KIX9 #4/35S=Myc-KIX9 #4-1.jpg]

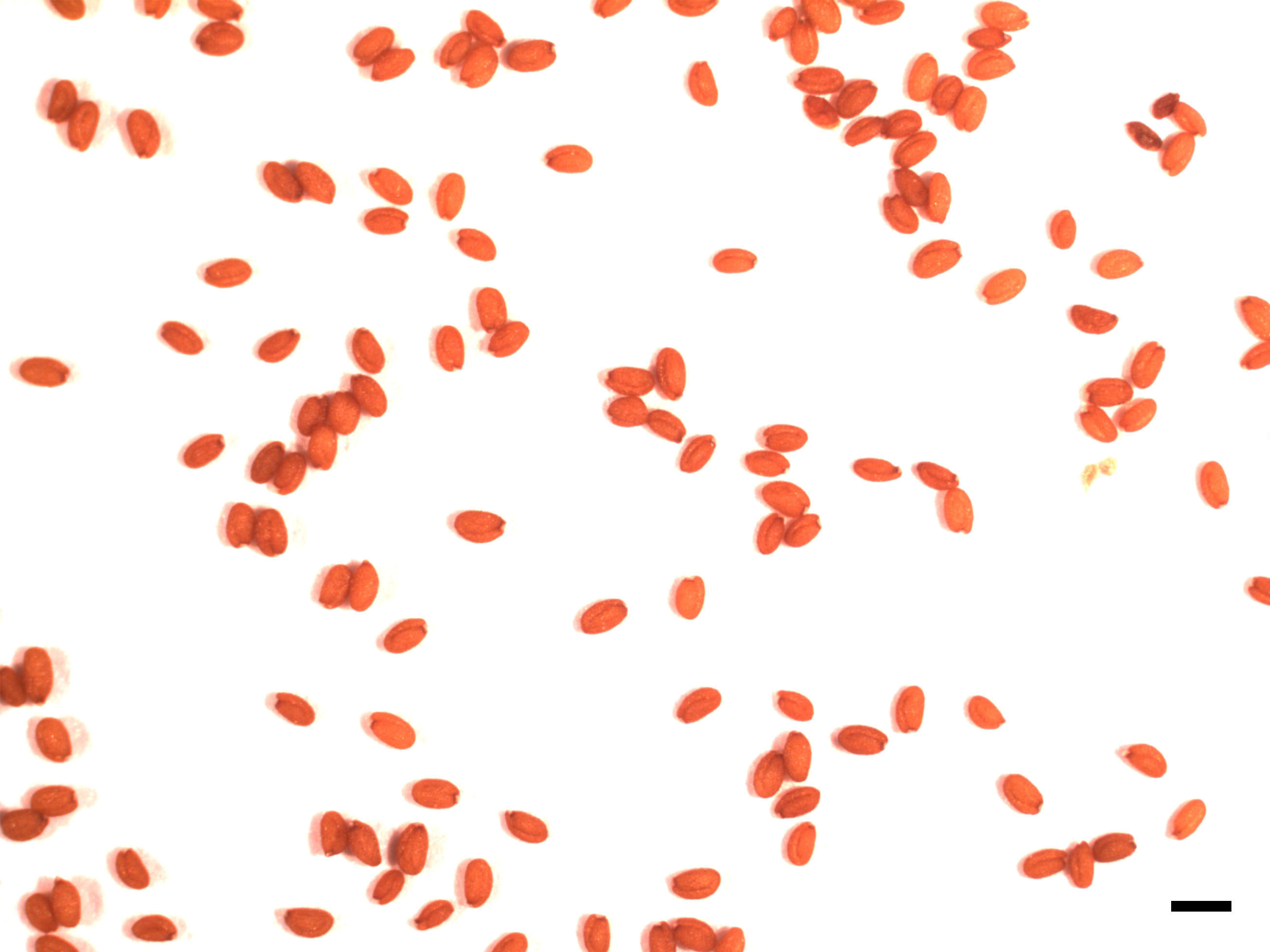

Supplement: Supplementary file 5 — Source Data [file 41467_2020_15603_MOESM5_ESM.zip › seed photos/35S=Myc-KIX9 #4/35S=Myc-KIX9 #4-2.jpg]

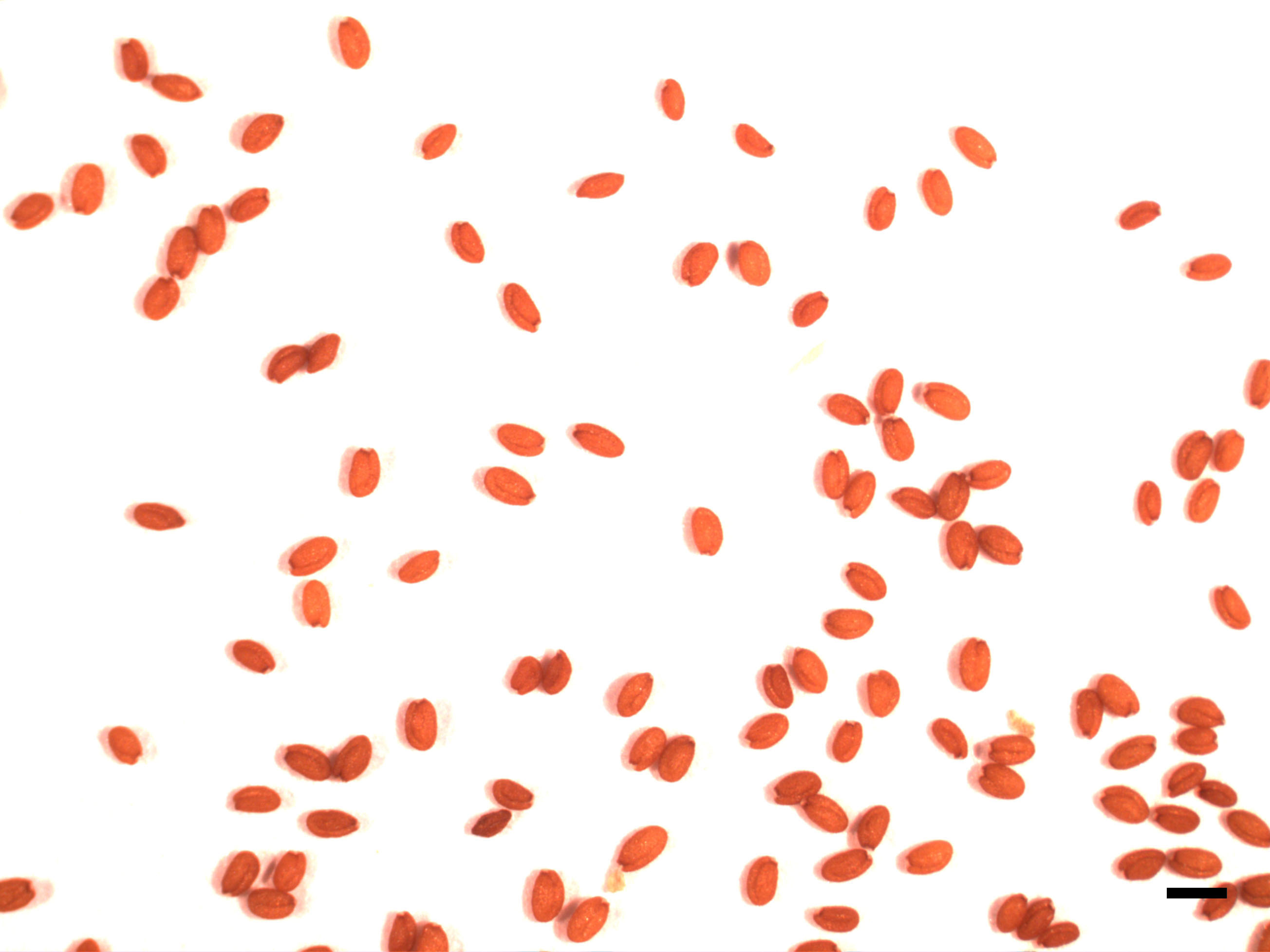

Supplement: Supplementary file 5 — Source Data [file 41467_2020_15603_MOESM5_ESM.zip › seed photos/35S=Myc-KIX9 #9/35S=Myc-KIX9 #9-1.jpg]

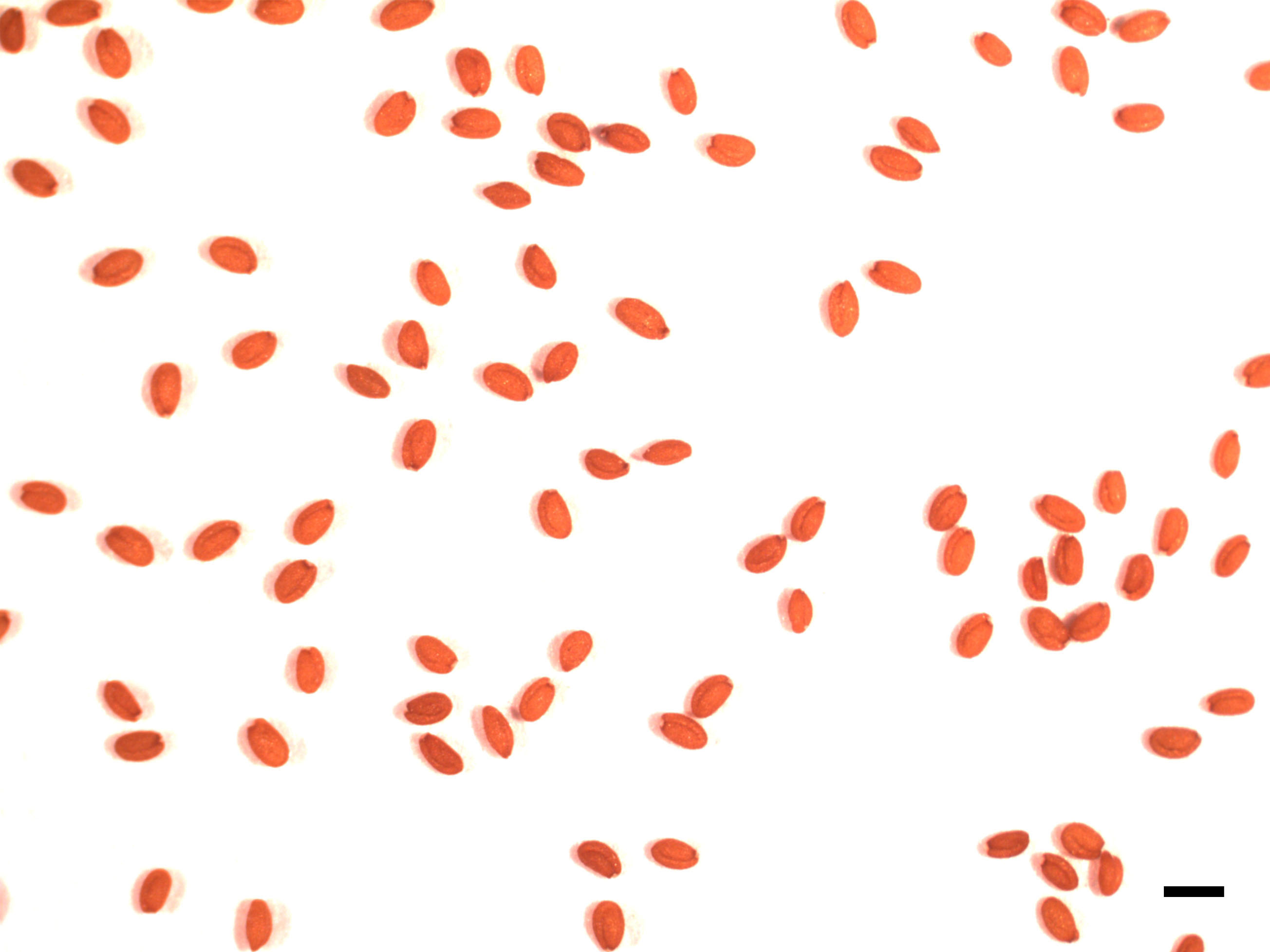

Supplement: Supplementary file 5 — Source Data [file 41467_2020_15603_MOESM5_ESM.zip › seed photos/35S=Myc-KIX9 #9/35S=Myc-KIX9 #9-2.jpg]

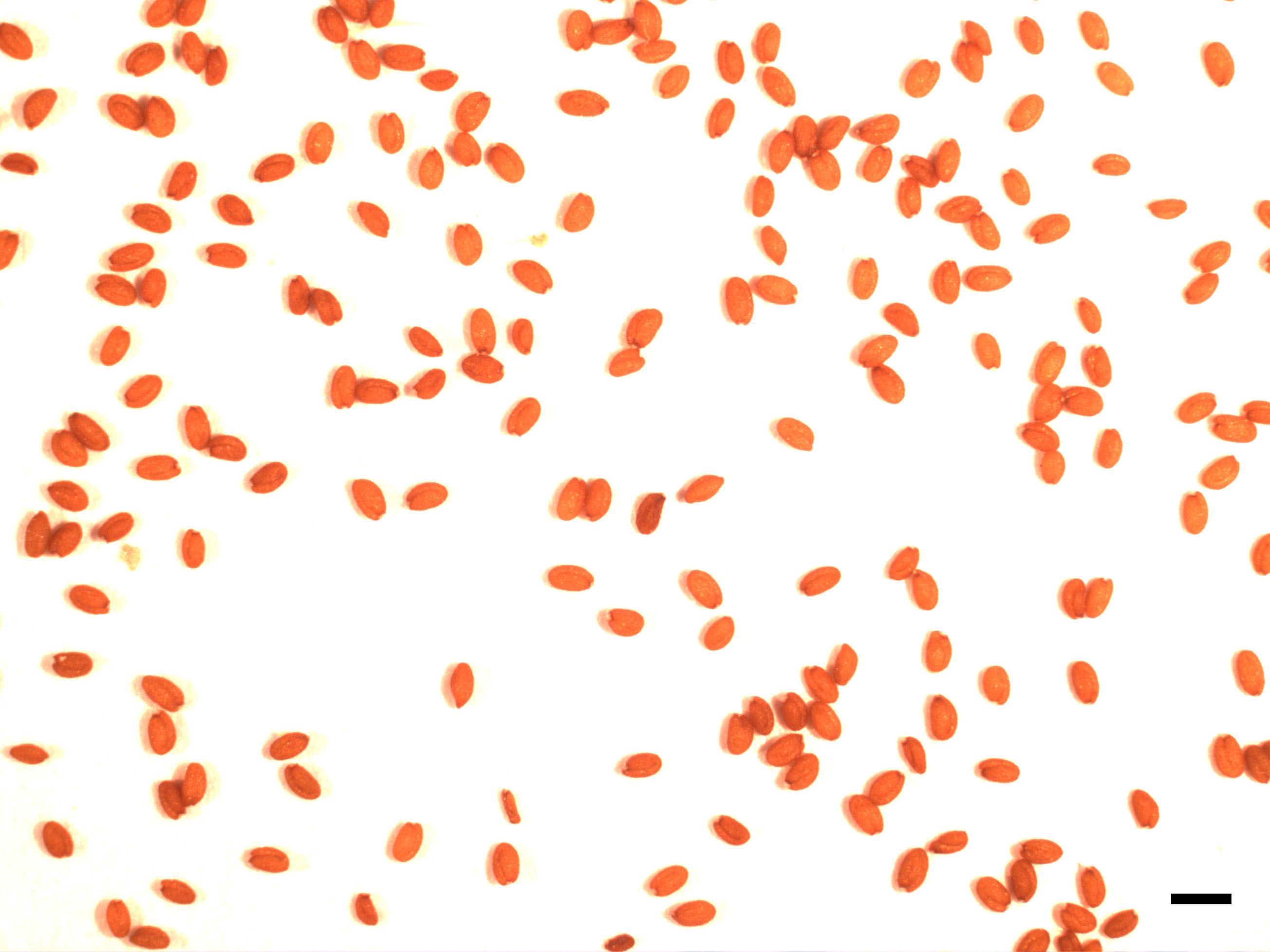

Supplement: Supplementary file 5 — Source Data [file 41467_2020_15603_MOESM5_ESM.zip › seed photos/35S=MYC-PPD1 #1/35S=MYC-PPD1 #1-1.jpg]

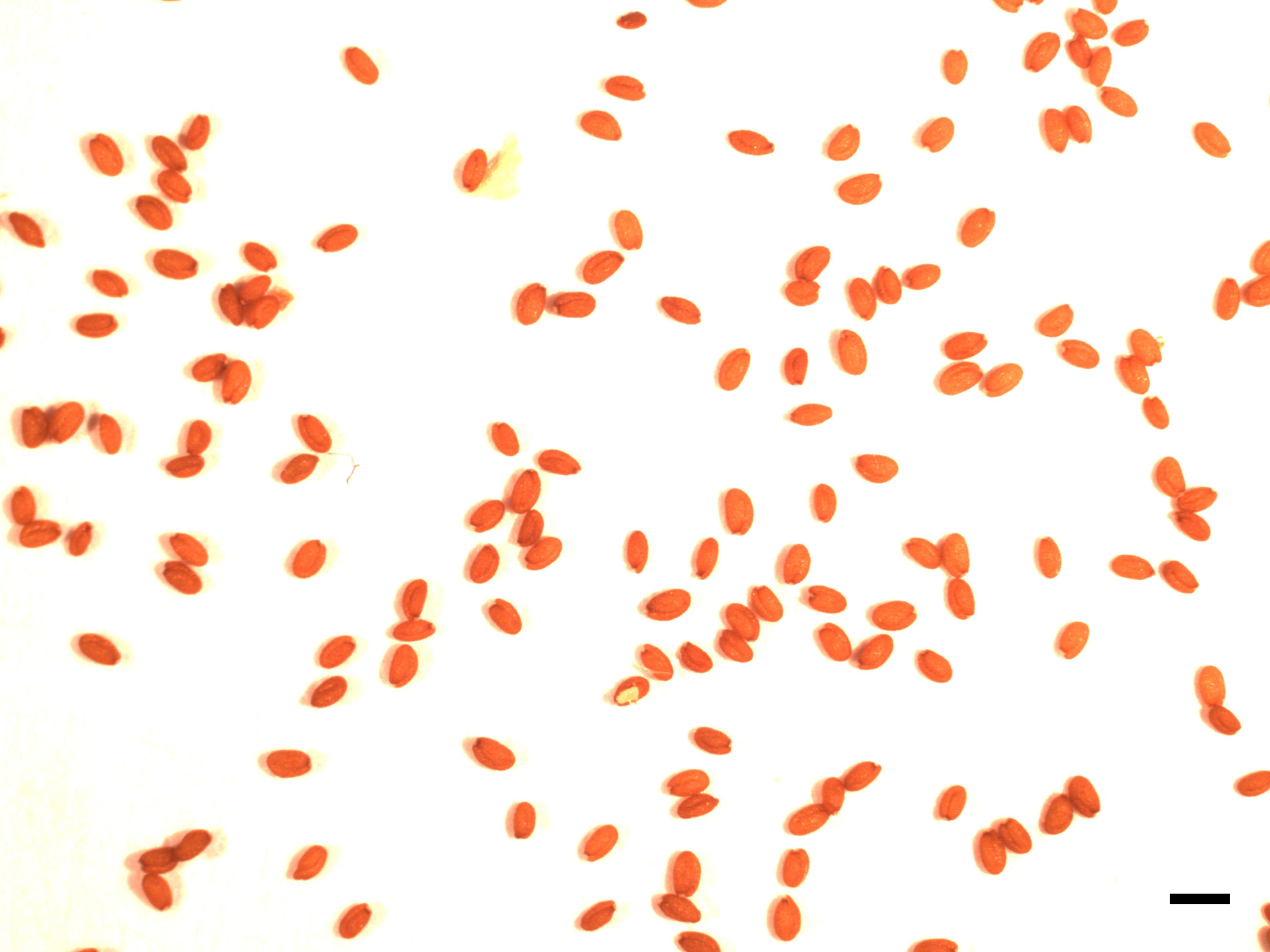

Supplement: Supplementary file 5 — Source Data [file 41467_2020_15603_MOESM5_ESM.zip › seed photos/35S=MYC-PPD1 #1/35S=MYC-PPD1 #1-2.jpg]

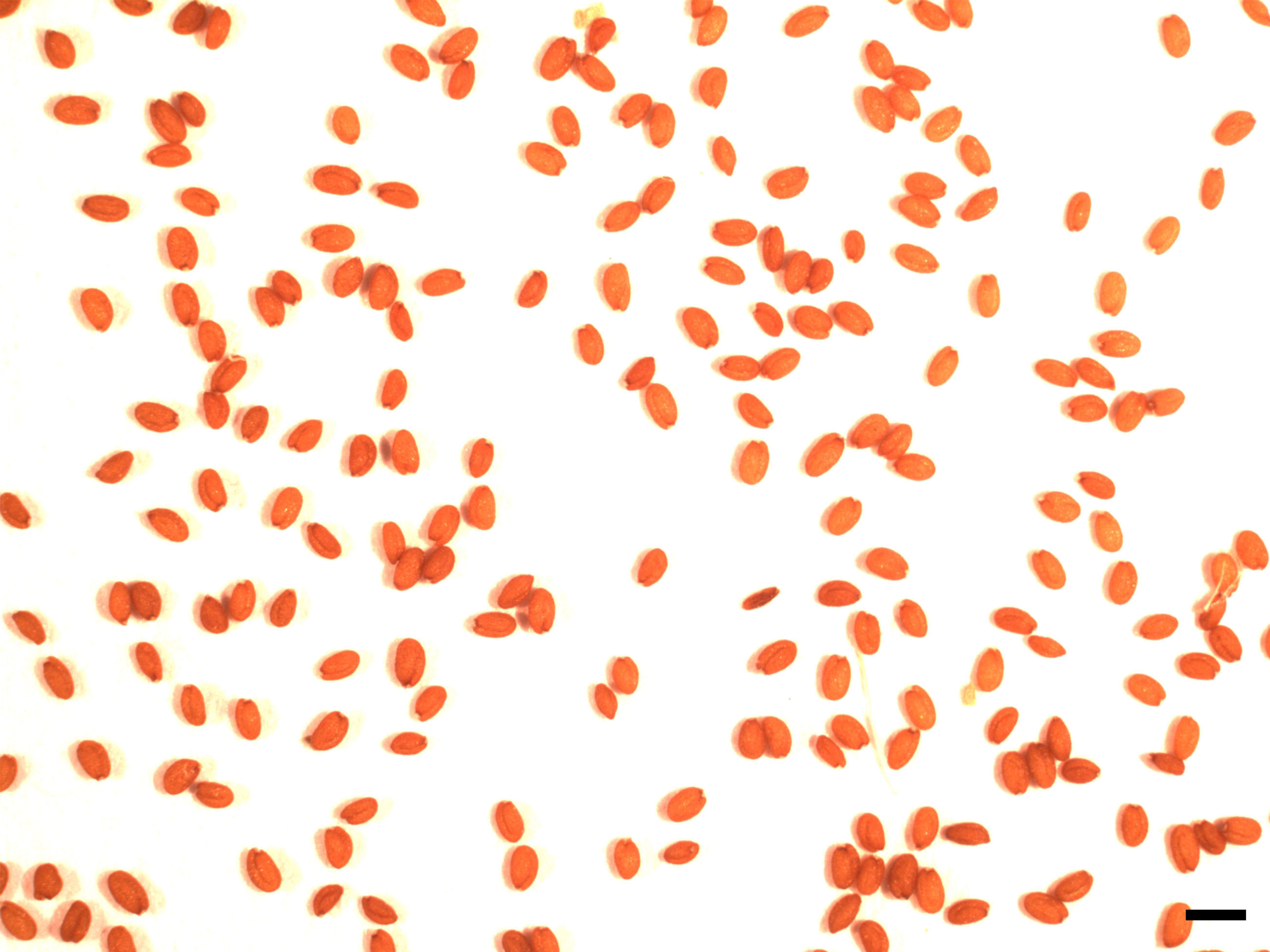

Supplement: Supplementary file 5 — Source Data [file 41467_2020_15603_MOESM5_ESM.zip › seed photos/35S=MYC-PPD1 #5/35S=MYC-PPD1 #5-1.jpg]

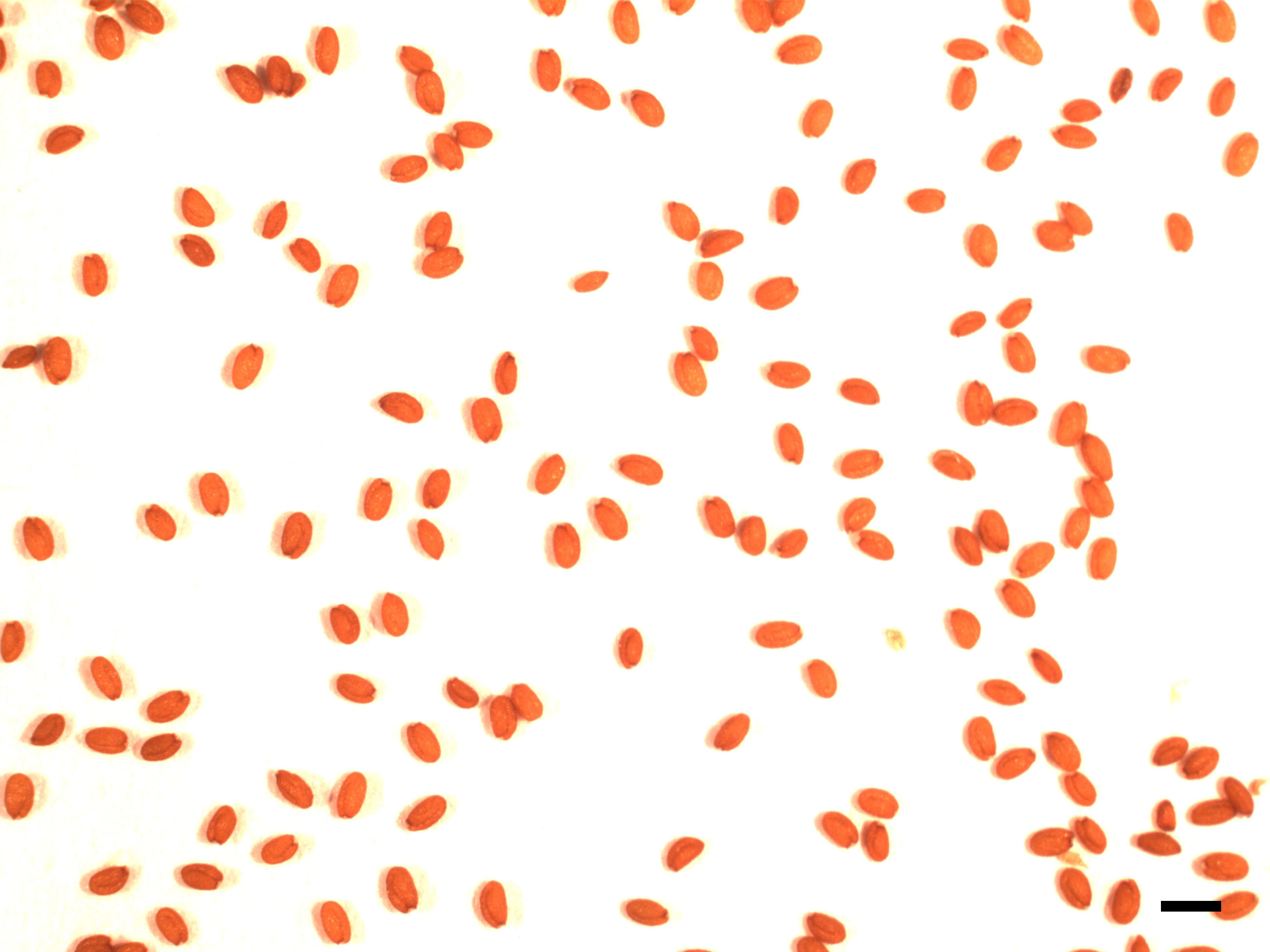

Supplement: Supplementary file 5 — Source Data [file 41467_2020_15603_MOESM5_ESM.zip › seed photos/35S=MYC-PPD1 #5/35S=MYC-PPD1 #5-2.jpg]

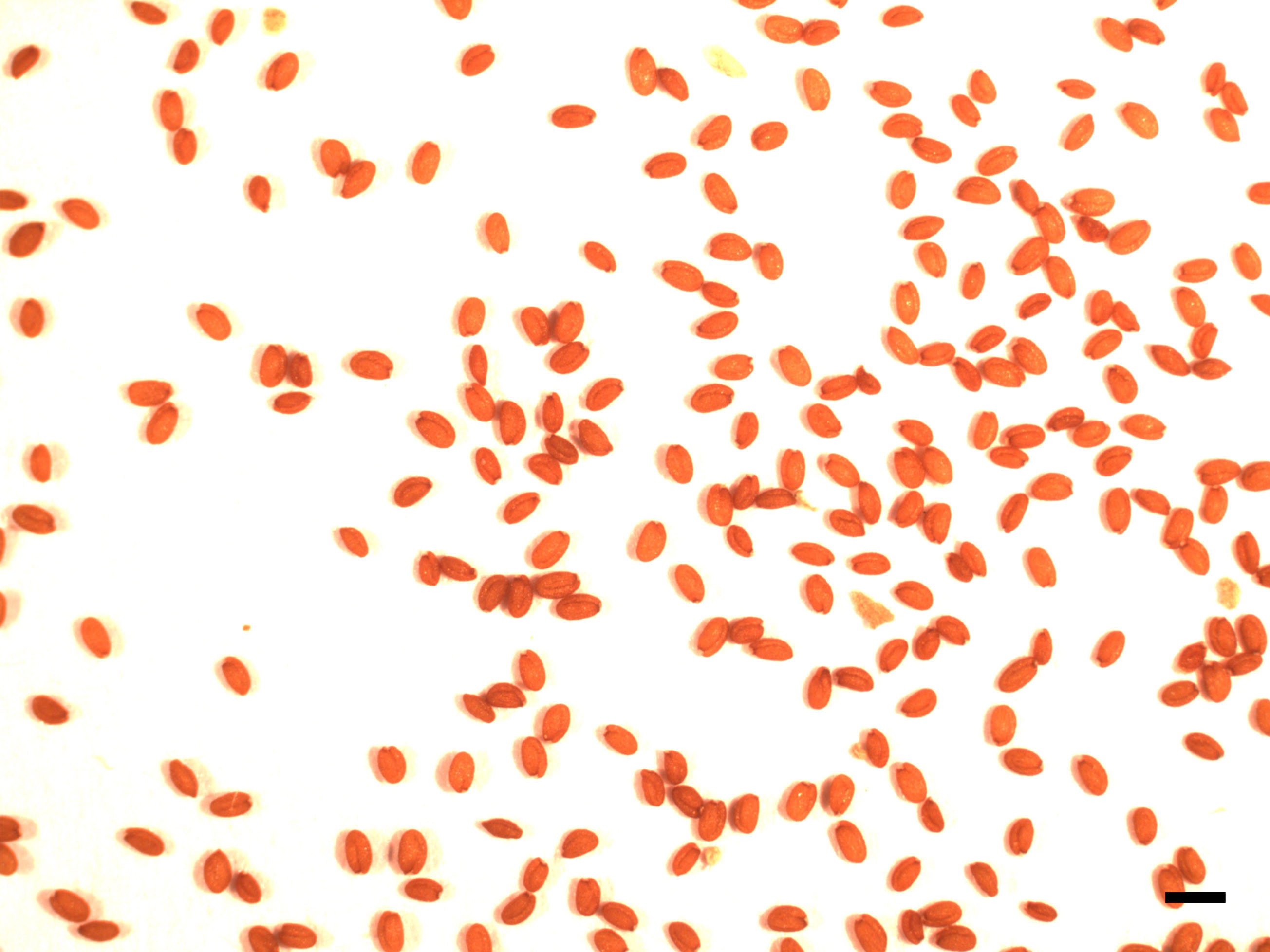

Supplement: Supplementary file 5 — Source Data [file 41467_2020_15603_MOESM5_ESM.zip › seed photos/35S=MYC-PPD1 #8/35S=MYC-PPD1 #8.jpg]

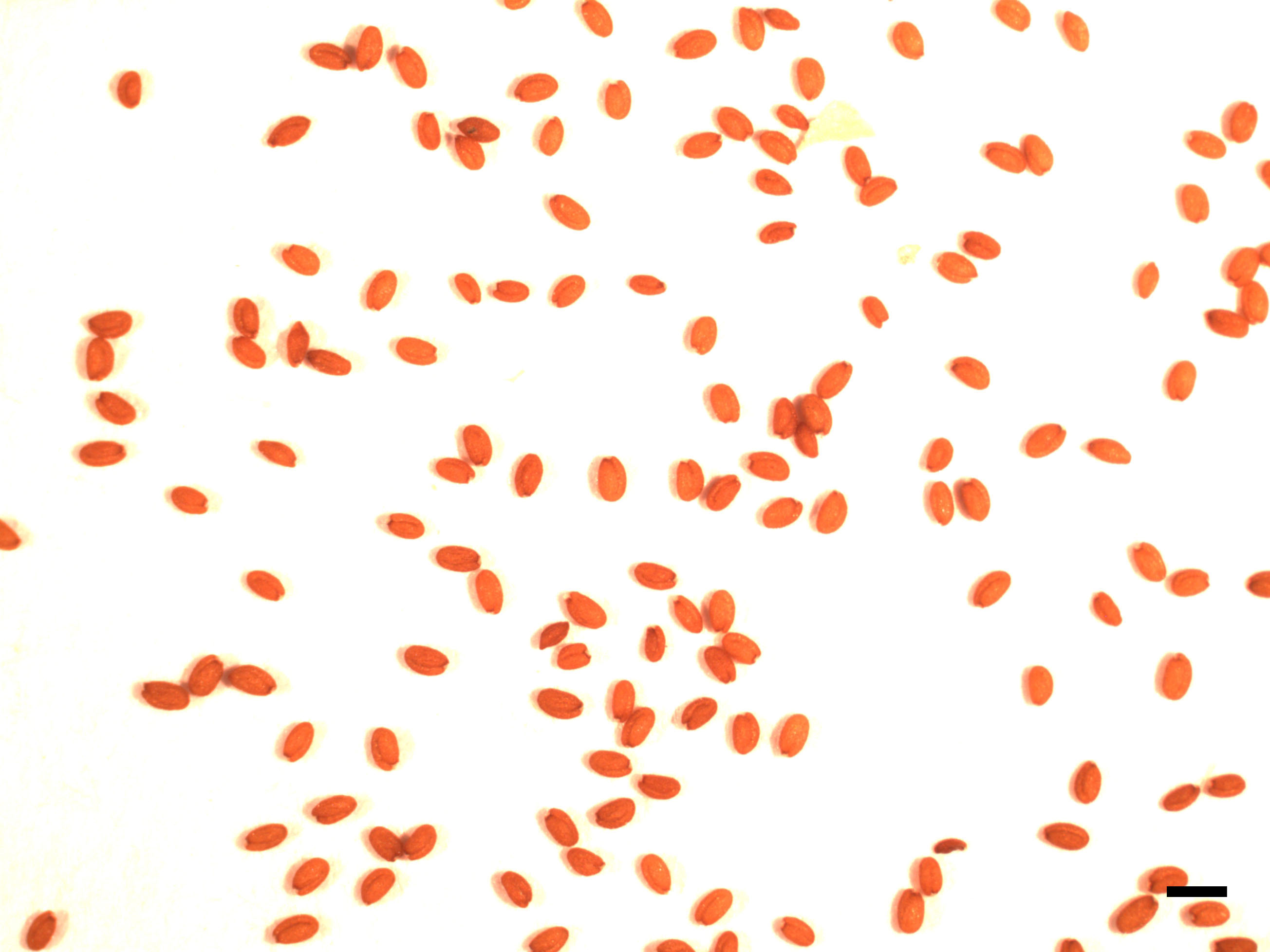

Supplement: Supplementary file 5 — Source Data [file 41467_2020_15603_MOESM5_ESM.zip › seed photos/35S=MYC-PPD2 #2/35S=MYC-PPD2 #2-1.jpg]

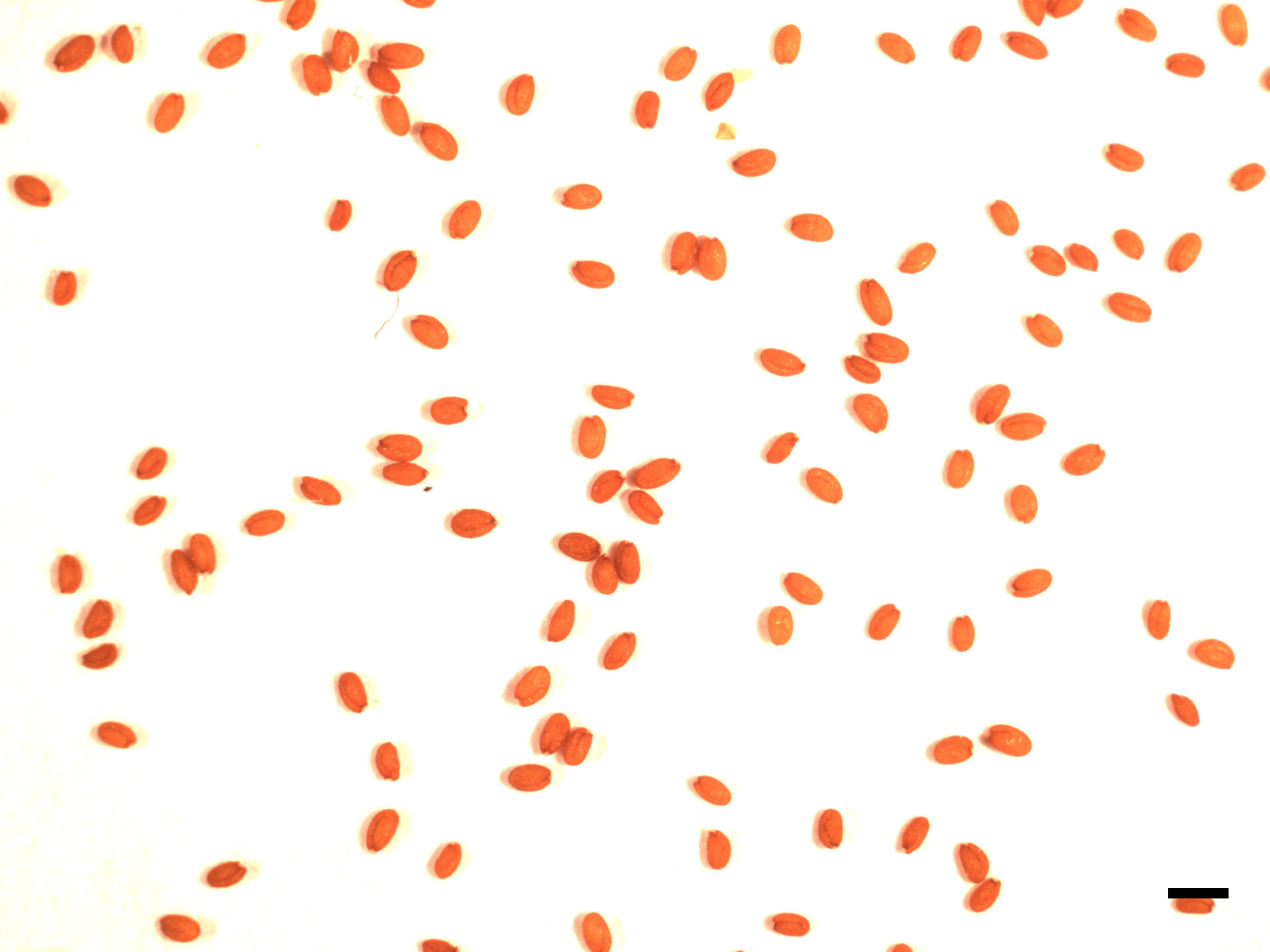

Supplement: Supplementary file 5 — Source Data [file 41467_2020_15603_MOESM5_ESM.zip › seed photos/35S=MYC-PPD2 #2/35S=MYC-PPD2 #2-2.jpg]

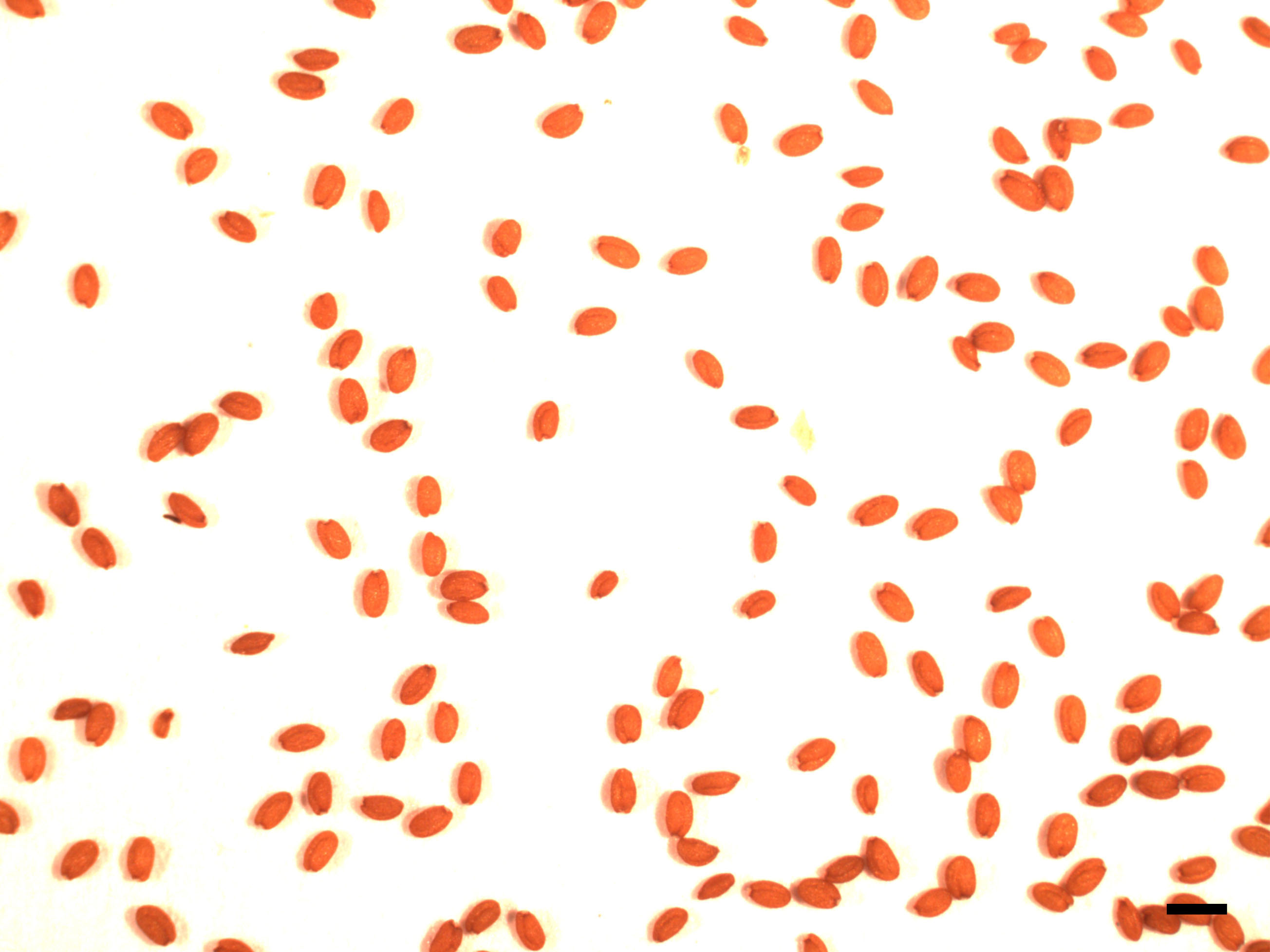

Supplement: Supplementary file 5 — Source Data [file 41467_2020_15603_MOESM5_ESM.zip › seed photos/35S=MYC-PPD2 #5/35S=MYC-PPD2 #5-1.jpg]

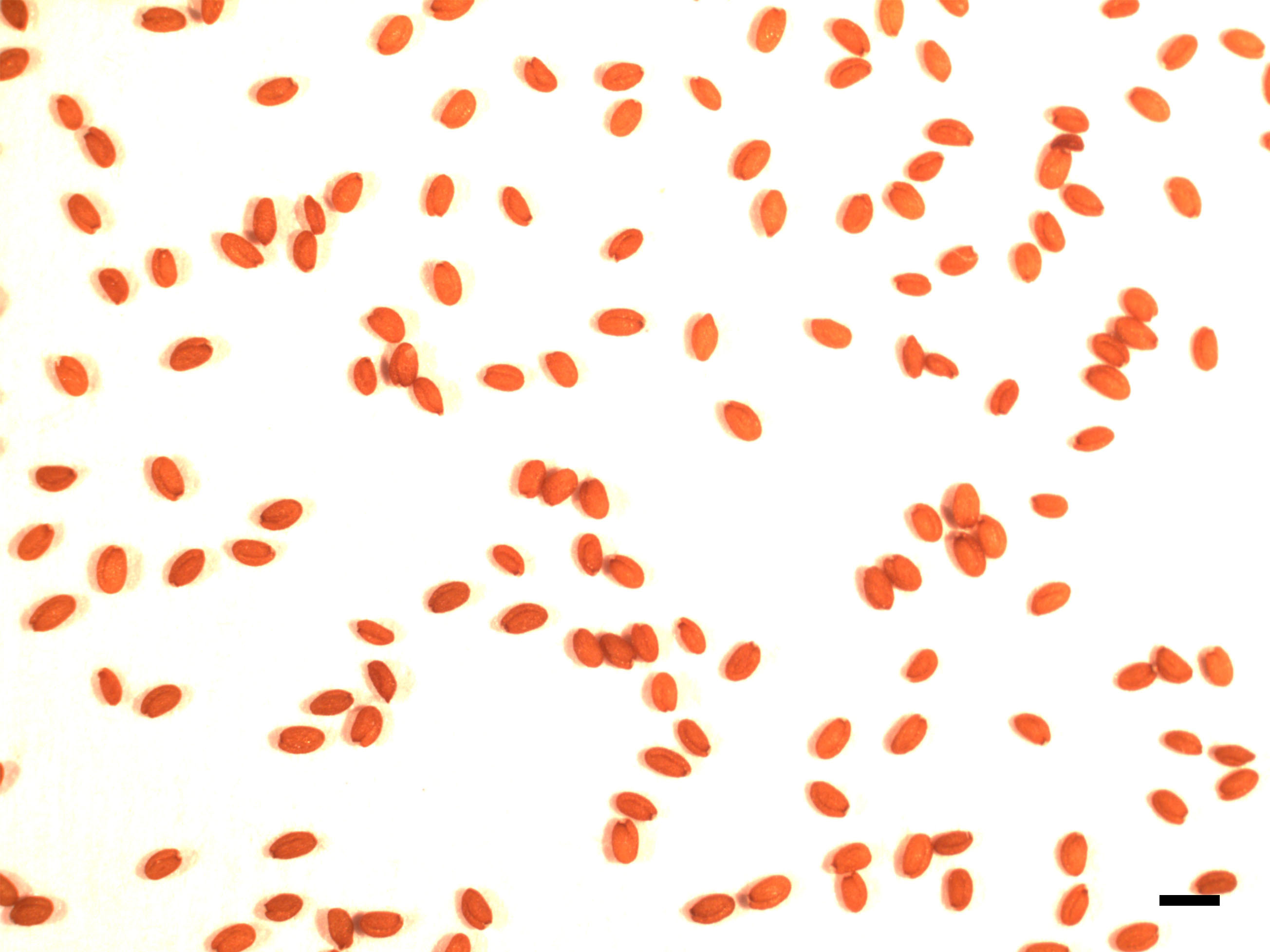

Supplement: Supplementary file 5 — Source Data [file 41467_2020_15603_MOESM5_ESM.zip › seed photos/35S=MYC-PPD2 #5/35S=MYC-PPD2 #5-2.jpg]

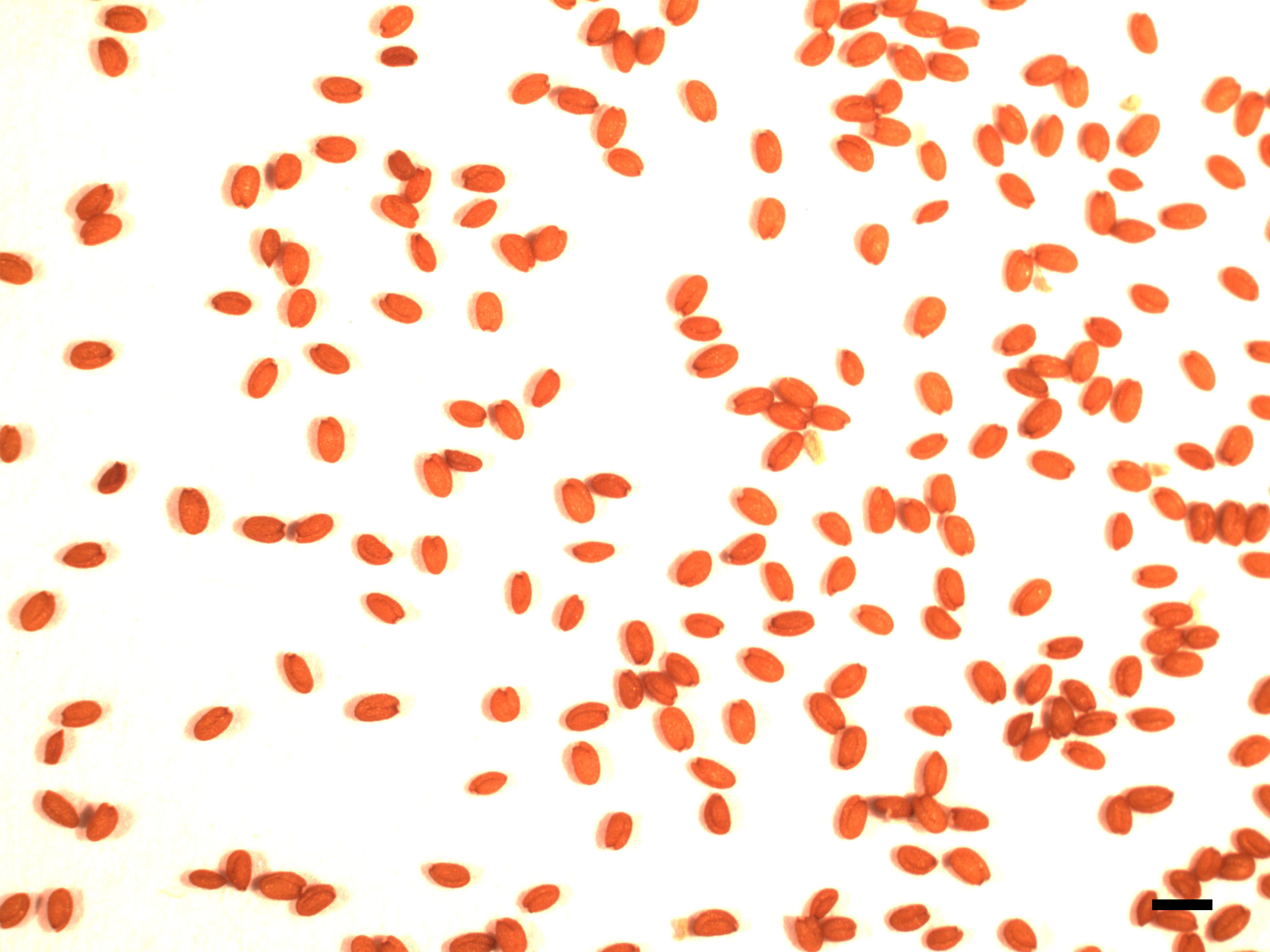

Supplement: Supplementary file 5 — Source Data [file 41467_2020_15603_MOESM5_ESM.zip › seed photos/35S=MYC-PPD2 #7/35S=MYC-PPD2 #7.jpg]

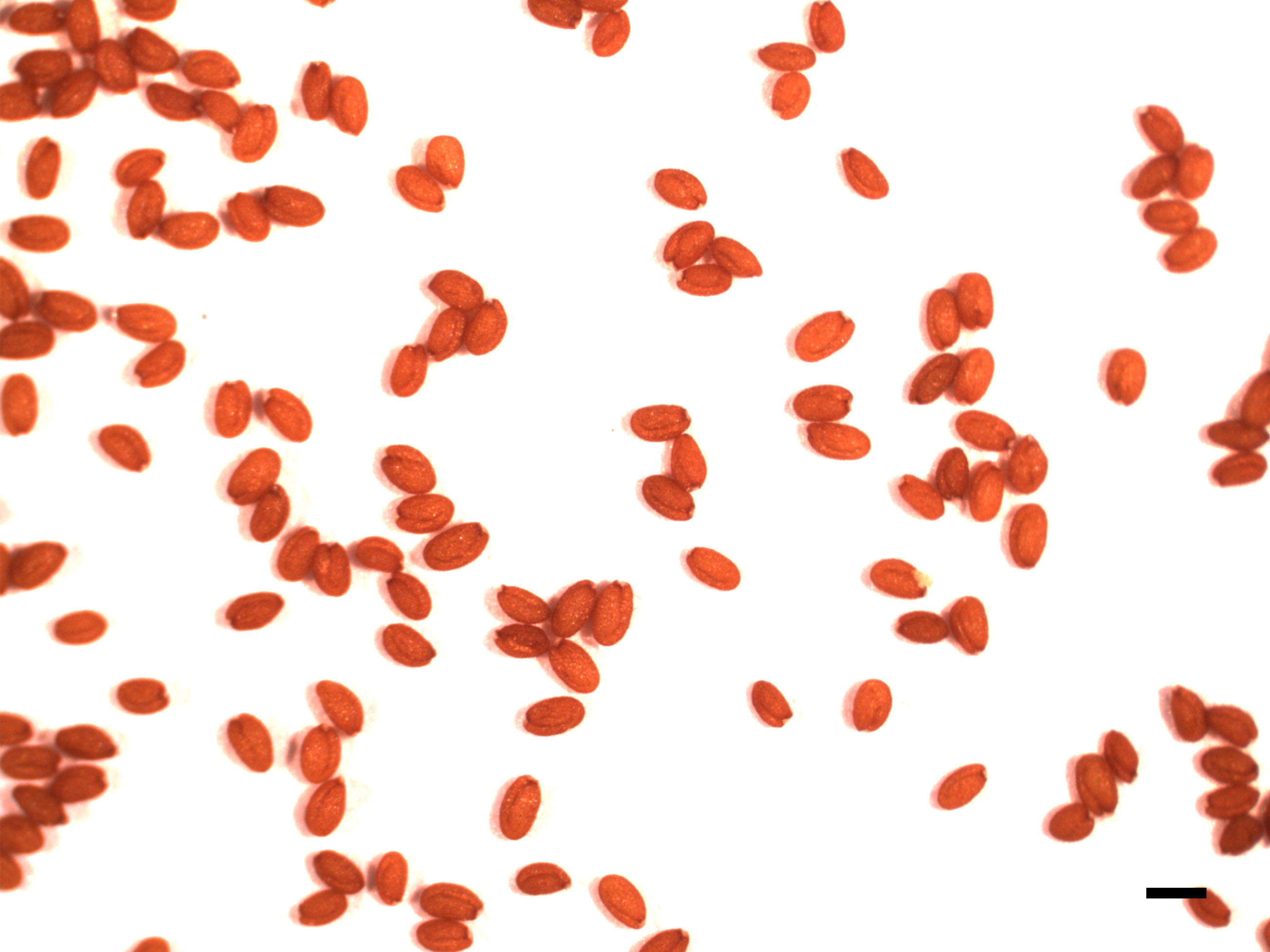

Supplement: Supplementary file 5 — Source Data [file 41467_2020_15603_MOESM5_ESM.zip › seed photos/35S=SAP/35S=SAP-1.jpg]

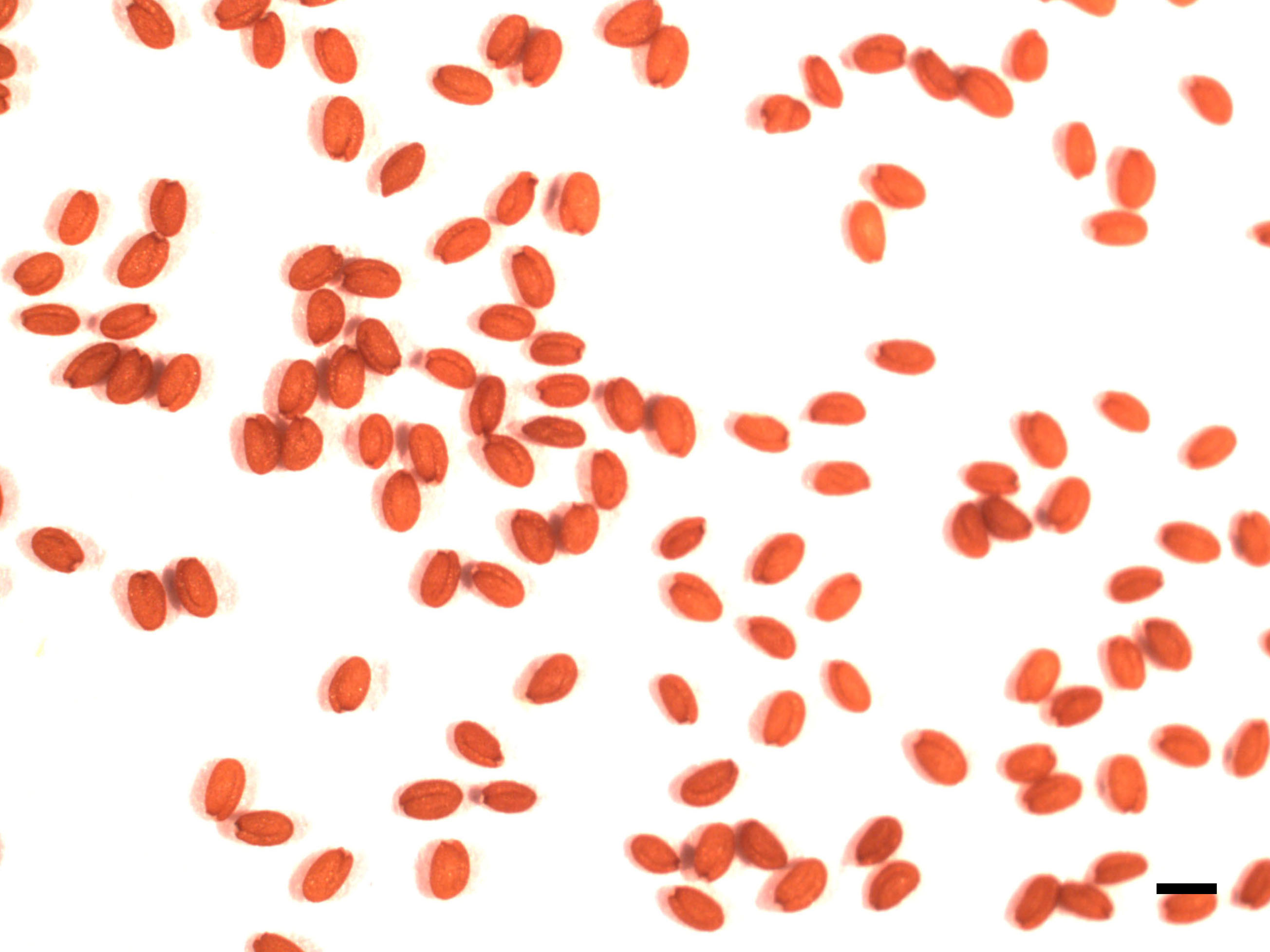

Supplement: Supplementary file 5 — Source Data [file 41467_2020_15603_MOESM5_ESM.zip › seed photos/35S=SAP/35S=SAP-2.jpg]

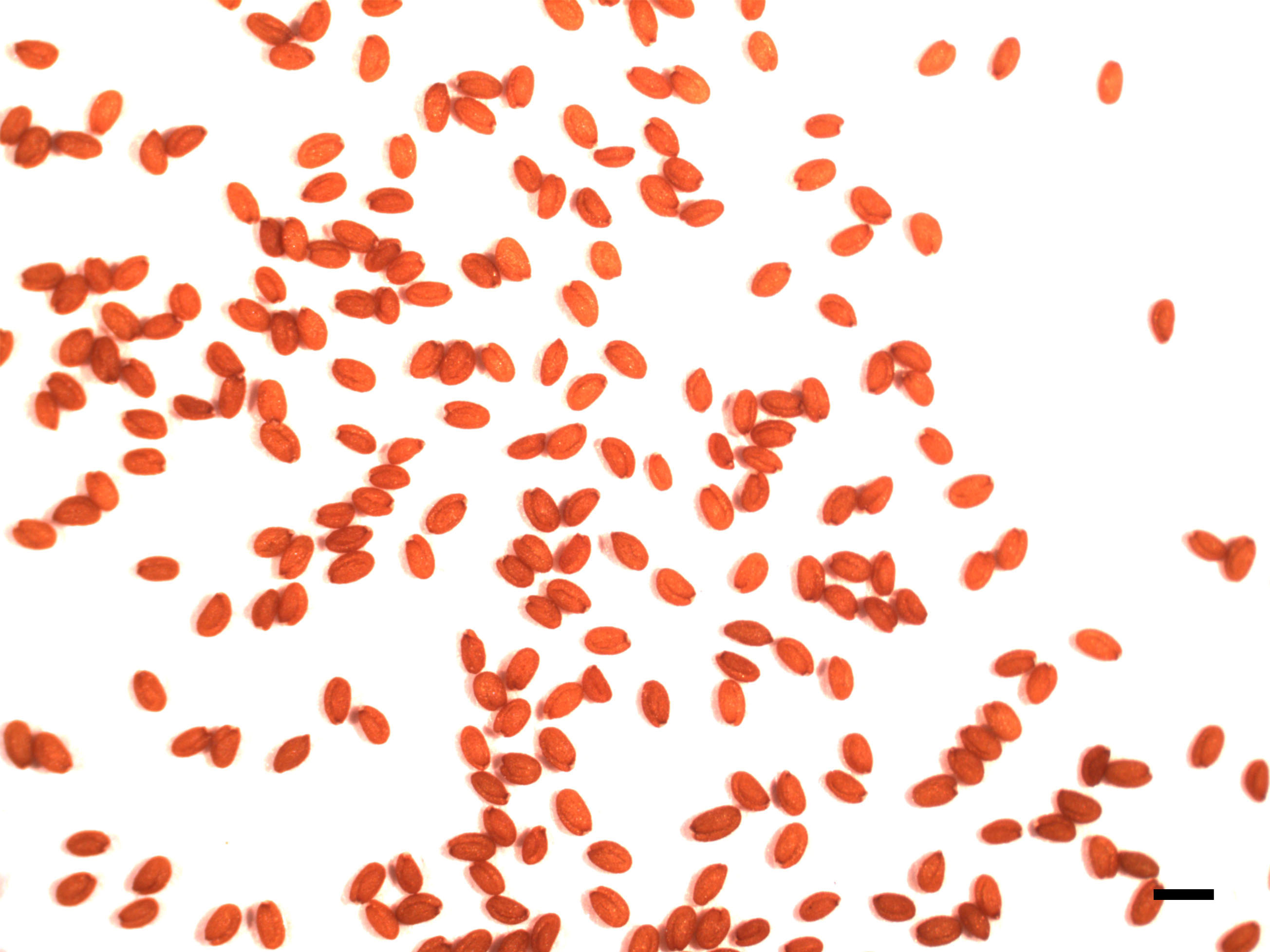

Supplement: Supplementary file 5 — Source Data [file 41467_2020_15603_MOESM5_ESM.zip › seed photos/35S=SAP;gif1/35S=SAP;gif1.jpg]

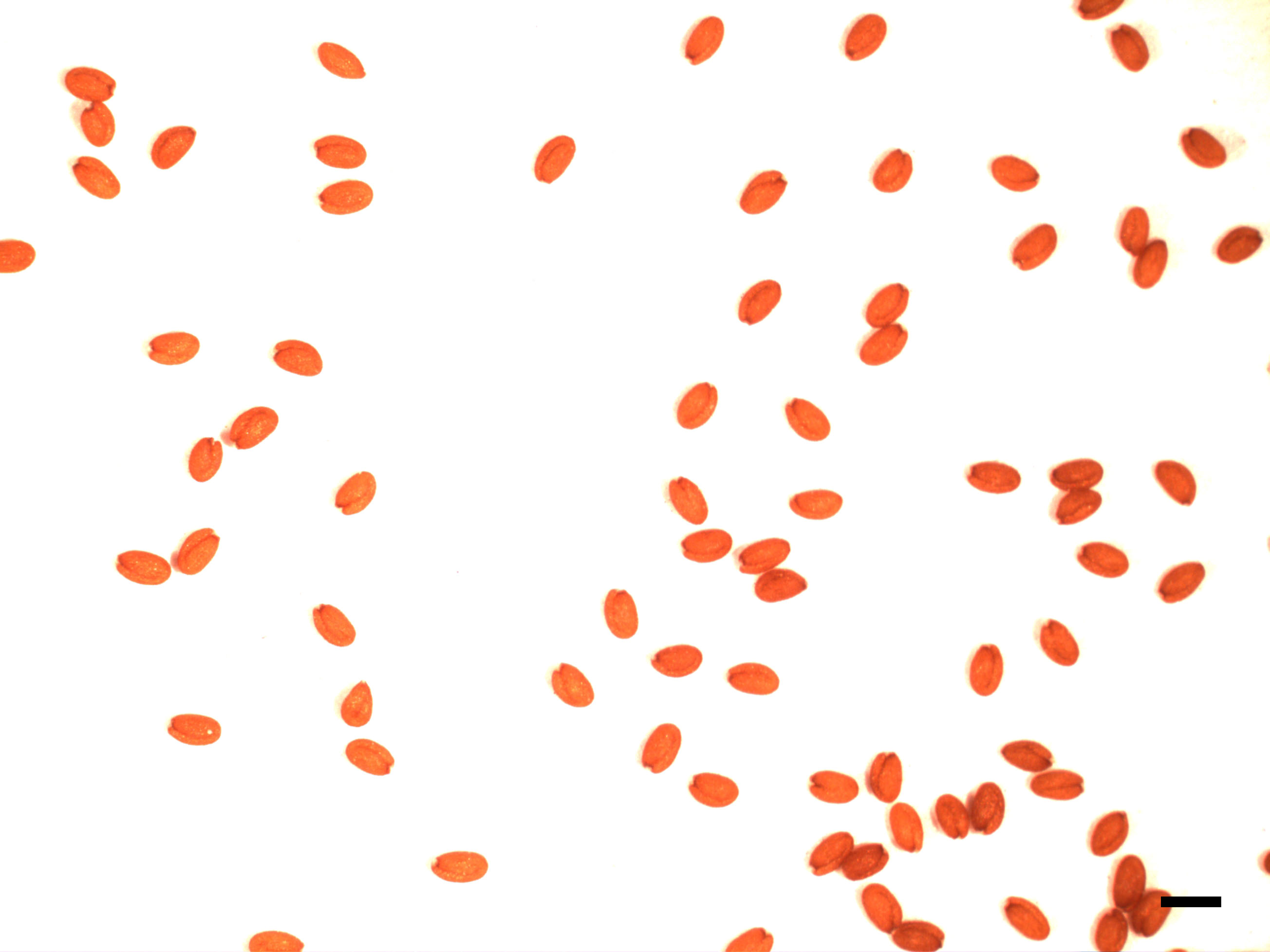

Supplement: Supplementary file 5 — Source Data [file 41467_2020_15603_MOESM5_ESM.zip › seed photos/Col-0/col-0 #1.jpg]

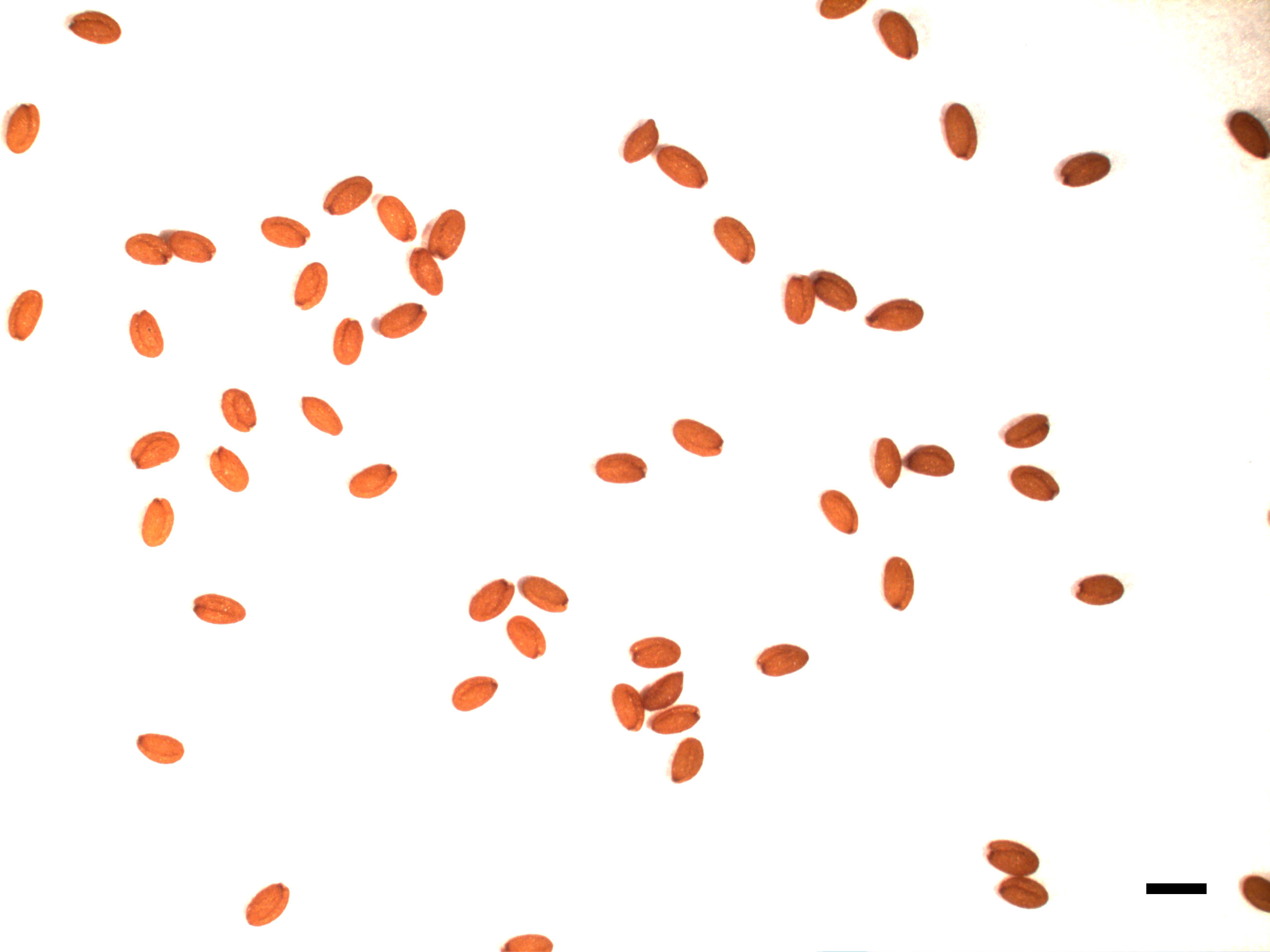

Supplement: Supplementary file 5 — Source Data [file 41467_2020_15603_MOESM5_ESM.zip › seed photos/Col-0/col-0 #2.jpg]

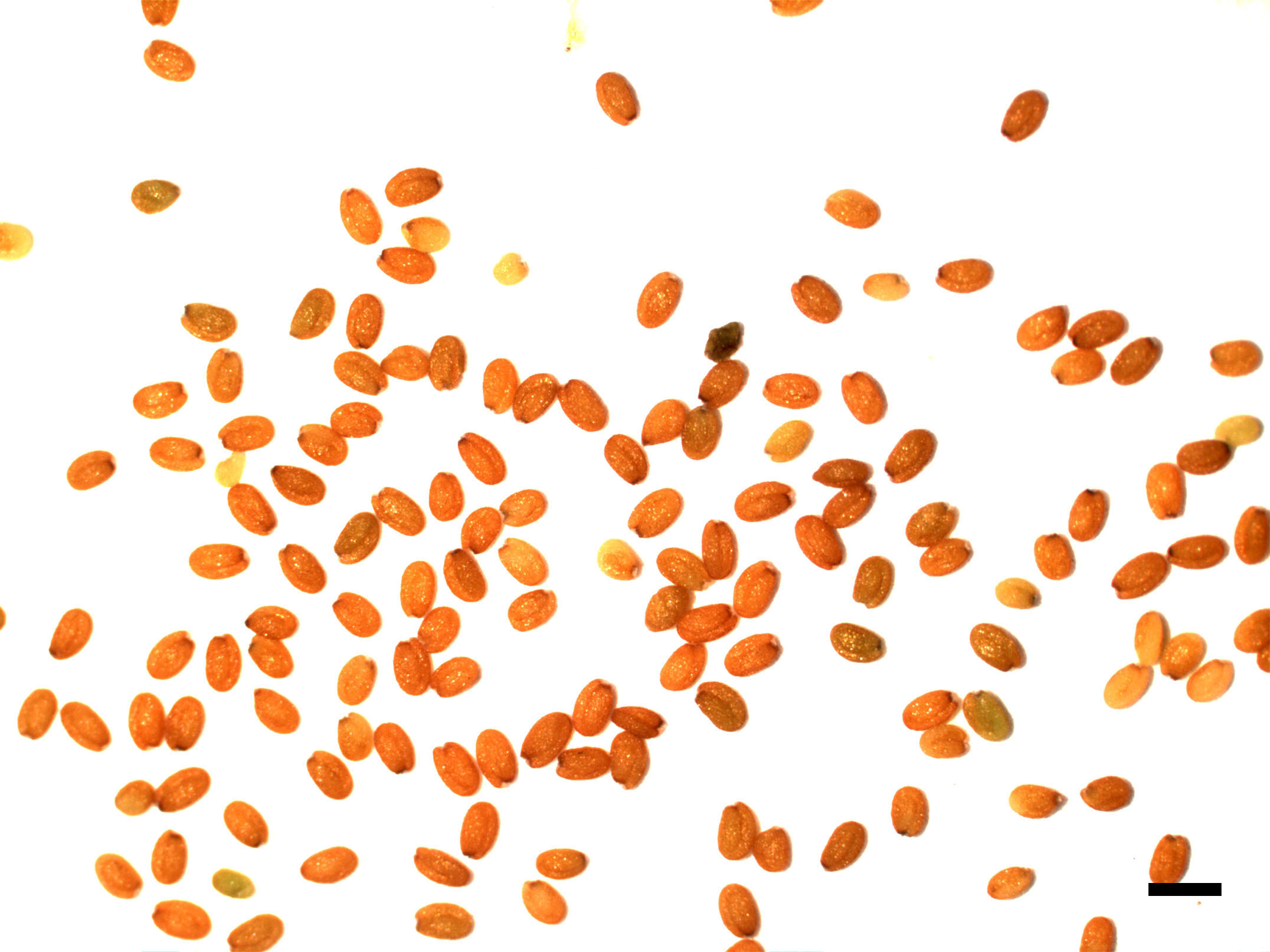

Supplement: Supplementary file 5 — Source Data [file 41467_2020_15603_MOESM5_ESM.zip › seed photos/gif1 kix8-1 kix9-1/gif1 kix8-1 kix9-1.jpg]

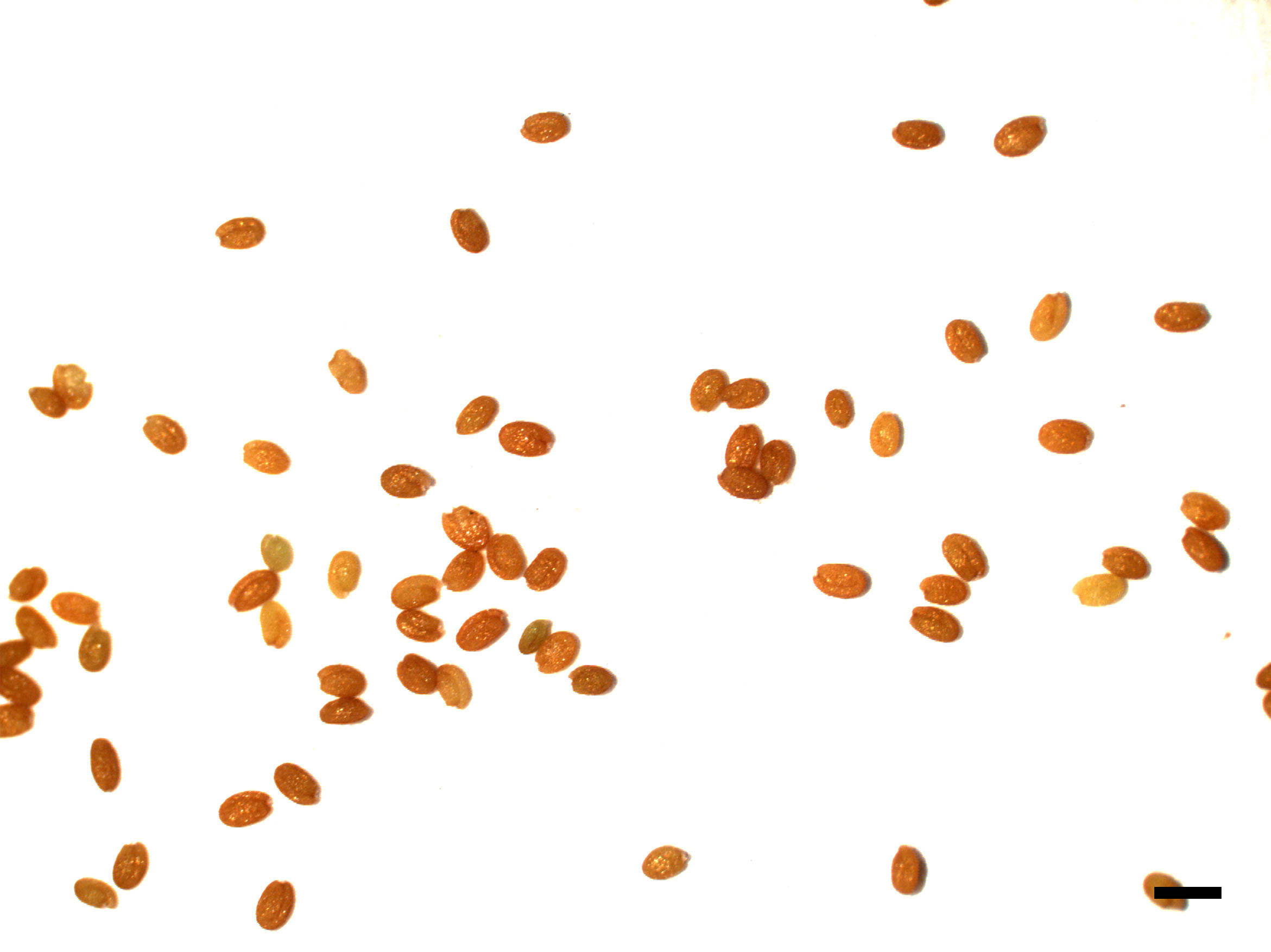

Supplement: Supplementary file 5 — Source Data [file 41467_2020_15603_MOESM5_ESM.zip › seed photos/gif1 myc3 myc4/gif1 myc3 myc4 #1.jpg]

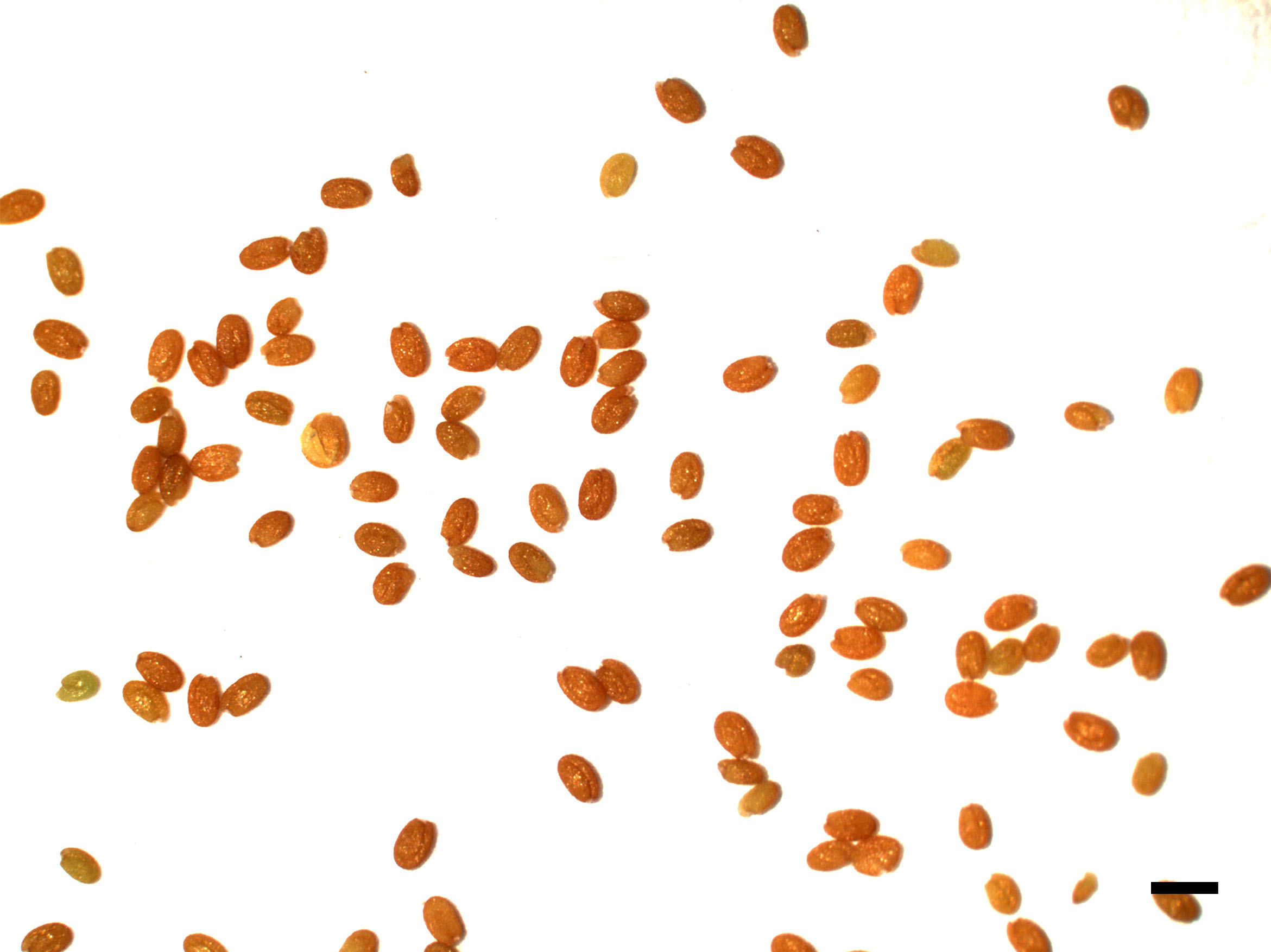

Supplement: Supplementary file 5 — Source Data [file 41467_2020_15603_MOESM5_ESM.zip › seed photos/gif1 myc3 myc4/gif1 myc3 myc4 #2.jpg]

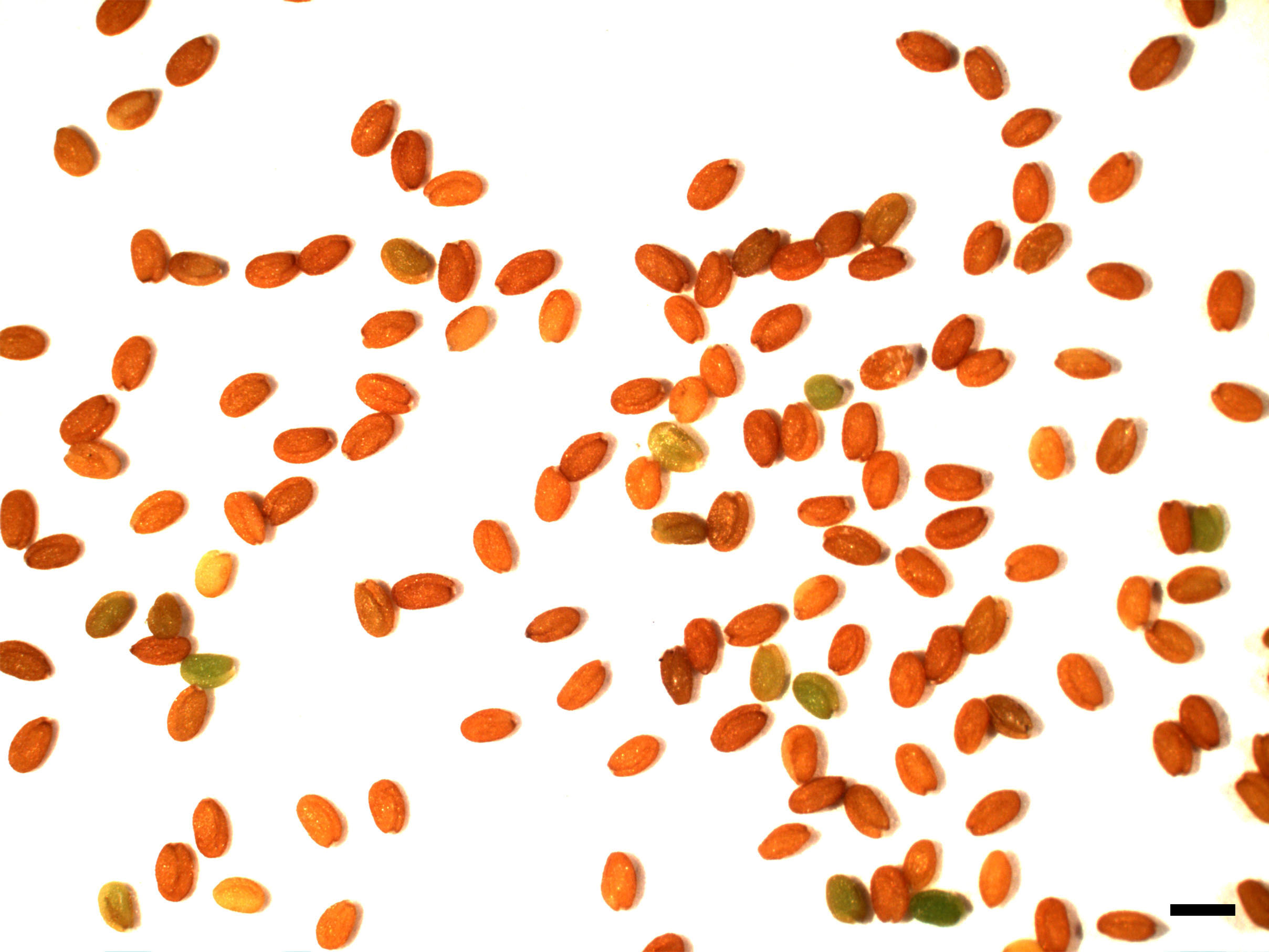

Supplement: Supplementary file 5 — Source Data [file 41467_2020_15603_MOESM5_ESM.zip › seed photos/gif1 ppd1-2 ppd2-cr/gif1 ppd1-2 ppd2-cr.jpg]

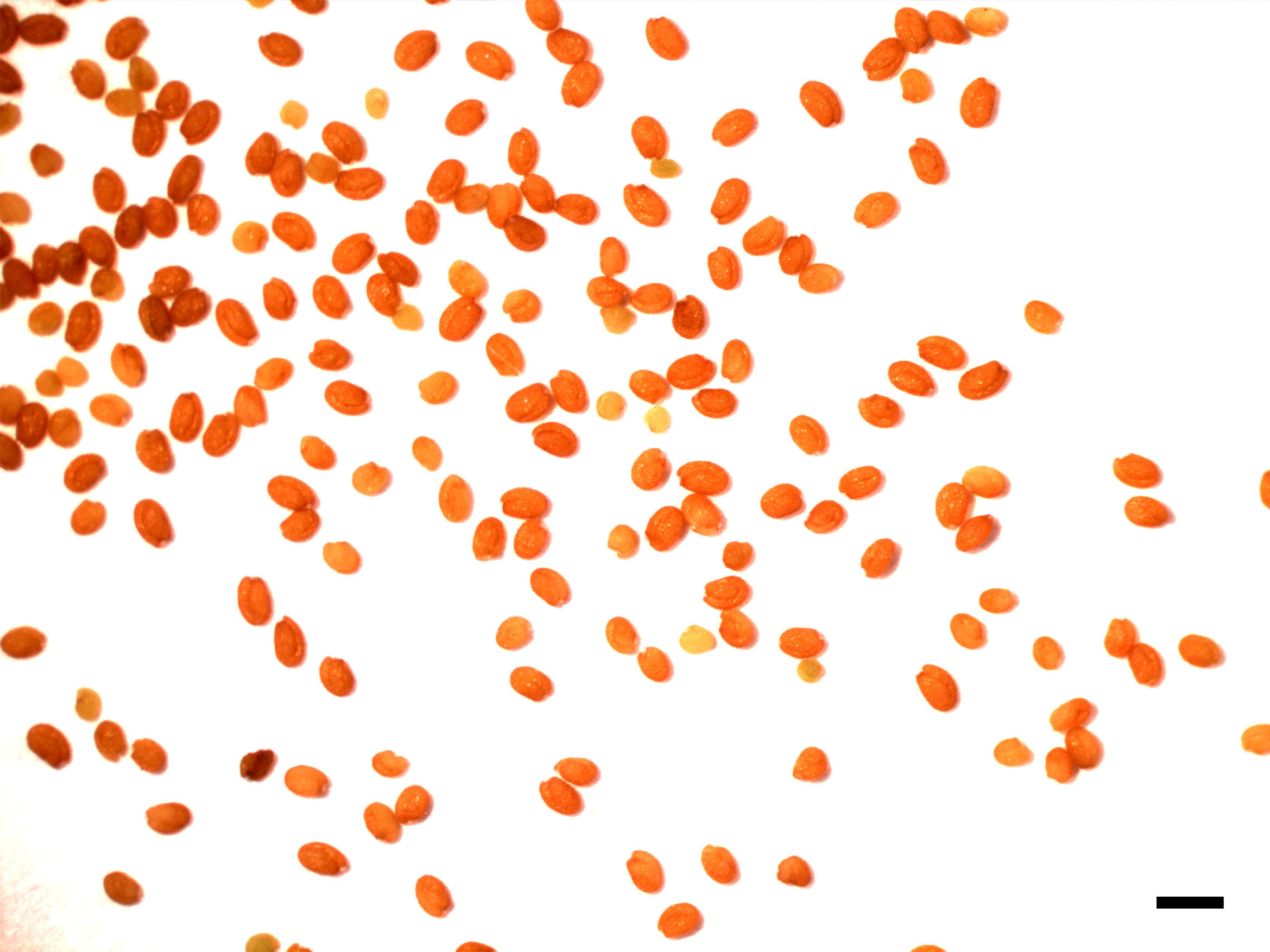

Supplement: Supplementary file 5 — Source Data [file 41467_2020_15603_MOESM5_ESM.zip › seed photos/gif1/gif1.jpg]

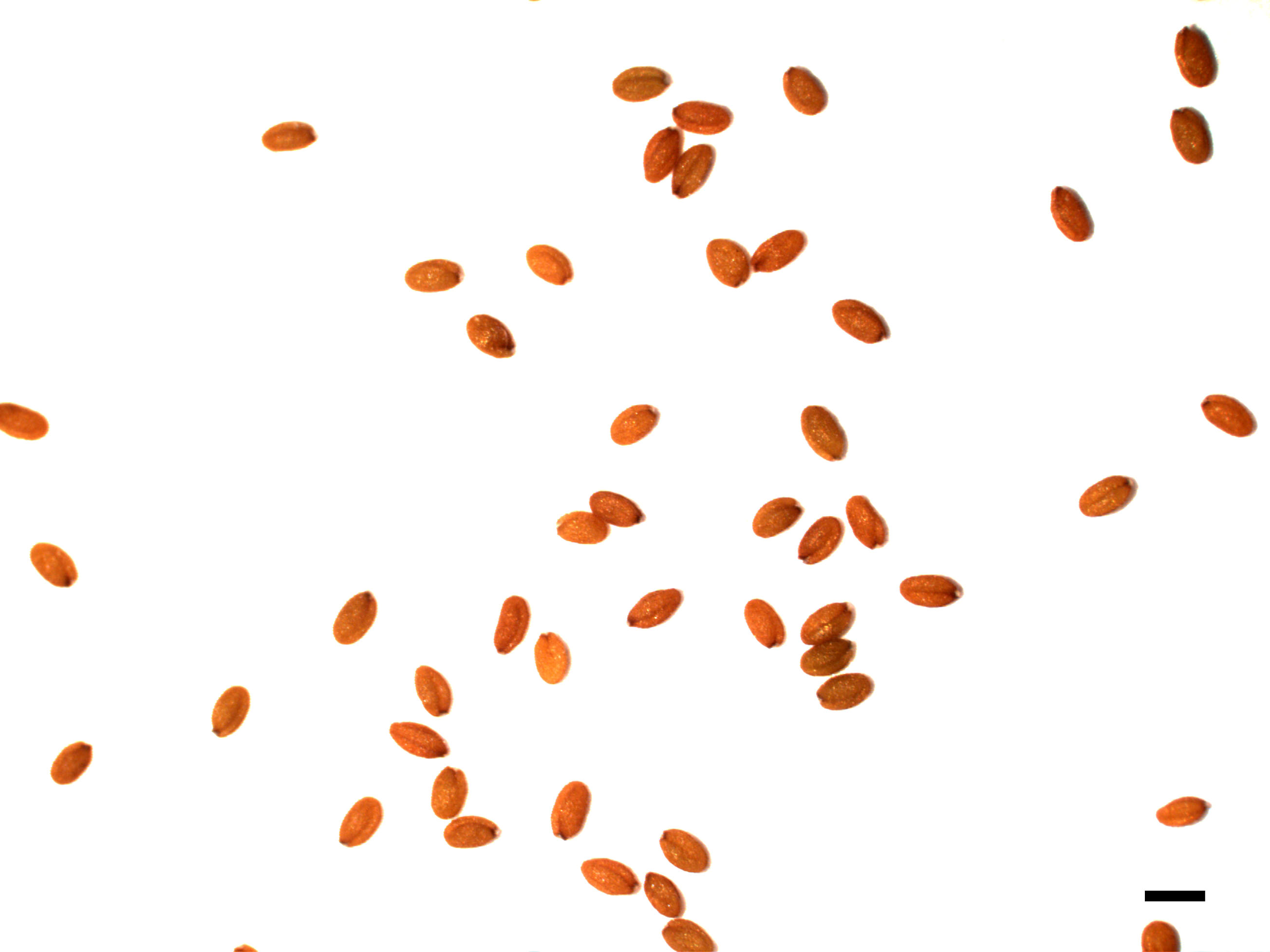

Supplement: Supplementary file 5 — Source Data [file 41467_2020_15603_MOESM5_ESM.zip › seed photos/kix8-1 kix9-1 ppd1-2 ppd2-cr/kix8-1 kix9-1 ppd1-2 ppd2-cr #1.jpg]

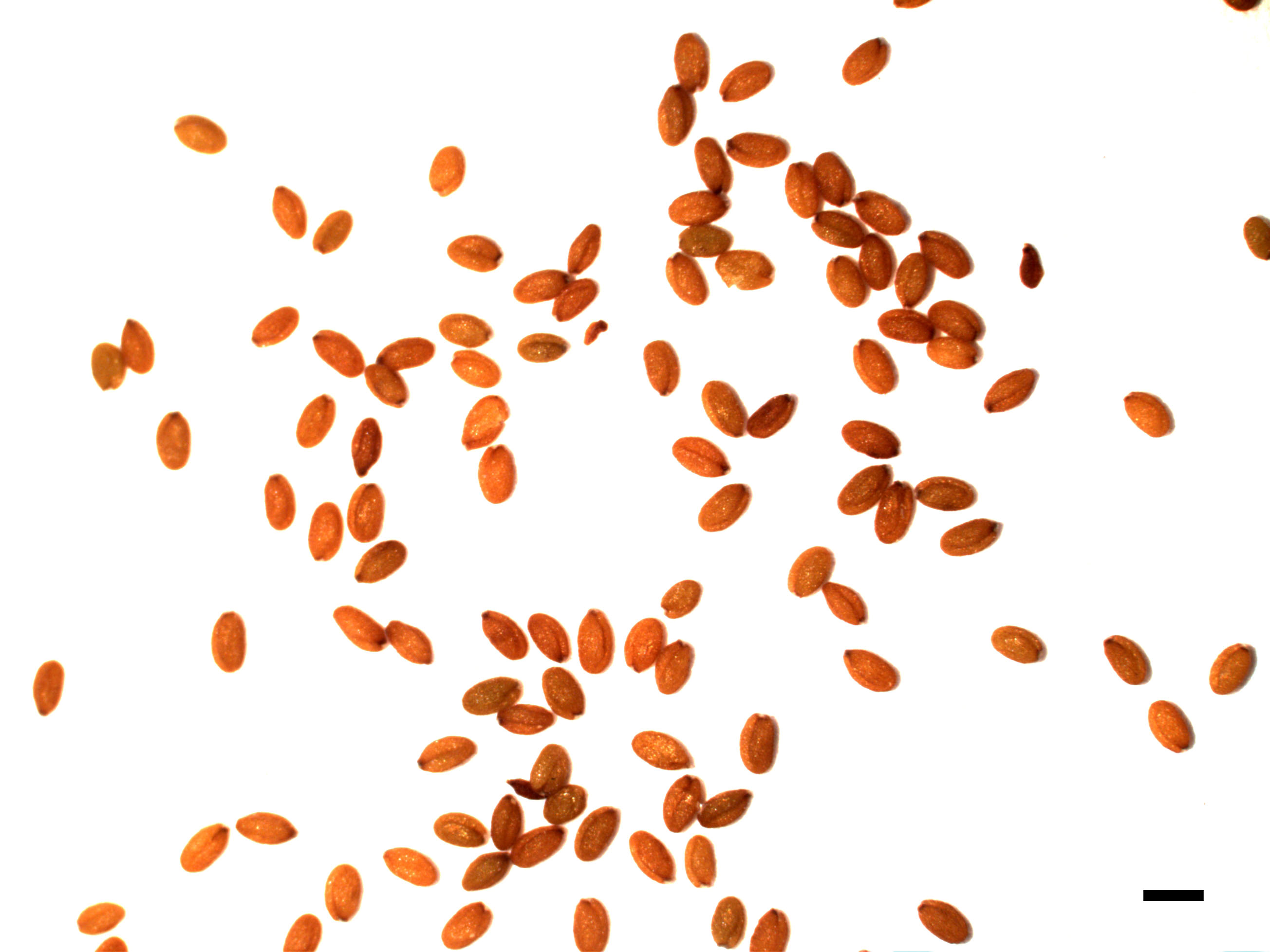

Supplement: Supplementary file 5 — Source Data [file 41467_2020_15603_MOESM5_ESM.zip › seed photos/kix8-1 kix9-1 ppd1-2 ppd2-cr/kix8-1 kix9-1 ppd1-2 ppd2-cr #2.jpg]

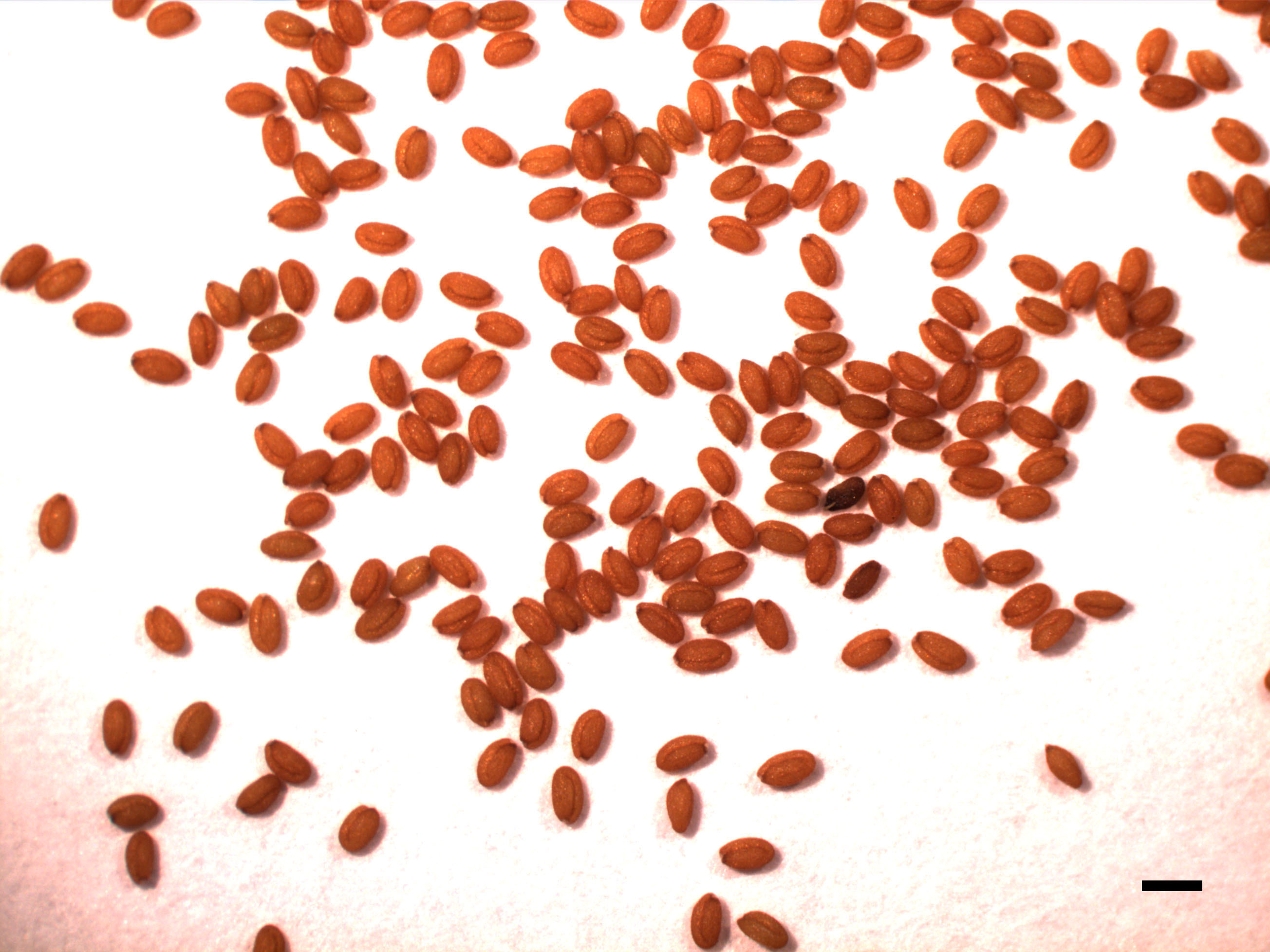

Supplement: Supplementary file 5 — Source Data [file 41467_2020_15603_MOESM5_ESM.zip › seed photos/kix8-1/kix8-1.jpg]

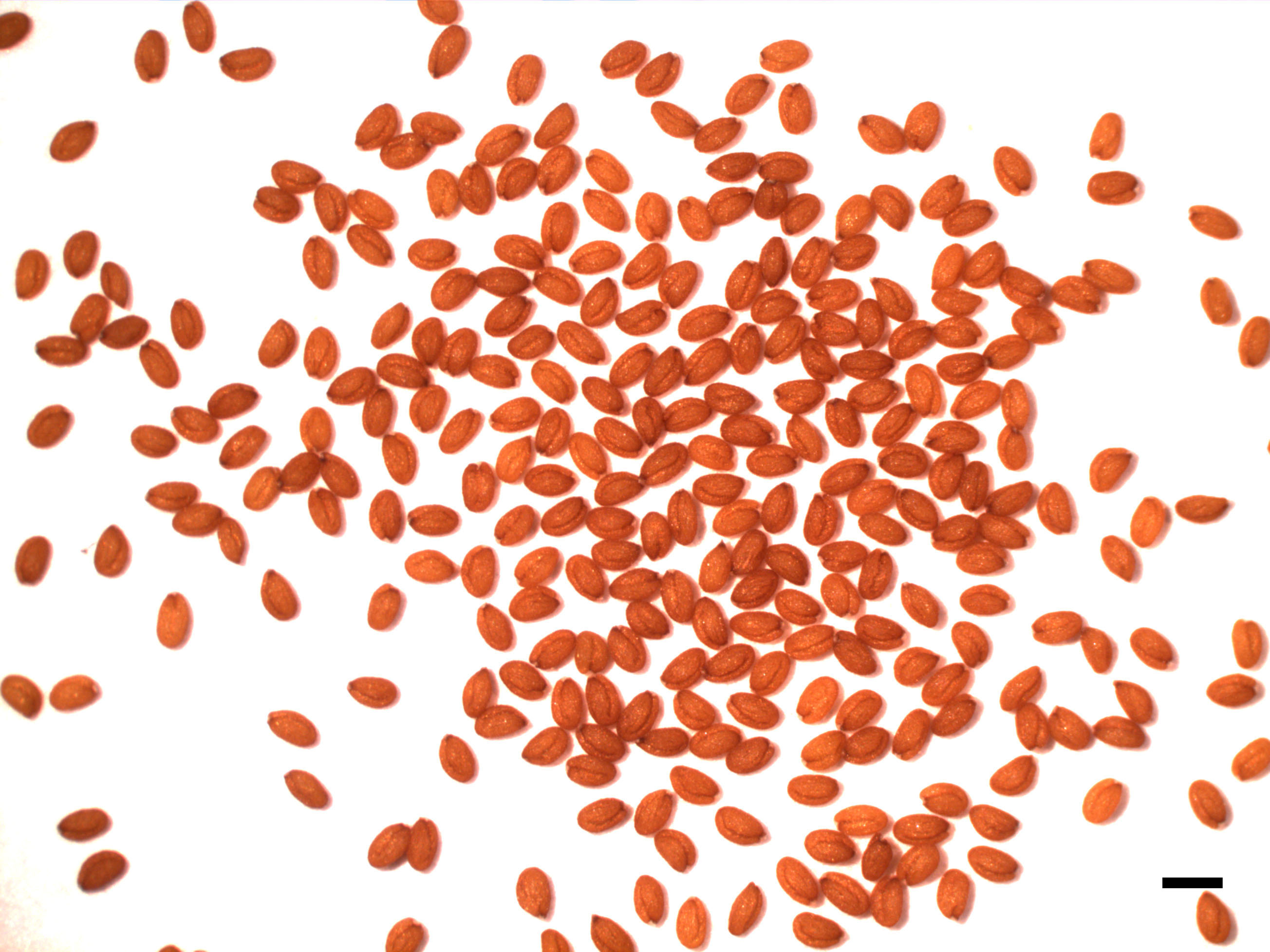

Supplement: Supplementary file 5 — Source Data [file 41467_2020_15603_MOESM5_ESM.zip › seed photos/Kix9-1/kix9-1.jpg]

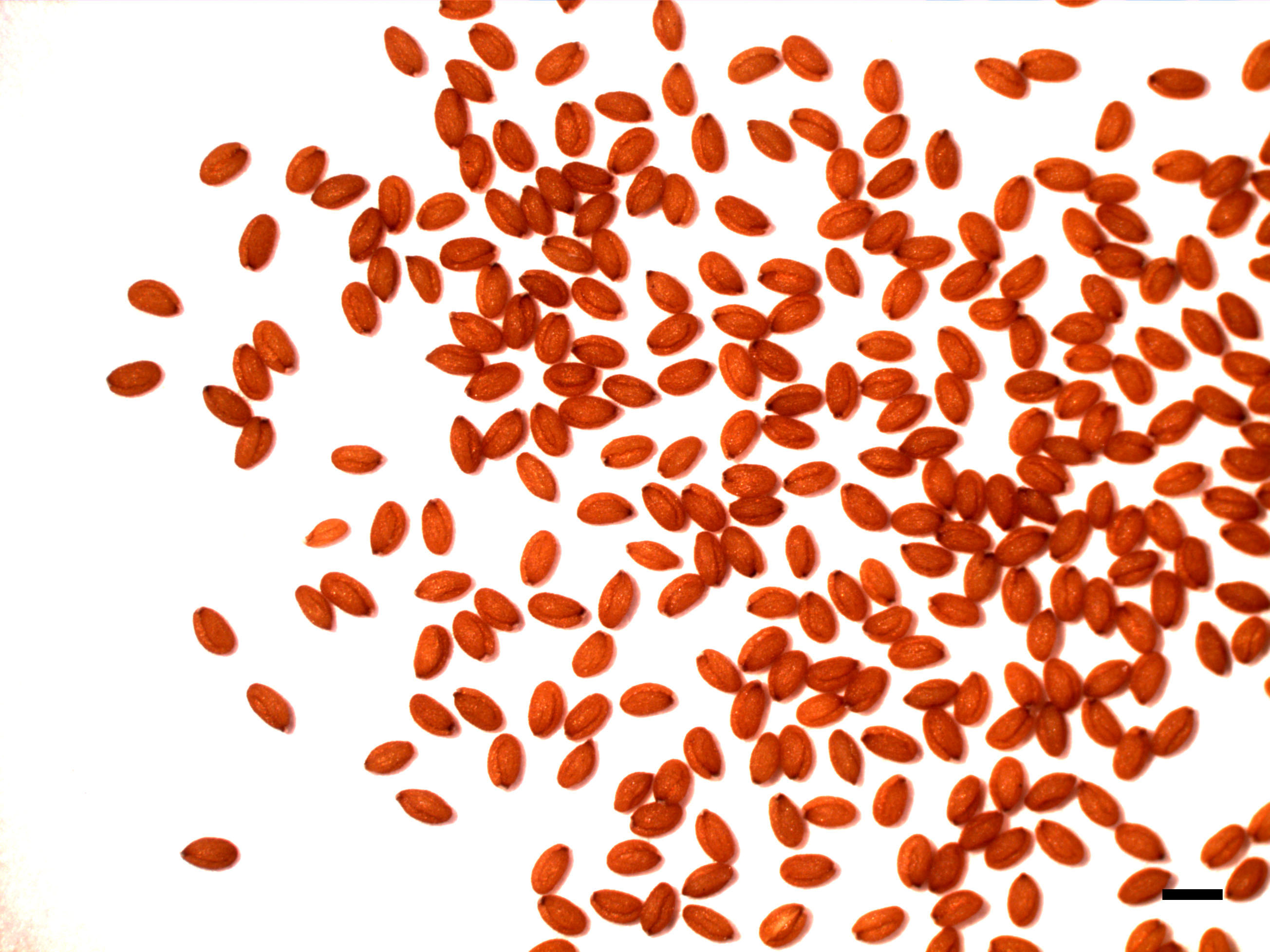

Supplement: Supplementary file 5 — Source Data [file 41467_2020_15603_MOESM5_ESM.zip › seed photos/kxi8-1 kix9-1/kxi8-1 kix9-1.jpg]

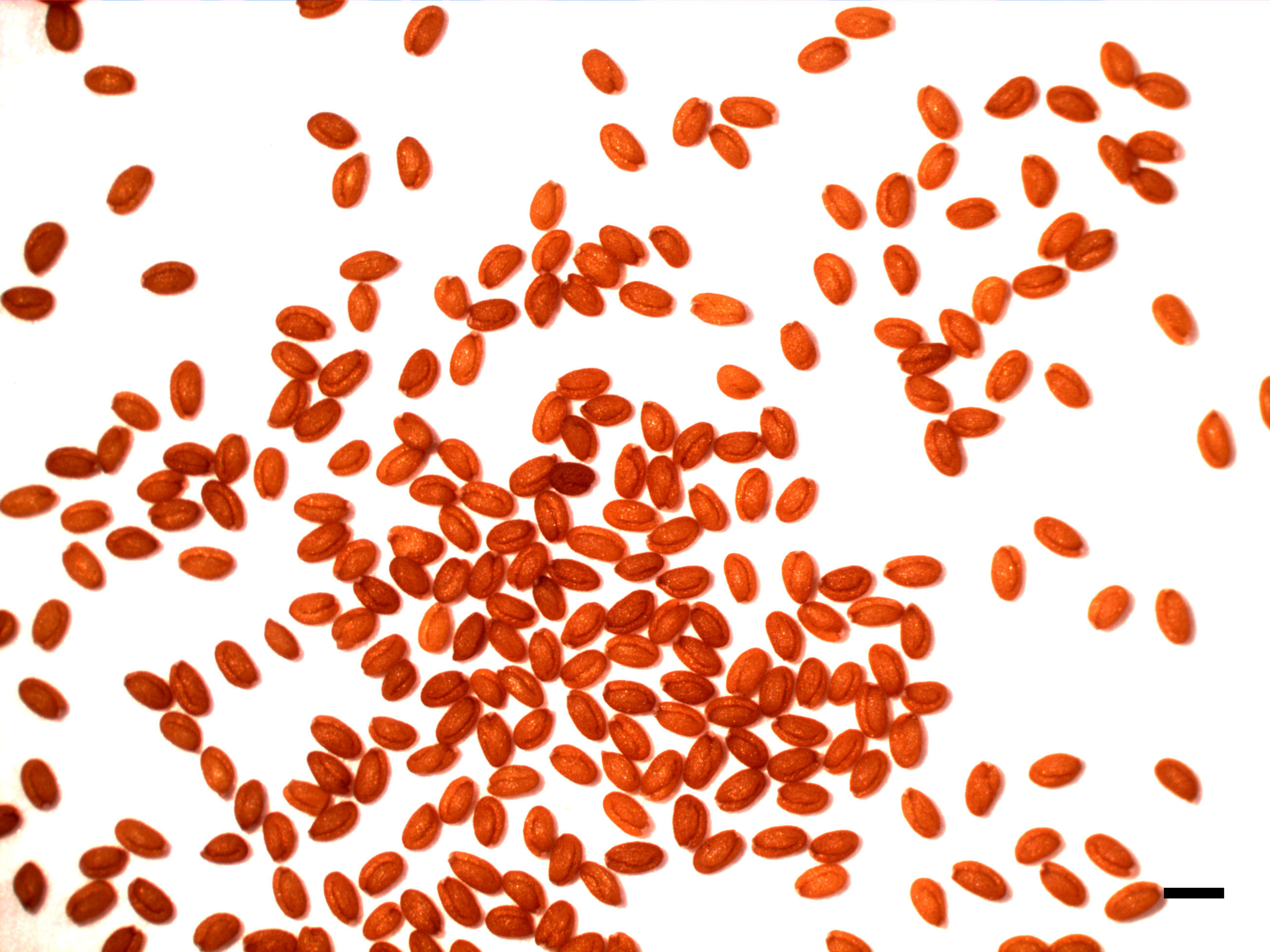

Supplement: Supplementary file 5 — Source Data [file 41467_2020_15603_MOESM5_ESM.zip › seed photos/myc3 myc4/myc3 myc4.jpg]

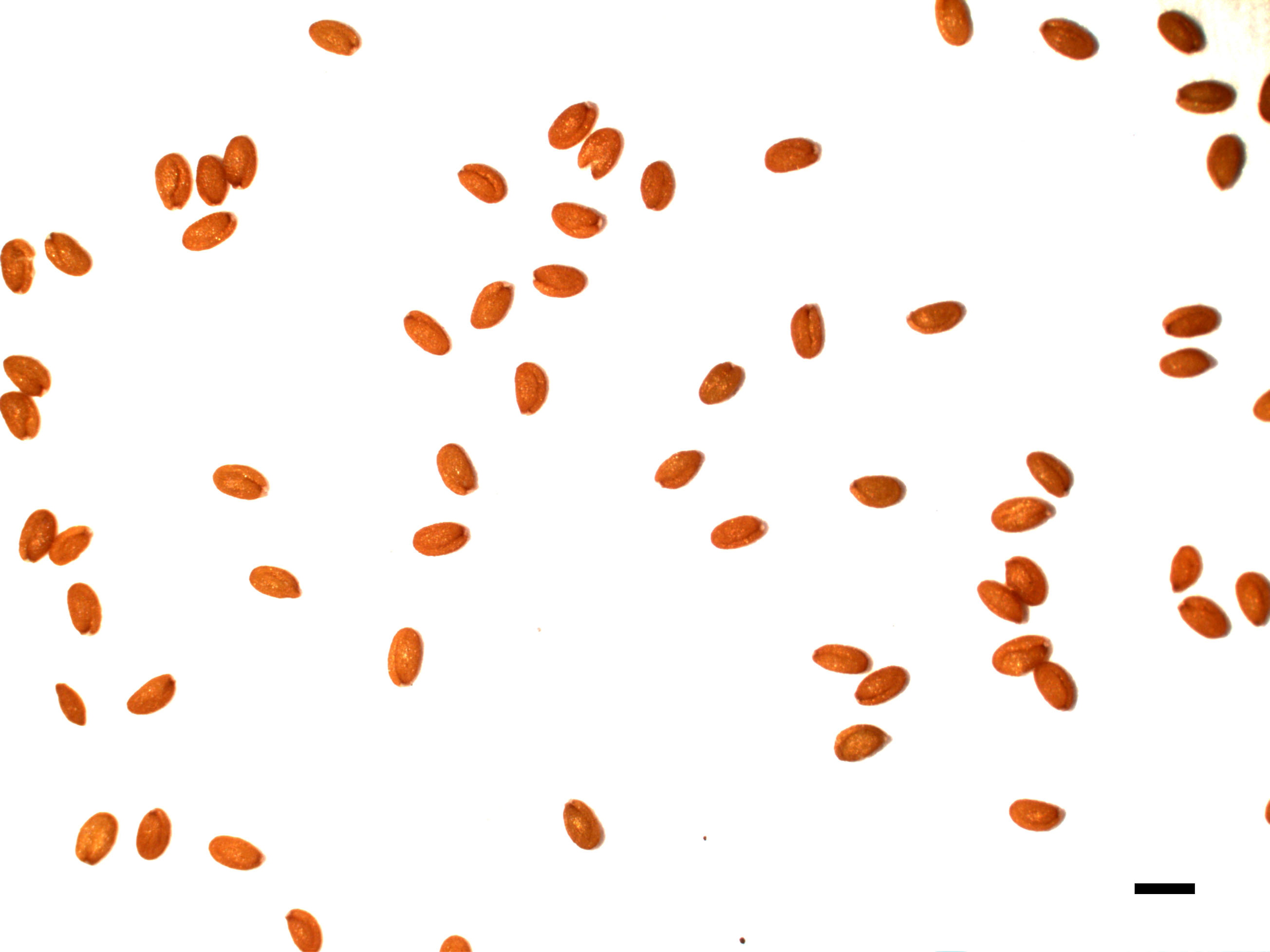

Supplement: Supplementary file 5 — Source Data [file 41467_2020_15603_MOESM5_ESM.zip › seed photos/myc3/myc3 #1.jpg]

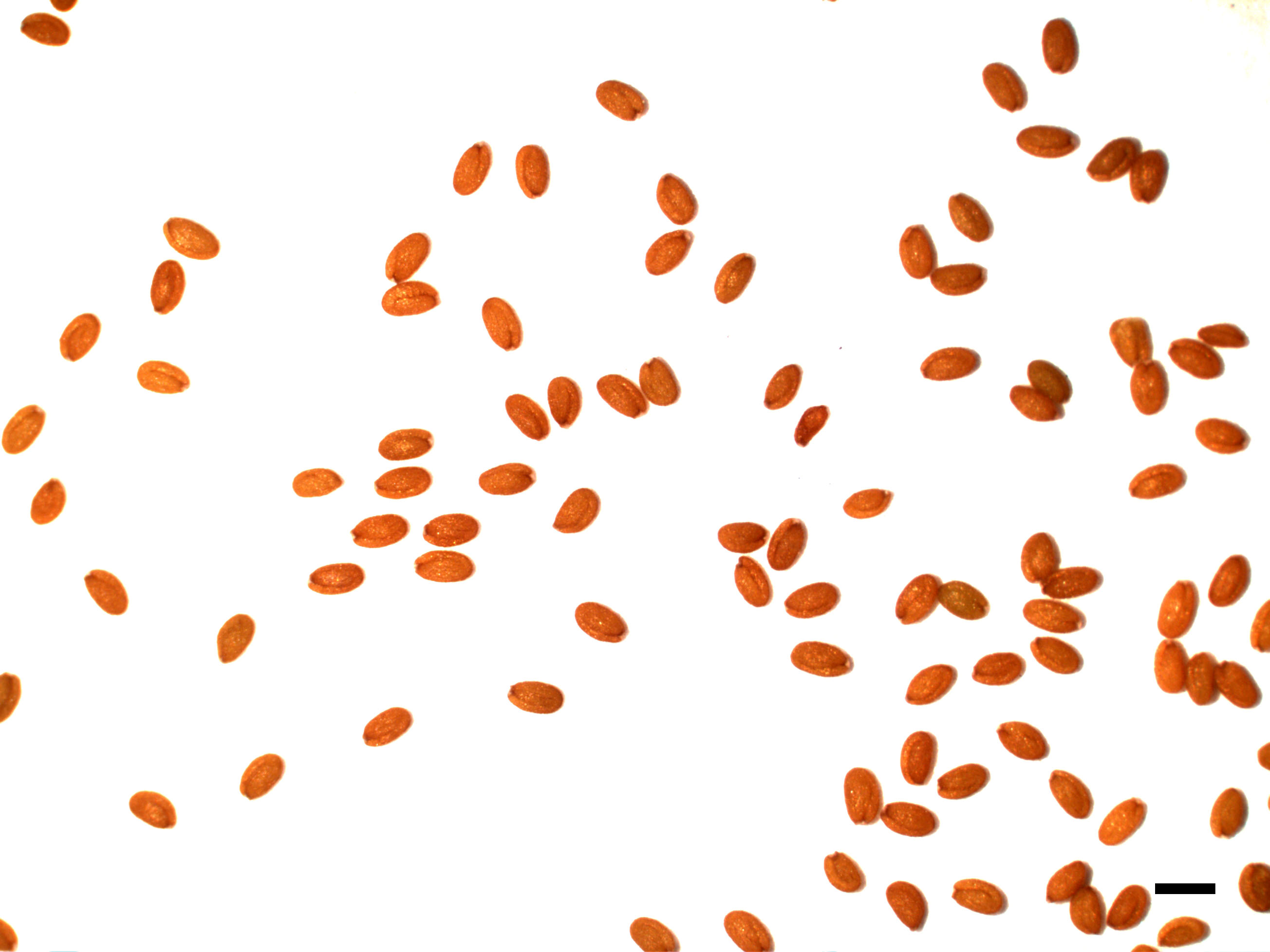

Supplement: Supplementary file 5 — Source Data [file 41467_2020_15603_MOESM5_ESM.zip › seed photos/myc3/myc3 #2.jpg]

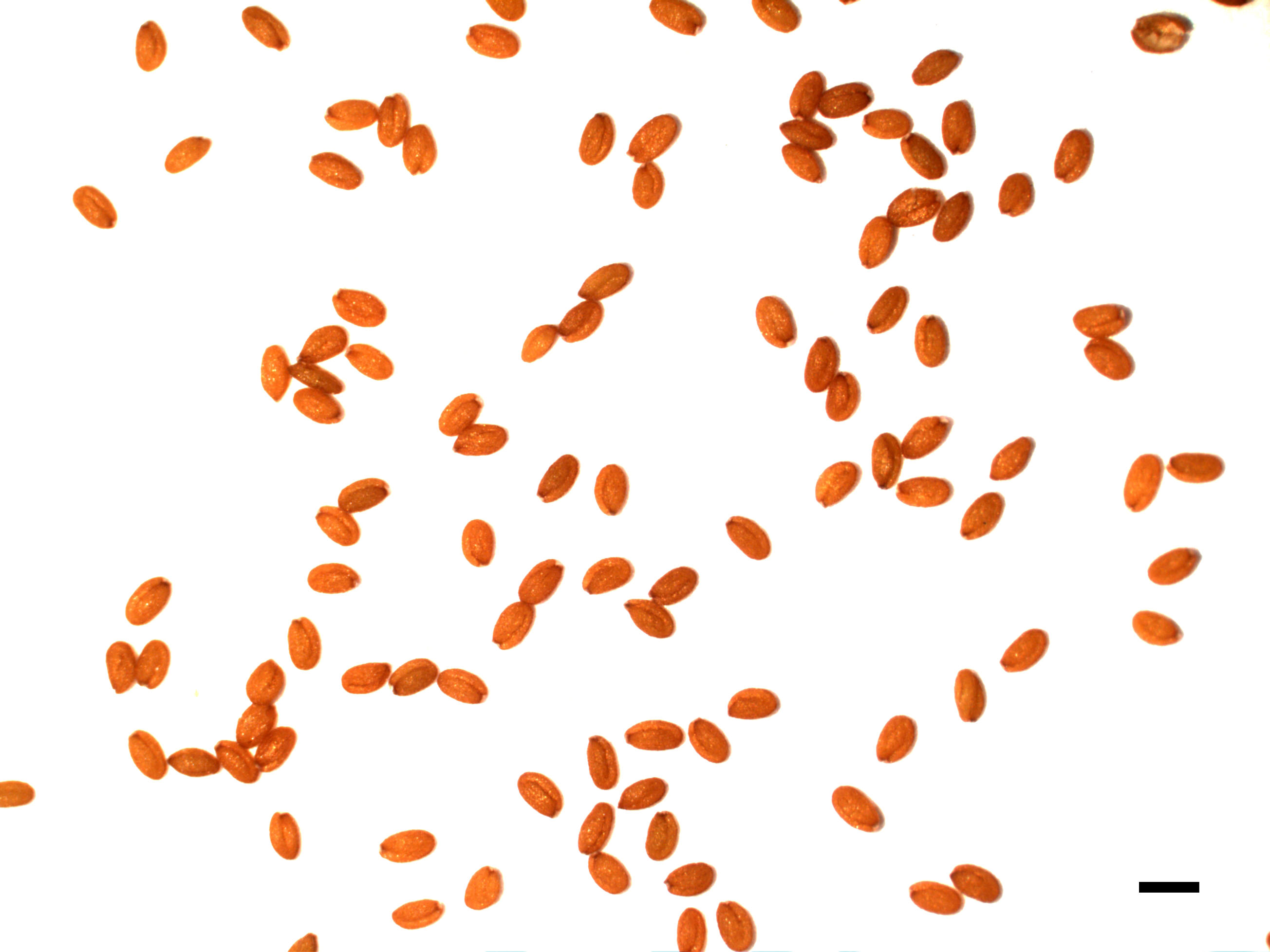

Supplement: Supplementary file 5 — Source Data [file 41467_2020_15603_MOESM5_ESM.zip › seed photos/myc4/myc4 #1.jpg]

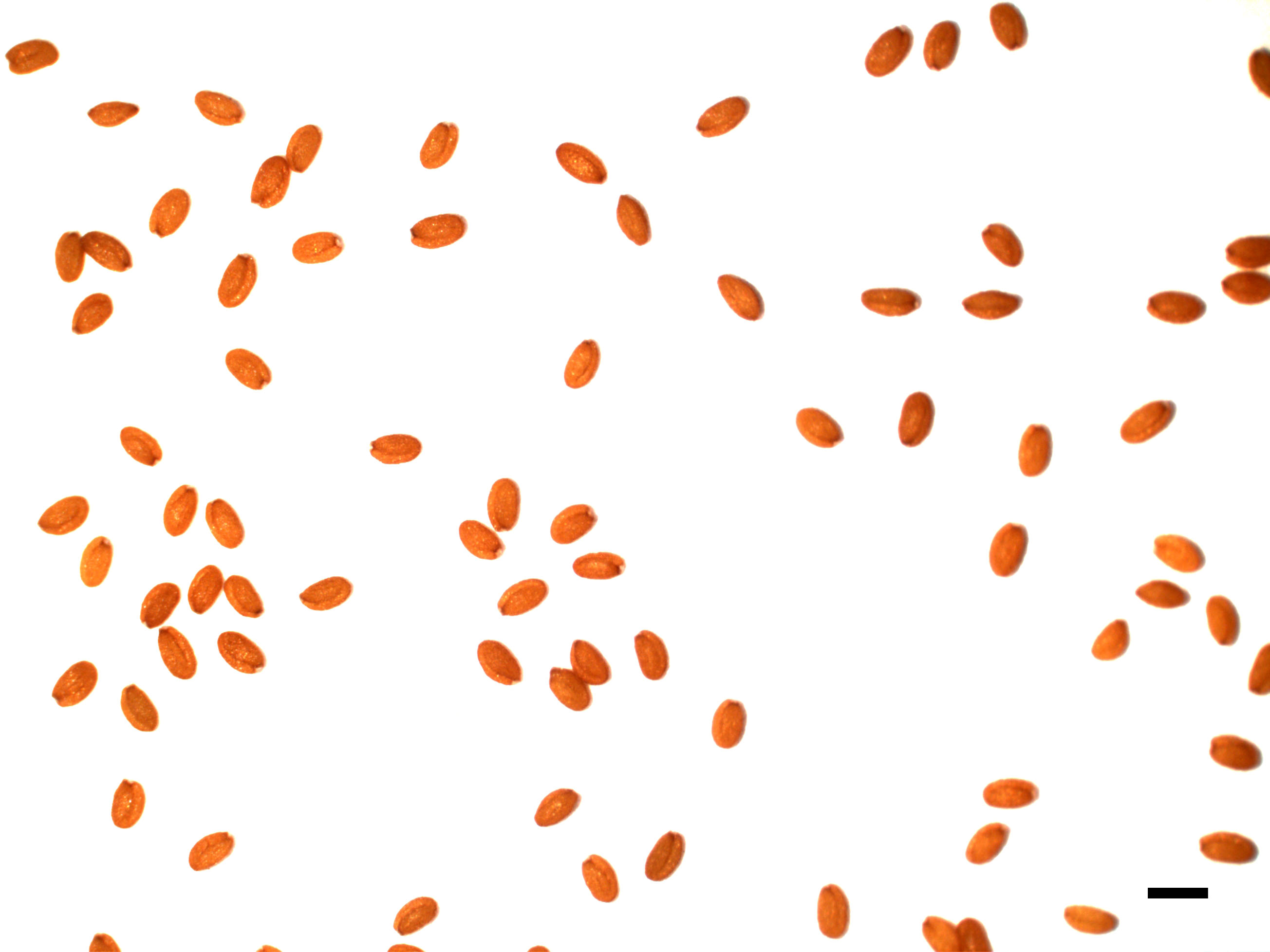

Supplement: Supplementary file 5 — Source Data [file 41467_2020_15603_MOESM5_ESM.zip › seed photos/myc4/myc4 #2.jpg]

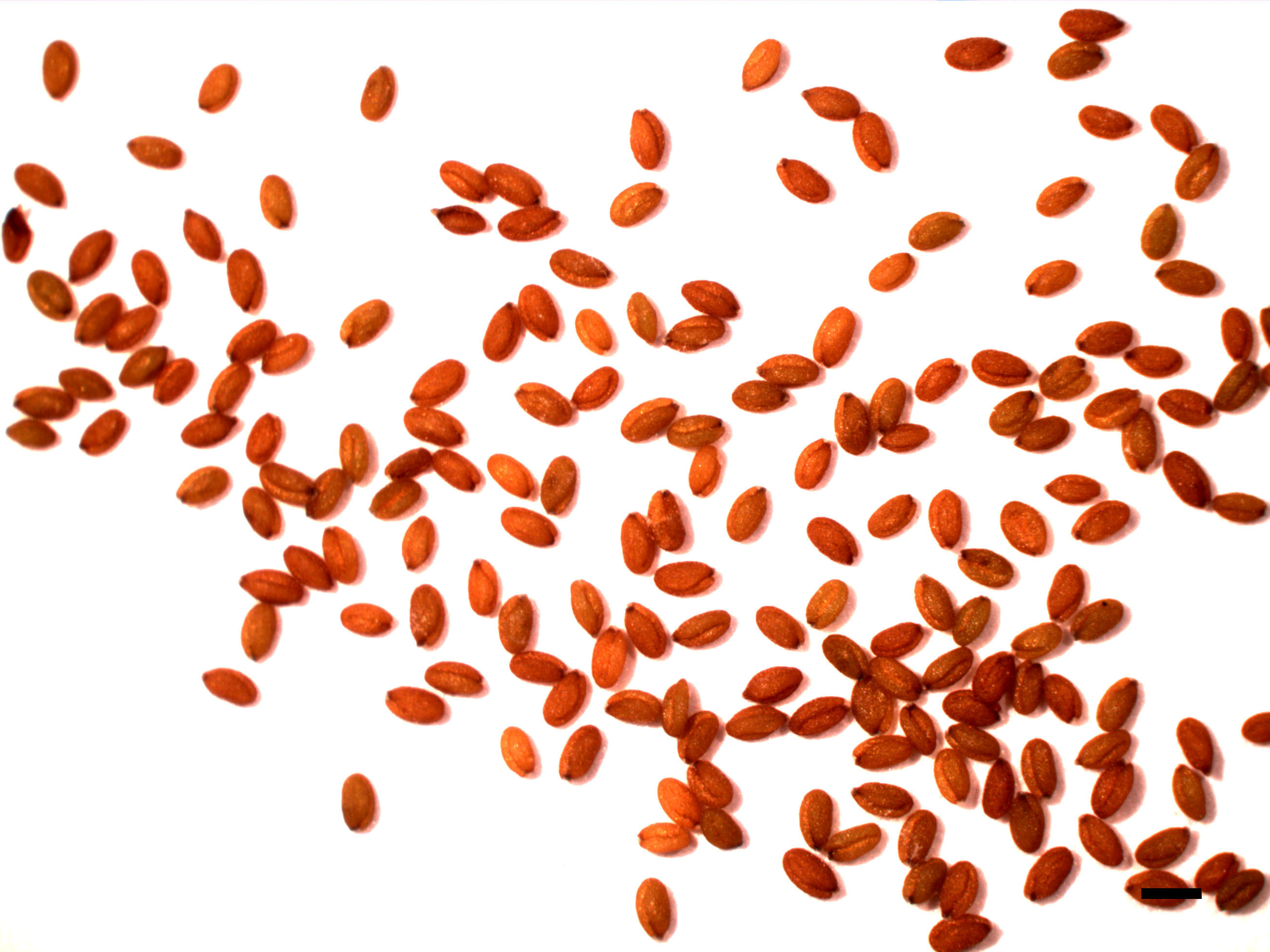

Supplement: Supplementary file 5 — Source Data [file 41467_2020_15603_MOESM5_ESM.zip › seed photos/ppd1-2 ppd2-cr/ppd1-2 ppd2-cr.jpg]

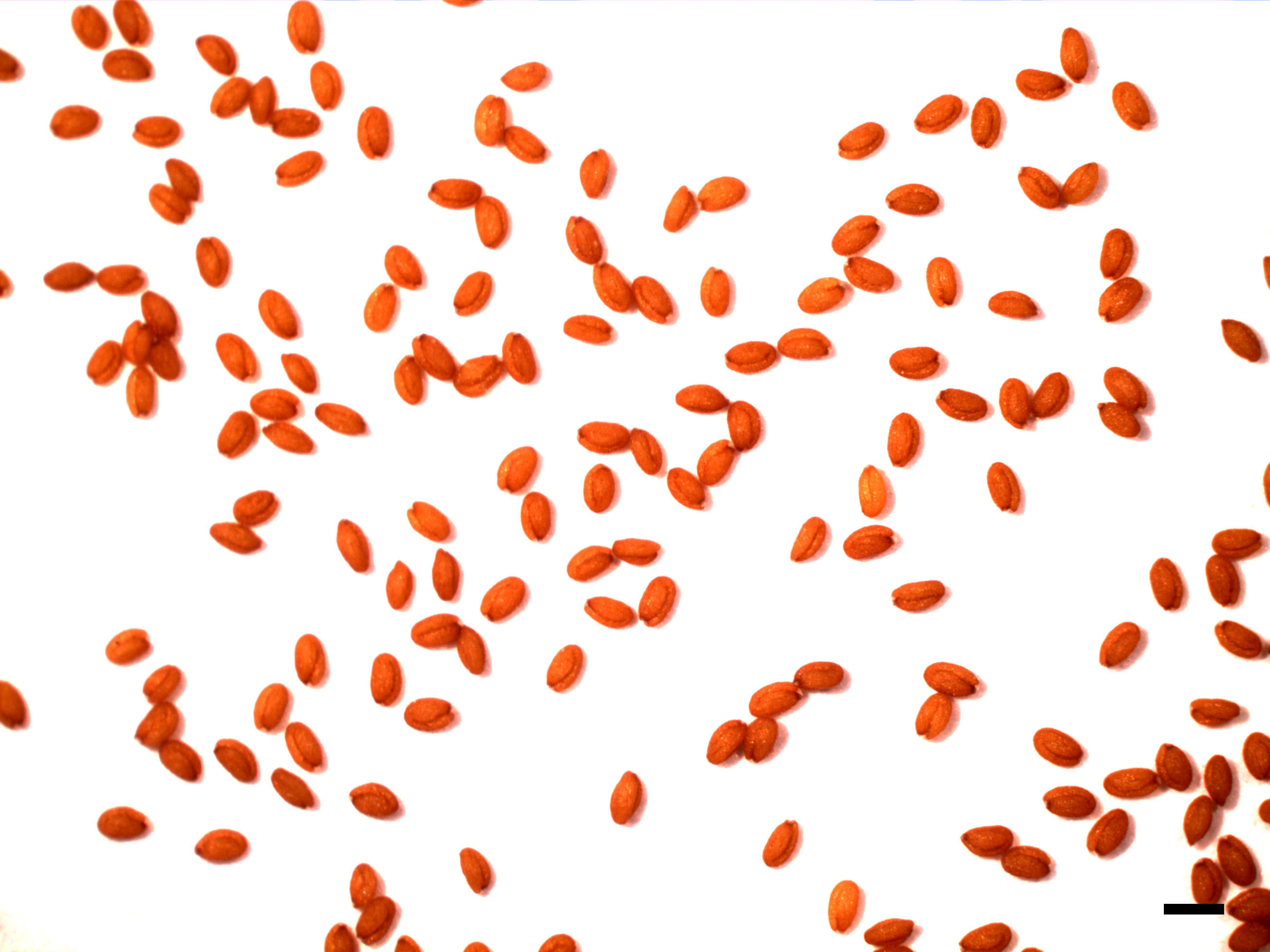

Supplement: Supplementary file 5 — Source Data [file 41467_2020_15603_MOESM5_ESM.zip › seed photos/ppd1-2/ppd1-2.jpg]

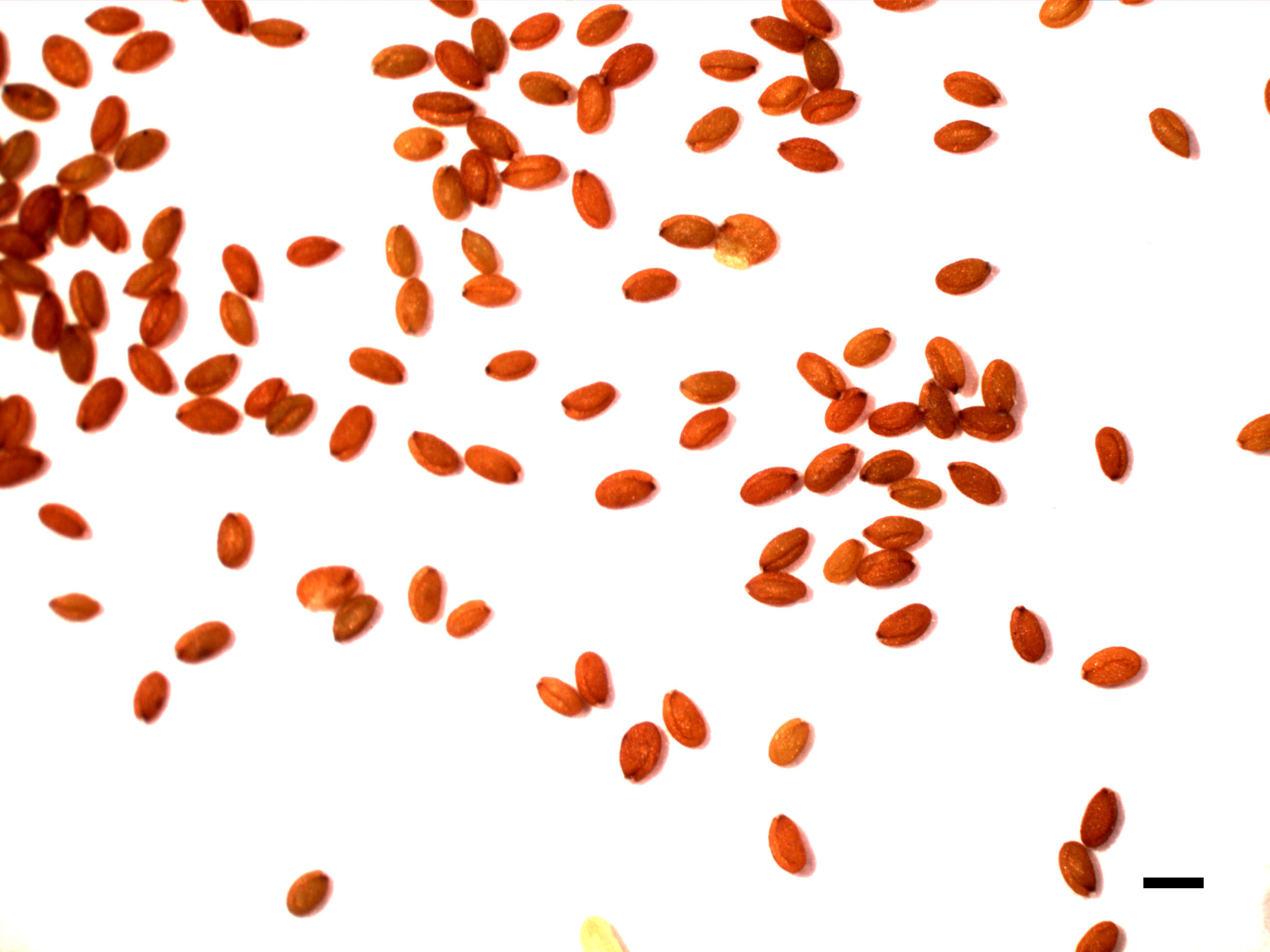

Supplement: Supplementary file 5 — Source Data [file 41467_2020_15603_MOESM5_ESM.zip › seed photos/ppd1-cr ppd2-1/ppd1-cr ppd2-1 #1.jpg]

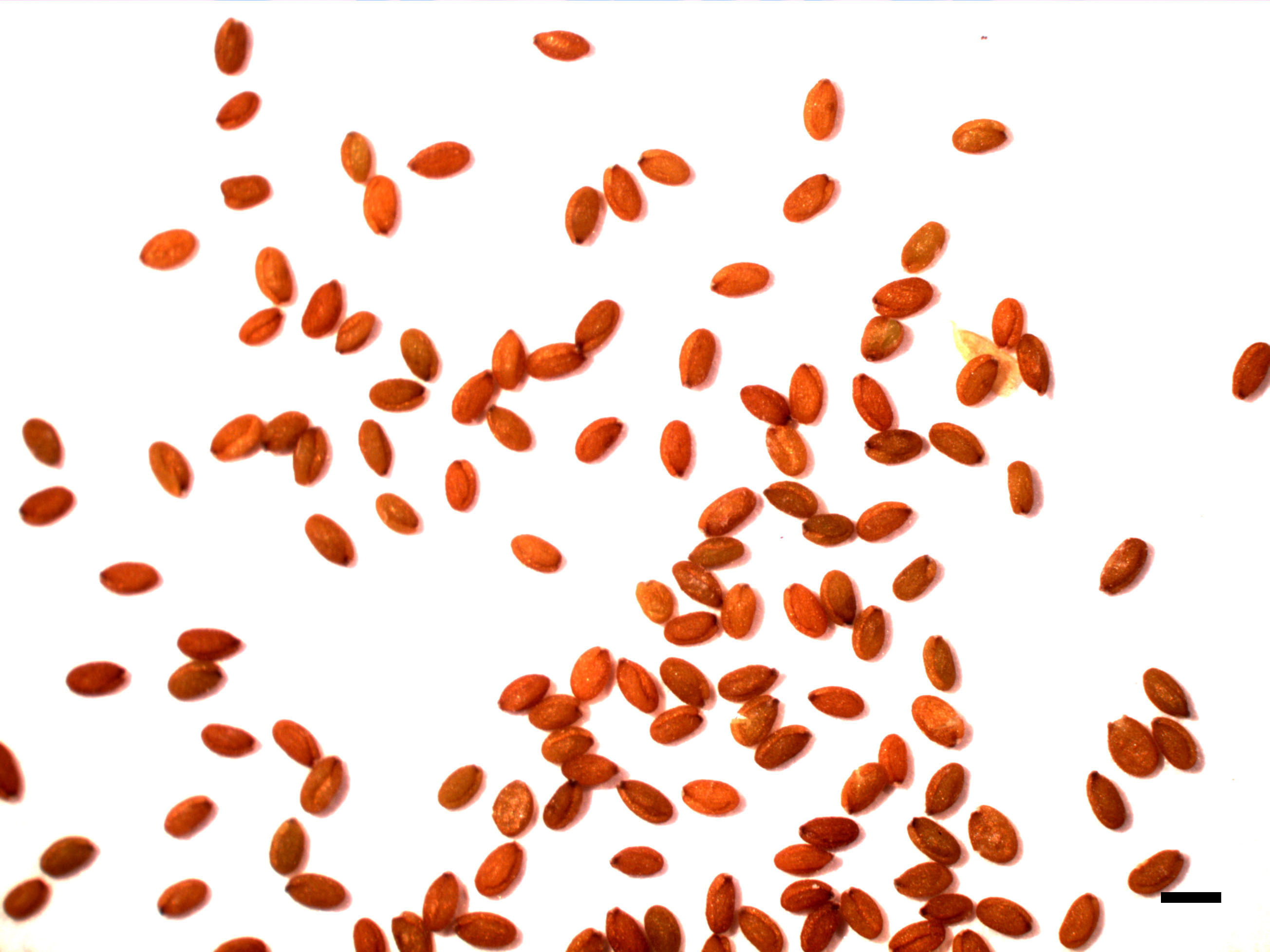

Supplement: Supplementary file 5 — Source Data [file 41467_2020_15603_MOESM5_ESM.zip › seed photos/ppd1-cr ppd2-1/ppd1-cr ppd2-1 #2.jpg]

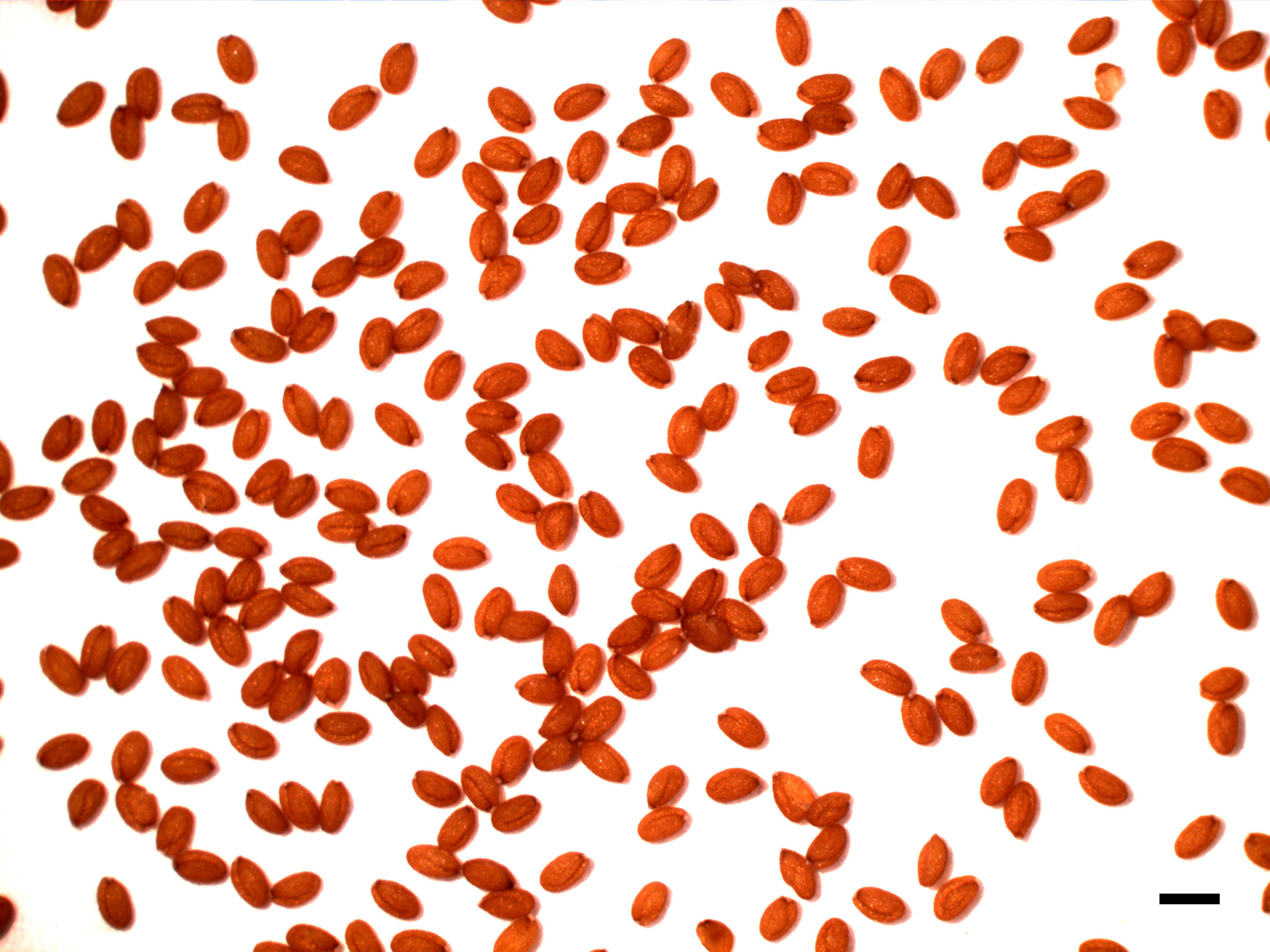

Supplement: Supplementary file 5 — Source Data [file 41467_2020_15603_MOESM5_ESM.zip › seed photos/ppd2-1/ppd2-1.jpg]
